# FKBP10 promotes M2 polarization of macrophage via MEK/ERK/CXCL8 axis and facilitates tumor progression in clear cell renal cell carcinoma

**DOI:** 10.7150/ijbs.117535

**Published:** 2026-01-15

**Authors:** Jin-Wei Chen, Jia-Ying Li, Hao-Qian Feng, Liang-Min Fu, Xin-Wei Zhou, Han-Sen Lin, Ying-Han Wang, Ke-Zhi Liu, Yu-Hang Chen, Zhu Wang, Qiong Deng, Jie-Yan Wang, Mei-Yu Jin, Hui Liang, Jin-Huan Wei, Jun-Hang Luo, Cheng-Peng Gui

**Affiliations:** 1 Department of Urology, The First Affiliated Hospital, Sun Yat-sen University, Guangzhou, Guangdong, China.; 2 Department of Urology, The First Affiliated Hospital, Guangzhou Medical University, Guangzhou, Guangdong, China.; 3 Department of Urology, The Second Xiangya Hospital, Central South University, Changsha, Hunan, China.; 4 Key Laboratory of Diabetes Immunology, Ministry of Education, National Clinical Research Center for Metabolic Disease, Changsha, Hunan, China.; 5 Department of Urology, Affiliated Longhua People's Hospital, Southern Medical University, Shenzhen, Guangdong, China.; 6 Institute of Precision Medicine, First Affiliated Hospital, Sun Yat-sen University, Guangzhou, China.

**Keywords:** FKBP10, clear cell renal cell carcinoma, tumor heterogeneity, tumor microenvironment, anti-PD-1/PD-L1 therapy

## Abstract

The progression and therapeutic response of clear cell renal cell carcinoma (ccRCC) are critically shaped by the complex interactions between tumor cell heterogeneity and the tumor immune microenvironment (TIME). However, a comprehensive classification of the ccRCC ecosystem and its clinical relevance is lacking. To address this, we utilized comprehensive bioinformatics approaches to analyze ten public single-cell RNA sequencing datasets from 194 samples across 118 ccRCC patients. Across 1,172,154 cells, we identified four TIME subtypes (immune activation, innate immunity, immunosuppressive myeloid [ISM], and immune exclusion) and six functional states of tumor cells (metabolic, angiogenic, stress-responsive, antigen-presenting, cell cycling, and epithelial-mesenchymal transition [EMT]). The interplay between these components defined four immune ecosystems, among which the ISM subtype, coupled with the EMT tumor state was associated with the poorest prognosis. Using machine learning-based prognostic modeling, we highlighted FKBP10 as a critical prognostic gene. Mechanistically, we demonstrated that FKBP10 not only promoted EMT but also activated the MEK/ERK/ELF3 signaling axis, leading to an increased secretion of CXCL8 by tumor cells. Tumor-derived CXCL8, in turn, drove macrophage M2 polarization and myeloid-derived suppressor cell (MDSC) recruitment, thereby reinforcing an immunosuppressive TIME. Furthermore, targeting FKBP10 synergized with anti-PD-1 therapy in suppressing tumor growth *in vivo*. Our work provides a comprehensive molecular atlas of the ccRCC ecosystem, establishes FKBP10 as a key regulator of immune suppression, and highlights its potential as a therapeutic target for personalized immunotherapy.

## Introduction

Clear cell renal cell carcinoma (ccRCC) is the most common and aggressive subtype of kidney cancer 1. Localized ccRCC shows surgical curability, but metastatic disease remains refractory to conventional chemotherapy. Although immune checkpoint blockade (ICB) has improved outcomes for a subset of patients 2-4, durable responses remain limited to about 30-40% of patients 5-7. This therapeutic plateau emphasizes the critical need to better understand the mechanisms of immune evasion in the tumor microenvironment (TME).

The ccRCC TME is a dynamic ecosystem composed of heterogeneous tumor cells and stromal components. Recent multi-omic and single-cell transcriptomic studies have begun to reveal this complexity, uncovering distinct molecular subtypes 2, 8-12, immune cell states 13-20, and spatial architectures 21, 22. However, two critical limitations persist. First, many current analyses treat the tumor and its microenvironment as separate entities, ignoring their dynamic co-evolution and the integrated network of cross-talk that functionally defines the tumor ecosystem 19, 23. Furthermore, previous studies have not connected TME subtypes to immunotherapeutic responses in ccRCC. This gap limits a comprehensive understanding of ccRCC biology and constrains the development of ecosystem-level prognostic biomarkers and therapeutic strategies.

To address this gap, we propose an integrated framework that combines the composition of the TIME, the states of malignant cells, and intercellular communication networks to define stable, clinically relevant tumor ecosystems in ccRCC. This study aims to decode the ccRCC ecosystem by integrating multiple single-cell transcriptomic datasets. Our specific objectives were: (1) to define coherent TIME subtypes and epithelial cell states; (2) to integrate these components into comprehensive tumor ecosystems; (3) to establish a prognostic model based on ecosystem-specific gene signatures; and (4) to identify and experimentally validate key molecular drivers in high-risk ecosystem.

Our investigation identified FK506 binding protein 10 (FKBP10) as a key candidate gene associated with high-risk ecosystems. FKBP10 is a member of FKBP family which contains a characteristic active peptidyl prolyl isomerases (PPIase) domain. PPIase is responsible for catalyzing the interconversion of cis/trans prolyl conformations, thus inducing rate-limiting change in protein conformation 24. Members of this family are associated with several cellular processes, including protein folding, stability and trafficking, kinase activity, and receptor signaling 25. FKBP family is important in regulating signaling pathways involved in inflammation, adaptive immune response, cancer, and developmental biology 26. Although prior studies have linked FKBP10 to other cancers 27-30, its functional role, particularly in reprogramming the ccRCC immune microenvironment, remains unexplored. Our mechanistic investigations show that FKBP10 promotes tumor metastasis through the MEK/ERK/ELF3 signaling axis, stimulating CXCL8 secretion that in turn drives M2 macrophage polarization and MDSC recruitment. Thus, our work not only presents a comprehensive atlas of the ccRCC ecosystem but also highlights FKBP10 as a central mediator of immunosuppressive niche formation, offering a novel therapeutic target for this aggressive malignancy.

## Methods

### Data Availability

The entire study design is succinctly illustrated in a detailed flowchart (Supplementary **Fig. S1**). Detailed information about resources and reagents is provided in Supplementary **Table S1.** The acquisition of single-cell RNA-sequencing (scRNA-seq) datasets was facilitated from sources including: GSE131685 18, GSE159115 17, GSE207493 19, GSE224630 31, SRZ190804 21, Young *et al.* 16, Braun *et al.* 13, Bi *et al.* 32, Li *et al.* 33, Obradovic *et al.* 34. The last three datasets were used as external datasets and used for validating our main findings. The spatial transcriptomic data analyzed in this study, including the H&E-stained tissue image, were obtained from the publicly available dataset published by Meylan *et al.* 22. Bulk RNA-seq datasets were procured from The Cancer Genome Atlas (TCGA-KIRC cohort) and ArrayExpress (E-MTAB-1980) 35. Additionally, datasets from ICB-treated/CAR-T-treated cohorts were obtained: CheckMate cohorts, detailed in Braun *et al.* 23; JAVELIN Renal 101, documented by Motzer *et al.* 36; IMvigor210 cohort 37; VanAllen cohort 38; Kim cohort (GSE135222); Cho cohort (GSE126044); Hugo cohort (GSE78220); and Lauss cohort (GSE100797).

### Multicenter Cohort and Bulk RNA Sequencing

This study utilized surgical specimens from a multicenter cohort of 61 patients with ccRCC treated with ICB-tyrosine kinase inhibitor (TKI) combination therapies. Fresh tumor tissues were prospectively collected from four Chinese medical institutions: First Affiliated Hospital of Sun Yat-sen University (Guangzhou, China; n = 12): Axitinib-Toripalimab (n = 9), Axitinib-Pembrolizumab (n = 2), Lenvatinib-Pembrolizumab (n = 1); Sun Yat-sen University Cancer Center (Guangzhou, China; n = 21): Axitinib-Toripalimab (n = 12), Axitinib-Pembrolizumab (n = 6), Lenvatinib-Pembrolizumab (n = 3); Renji Hospital, Shanghai Jiao Tong University (Shanghai, China; n = 13): Axitinib-Toripalimab (n = 13); Shengjing Hospital, China Medical University (Shenyang, China; n = 15): Axitinib-Toripalimab (n = 15). Specimens were obtained during surgery, immediately snap-frozen in liquid nitrogen, and stored at -80℃ until RNA extraction.

Bulk RNA-seq libraries were prepared via the TruSeq Stranded mRNA Library Prep Kit (Illumina) and sequenced on an Illumina NovaSeq 6000 platform. Raw reads were processed through FastQC for quality control, followed by adapter trimming and filtering with Trimmomatic. Clean reads were aligned to the GRCh38 human genome using STAR, and gene expression counts were quantified via featureCounts.

### Identification of Cellular Modules and TIME Subtypes

To examine the cellular composition and heterogeneity within TIME, we investigated the co-existence patterns of different cell subpopulations 39. Pairwise correlation values between the normalized frequencies of any two clusters within individual tumor samples were quantified using the corr.test function. These resulting correlation coefficients were subjected to hierarchical clustering employing the pheatmap package in R, utilizing the Ward.D2 clustering method and correlation distance as metrics. To avoid potential distortion of clustering due to the limited cell number of certain clusters, samples that contained fewer than 1,000 non-epithelial cells were excluded from this analysis. For each patient, the cluster-normalized frequencies of clusters from the same cellular module were summed, and the most abundant cellular module was designated as the dominant cellular module for this patient. Each cellular module corresponds to a TIME subtype, in which the phenotype was designated based on four aspects: (1) cellular composition, (2) marker genes expression and KEGG pathways enrichment, (3) TIME-related gene signatures as previously described (**Table S2**) 22, 40, (4) prognostic relevance verified with BisqueRNA that predicted cell type composition in bulk expression 41. Finally, we identified and listed the top 10 marker genes for the five most prevalent cell types within each module. These marker genes were defined as the molecular signature for specific cellular modules.

### Classification of Intra-tumoral Gene Expression Programs

Consensus non-negative matrix factorization (cNMF) was employed to identify gene expression programs (GEPs) in tumor samples 42. Samples with fewer than 100 tumor cells were filtered out, and 43 tumor samples were selected for this analysis. For each sample, cells with fewer than 300 unique genes and genes detected in fewer than 5 cells were filtered out. Then we selected 2,000 genes with the most over-dispersion, as determined by the v-score 43. Each gene was scaled to unit variance before running cNMF. We used cNMF with default parameters, except for the maximum number of iterations (=200). We adjusted the number of NMF components (k-value) by comparing the trade-off between predictive accuracy and solution stability, as described by Alexandrov *et al.* 44. We then obtained two matrices for each sample: one is the usage matrix (cells×programs) that captured normalized program usage in each cell (i.e., the proportion of the cell's transcripts attributed to each program), and the other is the GEP matrix (genes×programs) that listed the top-ranked genes within the programs according to their loadings of the NMF factor. We retained a total of 250 GEPs whose average cell usage was larger than 0.01 according to the Program-Ratio plots. The 250 programs were then compared by hierarchical clustering, using one minus the Pearson correlation coefficient over all gene scores as a distance metric. Six clusters of signatures were manually identified and used to define meta-programs (MPs). For each MP, we combined the top 100 genes of each GEP and calculated the average loading for each gene. We summarized the total loadings for repetitive genes, retained the original loadings for exclusive genes, and divided the loadings for each gene by the number of programs within the MP. Finally, the top 30 genes with the highest loading were identified as MP marker genes.

The functions of MPs were defined based on hallmark pathway analysis using the GSVA, and prognostic relevance. Prognostic associations were determined based on the predicted cell type composition in bulk expression by CIBERSORTx.

### Jaccard Similarity Analysis

The Jaccard similarity index was calculated to quantify the transcriptional resemblance between six MPs of malignant cells and the signatures of 12 cell types from PanglaoDB (https://panglaodb.se/) (**Table S3**). The Jaccard index was calculated using the top 50 marker genes with the following formula:

J (A, B) = |A Ո B| / |A U B|

### Cell-Cell Interaction Analysis

CellphoneDB facilitated the investigation of ligand-receptor pairs, highlighting significant interactions after adjusting for frequency constraints below 0.1% or above 2% of all cluster-cluster combinations 39, 45. The total number of ligand-receptor pairs among the different clusters within the same ecosystem was counted. We assumed that a ligand-receptor pair was enriched in a specific ecosystem if R_o/e_ > 1. First, the expected count of each ligand-receptor pair was calculated using the *χ^2^* test. Second, using Epitools, we calculated the R_o/e_ value according to the following formula:



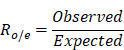



To identify the potential ligands of myeloid cells that drive the unique phenotype of EMT tumor cells, we used the signature of EMT for NicheNet analysis 46.

### Establishment of Ecosystem Specific Signature and Machine Learning-based Prognostic Models

An ecosystem is defined as a distinct entity formed by the hierarchical clustering of malignant cell states and TIME subtypes, which reveals functional interplay patterns among these components. Ecosystem-specific signatures were established by integrating cellular module signatures, corresponding MP signatures, and the ten ligand-receptor pairs with the highest R_o/e_ within the ecosystem. The enrichment score of ecosystem-specific signatures in bulk RNA-seq was estimated by ssGSEA. The signature from the most prognostically unfavorable ecosystem was selected to construct prognostic models.

To develop a consensus immunosuppressive myeloid and epithelial-mesenchymal transition interactive signature (ISM-EMT-Sig) with superior accuracy and stability, we integrated 11 machine learning algorithms according to previous studies 47-50. The integrative algorithms encompassed LASSO regression, Elastic Net (Enet), ridge regression, Random Survival Forest (RSF), CoxBoost, Stepwise Cox regression, Supervised Principal Component Analysis (SuperPC), Partial Least Squares Regression for Cox models (plsRcox), Survival Support Vector Machine (survival-SVM), generalized boosted regression Modeling (GBM), and Kang score. The Kang score, in particular, was generated using an approach similar to that used by Kang *et al.* 51 and Wang *et al.* 52. Specifically, algorithms such as LASSO, Kang score, RSF, StepCox, and CoxBoost are capable of variable screening. This enables us to use these five algorithms for variable selection and subsequently combine them with the remaining algorithms to build a prognostic model. Alternatively, we can use any of the 11 algorithms independently to construct the model. Ultimately, this methodology allows us to generate a total of 126 models.

The signature was generated as follows: (a) Univariate Cox regression identified prognostic mRNAs in the TCGA-KIRC cohort; only the genes with an expression fold change > 1.5 or < -1.5 and an FDR < 0.01 were selected for subsequent analysis. Subsequent evaluation of the selected differentially expressed genes (DEGs) for their statistical association with patient survival using a univariate Cox proportional hazards regression model, prioritizing DEGs that align with survival trends (e.g., DEGs highly expressed in the ecosystem 3 group indicating a hazard ratio (HR) > 1 or those prevalent in other groups with a HR < 1); (b) 126 algorithm combinations were applied to these prognostic mRNAs to construct predictive models within the TCGA-KIRC cohort (training set: 70%, internal validation set: 30%; (c) All models were evaluated across four validation datasets (E-MTAB-1980, CheckMate, JAVELIN, and SYSUFAH). (d) For each model, the Harrell concordance index (C-index) was calculated across all validation datasets. Besides, the number of genes included in each model was defined as model size. Considering the accuracy of the model and its simplicity, we aimed to include as few genes as possible (within 15 genes) to achieve the best prediction effect. Therefore, the model with the highest average C-index across validation datasets, incorporating no more than 15 genes, was selected as the optimal prognostic tool.

### Colony Formation Assay

The cells were harvested at 70% confluence and seeded into the 6-well plates. Each cell line was seeded in triplicate. After 2 weeks of culture, cells were fixed with formaldehyde for 15 minutes. The cells were then stained with crystal violet staining solution for 30 minutes. The colonies were imaged using Amersham Imager 600 imaging system, and were counted and analyzed using ImageJ software.

### Cell Proliferation Assay

The cells were counted and seeded in the 96-well plate for 6 days. After washing the cells with PBS, CCK8 was added to RPMI-1640 (10:90) every 24 hours. After incubation with CCK8 for 2 hours, absorbance at 450 nm was measured using a microplate reader.

### Transwell Assay

Matrigel (diluted with sterile deionized ice-cold water in a 1:2 ratio) was added to the membrane of the transwell insert, and the plate was incubated for 1 hour. The cells were then seeded onto the membrane of a 24-well transwell insert. Afterward, the migration buffer containing chemoattractant was added below the 24-well transwell insert and the plate was incubated for 24 hours. Remove the Matrigel by gently using a cotton tipped applicator which was infiltrated with PBS. Formaldehyde was added to the 24-well plate for 30 minutes. After washing the transwell inserts, crystal violet staining solution was used for cell staining. After 10 minutes, the transwell inserts were washed three times with PBS. When dried out, the transwell insert was observed under a microscope and imaged by OLYMPUS IX83 inverted imaging system 53. Migrating cells were counted and analyzed using ImageJ software.

### Subcutaneous Tumorigenesis

For the subcutaneous tumor model, 769-P human RCC cells stably transfected with lentiviral shRNA negative control (shNC) and shFKBP10 or vehicle and over-expression of DNASE1L3/DPEP1 were subcutaneously implanted into 4-week-old male nude mice (2×10^6^ cells/mouse).

To investigate the roles of FKBP10 and CXCL8, 769-P cells (2×10^6^ cells/mouse) stably transfected with shNC or shFKBP10 were implanted into 4-week-old male BALB/c mice, with or without CXCL8 injections every 3 days. Additionally, 769-P cells with lentiviral vehicle or FKBP10 overexpression were implanted into BALB/c mice. One week post-implantation, IgG (0.1 mg/mouse), CXCL8 neutralizing antibodies (0.1 mg/mouse), or CXCR1/2 inhibitor reparixin (30 mg/kg) in PBS were injected intraperitoneally every 2 days for 3 weeks.

Tumor size and volume were calculated as tumor volume (mm^3^) = (longest diameter) × (shortest diameter)^2^ × 0.5. At the endpoint, tumors were harvested and weighed.

### Immunofluorescence Staining

Immunofluorescence staining was performed on FFPE ccRCC sections following antigen retrieval and blocking procedures as described for immunohistochemistry. Sections were co-incubated with anti-CD86 (1:100) and anti-CD206 (1:200) overnight at 4℃. Species-specific secondary antibodies were applied for 1 hour in the dark: Alexa Fluor 488-conjugated Goat Anti-Mouse (1:500) and Alexa Fluor 555-conjugated Goat Anti-Rabbit (1:500). Nuclei were stained with DAPI for 5 minutes.

### Vesicle-like PLGA-based Nanoparticle (VNP) Formulation of siRNA Drug and Application of VNPsiRNA *In Vivo*

VNPsiRNA was prepared using a double emulsion method, as previously described 54. The 2'-O-Methyl (2'-OMe) modified siRNA was first mixed with chloroform, containing DOTAP and mPEG5k-b-PLGA11k (50:50), and emulsified by sonication on ice. This primary emulsion was then further emulsified in DEPC water through additional sonication on ice. The chloroform was subsequently removed using a rotary evaporator. The resulting nanoparticle dispersion was transferred to an ultrafiltration device and centrifuged to remove any unencapsulated compounds. The nanoparticles exhibited a spherical shape, with a particle size of 150 nm and an encapsulation efficiency of 49.2%. siRNA encapsulation efficiency was determined by high-performance liquid chromatography analysis. For syngeneic tumor models, Renca murine RCC cells were injected into 5- to 6-week-old male Balb/c mice (2×10^6^ cells/mouse), VNPsiRNA were administrated via intratumoral injection (40μg/mouse every 2 days) when tumors reached 50 mm^3^. Tumors were measured every alternate day and weighed upon harvesting.

### Cytokine Array Analysis

The cytokine secretion profiles in conditioned media from tumor cell cultures were analyzed using the Proteome Profiler Human Cytokine Array Kit following the manufacturer's protocol. Briefly, tumor cells treated with recombinant FKBP10 (1 μg/ml, 24 hours) were cultured in serum-free medium for 24 hours to eliminate exogenous protein interference. Conditioned media were collected, centrifuged to remove cellular debris, and aliquoted for immediate analysis. Array data were quantified by measuring the pixel density of duplicate spots using ImageLab 6.1 software. Background signal from negative control spots was subtracted, and relative cytokine levels were normalized to internal positive controls on each membrane. This method enabled simultaneous semi-quantitative screening of multiple cytokines, with sensitivity thresholds and cross-reactivity profiles as specified by the manufacturer.

### Embedded Co-culture Transwell Assay

THP-1 monocytes (human leukemia monocytic cell line) were cultured in RPMI-1640 complete medium supplemented with 10% FBS and 1% penicillin/streptomycin. Macrophage differentiation was induced by treating cell suspensions with 100 ng/mL phorbol 12-myristate 13-acetate for 24 hours. Following incubation, differentiated M0 macrophages were obtained through three gentle PBS washes to remove non-adherent cells, and were then cultured in fresh complete medium until reaching 80% confluency.

For the co-culture assay, Transwell inserts were placed into 24-well plates. M0 macrophages (2×10^4^ cells/well) were seeded into the lower chamber, while cancer cells (2×10^4^ cells/well) were plated in the upper chamber. In the experimental groups, recombinant human CXCL8 (20 ng/mL) was added to both chambers to establish a chemotactic gradient. Control groups received equivalent volumes of PBS. The co-culture system was maintained for 48 hours under standard culture conditions. Cell invasive capacity was assessed using the established Transwell protocol as previously described.

### Flow Cytometry

Tumors from mice were digested and prepared into single-cell suspension. Cells were incubated with antibodies for 30 minutes. The antibodies used included CD45, CD11b, F4/80, CD80, CD86, CD206, and Gr-1. Antibodies were used at ≤ 1.0 µg per million cells in a 100 µL volume. Data acquisition was performed using a Beckman CytoFLEX flow cytometer, and the collected data were analyzed with FlowJo v10.

### Prediction of Potential Transcriptional Factors

The putative promoter region of the CXCL8 gene was retrieved from the NCBI database (GRCh38.p14). Based on canonical promoter annotation principles, genomic sequence spanning 2,000 bp upstream of the transcription start site (TSS) and 100 bp downstream was defined as the promoter region. The genomic sequence was extracted and validated using the NCBI Genome Data Viewer.

To identify transcription factors (TFs) with potential binding activity in the CXCL8 promoter, the UCSC Genome Browser (https://genome.ucsc.edu/) was employed 55. Using the GRCh38/hg38 human reference genome assembly, the promoter coordinates (Chr4:73,738,569-73,740,669) were input into the search interface. Publicly available ChIP-seq and chromatin accessibility datasets were queried, and binding events were filtered to retain only those with a binding score ≥ 600. To prioritize clinically relevant TFs, differential expression analysis was performed using the GEPIA2 database (http://gepia2.cancer-pku.cn/) 56, focusing on TFs exhibiting significant positive correlations (*P* < 0.05, Pearson correlation) with CXCL8 expression.

For the candidate TFs identified in the preceding step, de novo motif scanning was conducted using JASPAR (http://jaspar.genereg.net/), a curated database of TF-binding profiles 57. A conservative relative profile score threshold of 90% (corresponding to a *P* < 1×10^-3^) was applied to minimize false-positive predictions. Predicted binding sites were visualized and annotated using JASPAR integrated analysis tools, with genomic coordinates and motif scores reported for all high-confidence hits.

### ChIP-seq

Cross-linked chromatin was prepared by treating cells with 1% formaldehyde, followed by quenching with 125 mM glycine. Chromatin was extracted using lysis buffer and sonicated to fragments ranging from 100 to 500 bp. Immunoprecipitation was performed overnight at 4 ℃ with ELF3 antibodies bound to Dynabeads. The immunoprecipitated DNA was purified and used for library construction with the NEBNext Ultra DNA Library Prep Kit for Illumina. Libraries were prepared through end repair, adapter ligation, USER enzyme digestion, and PCR amplification. Size selection was carried out using AMPure XP beads, and library quality was assessed with Qubit quantification and a high-sensitivity DNA chip. Sequencing was performed on the Illumina NovaSeq 6000 platform to generate 150 bp paired-end reads.

Raw sequencing reads were processed with fastp to remove adapters, poly-N sequences, and low-quality bases. Clean reads were aligned to the reference genome using Bowtie2. Peak calling was conducted with MACS2, and results were visualized with IGV. Peak annotation was performed using the ChIPseeker R package. Differential peak analysis was conducted using MAnorm.

### Statistical Analysis

R Project, RStudio, and Python were used for sequencing and machine learning analysis, while GraphPad Prism was used for the statistical analysis of experimental data.

For intergroup comparisons, a two-tailed Student's t-test (parametric, normal distribution) or Welch's t-test (unequal variances) was used. For non-normally distributed data, the Wilcoxon rank-sum test was applied. Paired t-tests were used for paired samples, and one-way ANOVA for multiple group comparisons. Chi-square or Fisher's exact tests were used for categorical data.

Survival analysis was performed using Kaplan-Meier curves and assessed with the Log-rank test. HR were calculated using the Cox proportional hazards model. Pearson or Spearman correlation coefficients were used depending on the data distribution. Statistical significance was set at *P* < 0.05.

## Results

### Enrichment of Myeloid Cells in Immunotherapy-Resistant ccRCC

**Figure 1:**
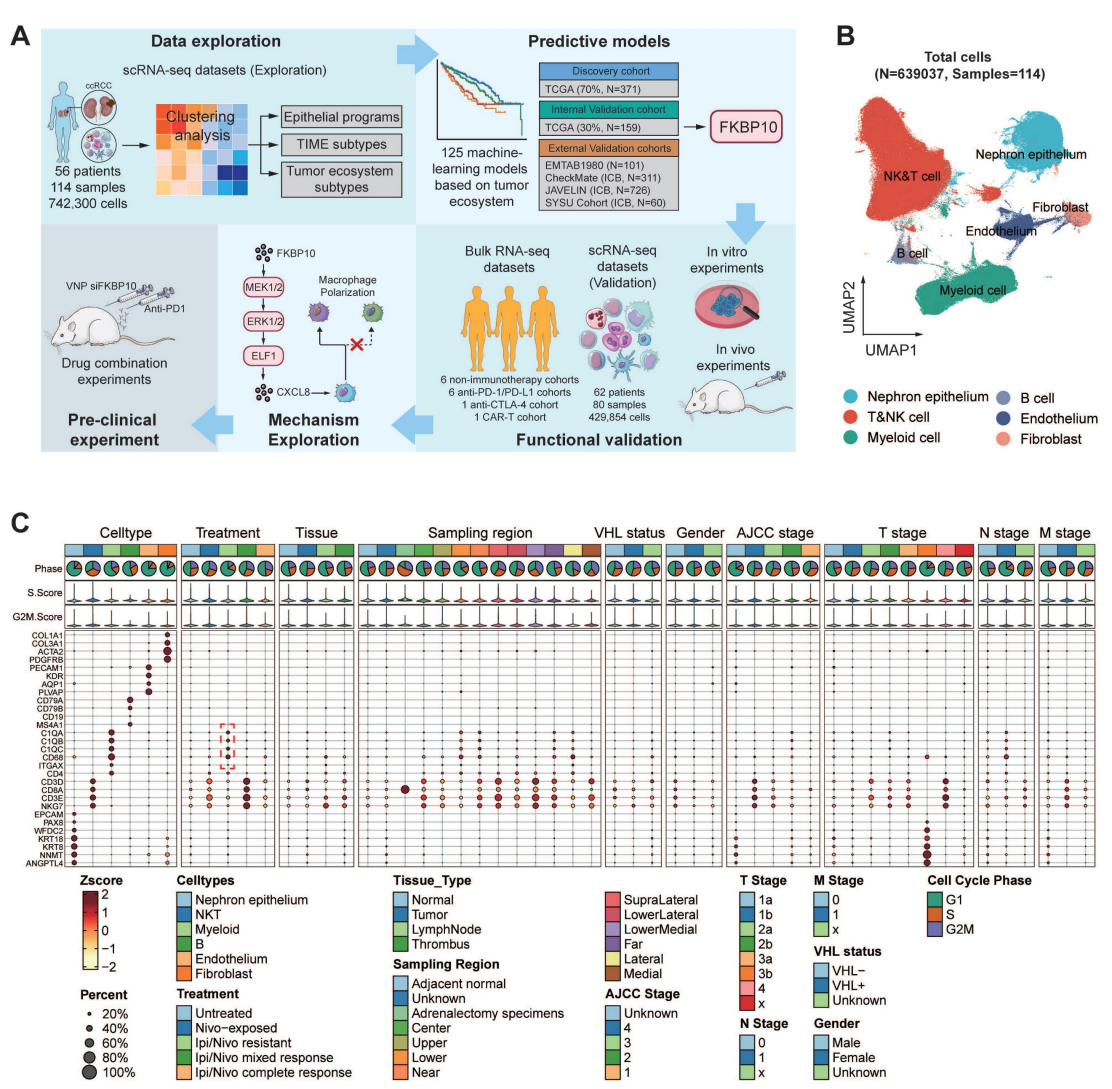
** Single-cell transcriptional landscape of the ccRCC ecosystem.** (A) Scheme of the overall study design. TIME, tumor immune microenvironment; ICB, immune checkpoint blockade; IHC, immunohistochemistry. (B) UMAP plot showing six major cell types. Dots represent individual cells, and colors represent different cell populations. T&NK, T lymphoid and natural killer cells. (C) Dot plot showing the marker genes for each major cell population and several clinical characteristics. Treatment refers to any therapeutic interventions administered to the samples prior to single-cell profiling. Nivo, Nivolumab (anti-PD-1 monotherapy); Ipi, Ipilimumab (anti-CTLA-4 monotherapy). Region refers to the sampling regions of tumor tissues. Near, tumor tissue directly adjacent to normal kidney; Far, tumor tissue distal to normal kidney; Center, tumor tissue between Near and Far. VHL+/-, with/without Von Hippel-Lindau gene mutation.

We integrated and normalized seven public scRNA-seq datasets comprising 73 tumor samples, 37 adjacent normal tissues, 2 thrombi, and 2 metastatic lymph nodes from 56 individuals (**Table S4**). After stringent quality control, we obtained 639,037 cells from 742,300 cells spanning 114 human samples (**Fig. 1A; Fig. S2-3**). Louvain clustering revealed six clusters across samples spanning NK and T lymphoid, B lymphoid, myeloid, endothelial and epithelial cells, and fibroblasts (**Fig. 1B**).

We analyzed canonical marker genes for each cell type and examined the clinical characteristics (**Fig. 1C**). Notably, immunotherapy-resistant patients, especially those with lymph node metastasis, exhibited high expression of myeloid cell markers. Cell cycle analysis indicated that the majority of these cells resided in the G0/G1 phase, indicative of relative dormancy or slow proliferation. Conversely, patients responsive to ipilimumab/nivolumab showed increased expression of CD8+ T cell markers, suggesting active immune response. In addition, our spatial analysis uncovered regional differences in immune cell distribution within the tumors, with myeloid cells enriched in the lower and lateral tumor regions, and T cells being prevalent elsewhere. These findings suggest that resistance to ICB may be linked to a higher presence of myeloid cells and a lower presence of CD8+ T cells, with distinct spatial patterns across the tumor.

### Cellular Module Analyses Reveal Four TIME Subtypes

**Figure 2:**
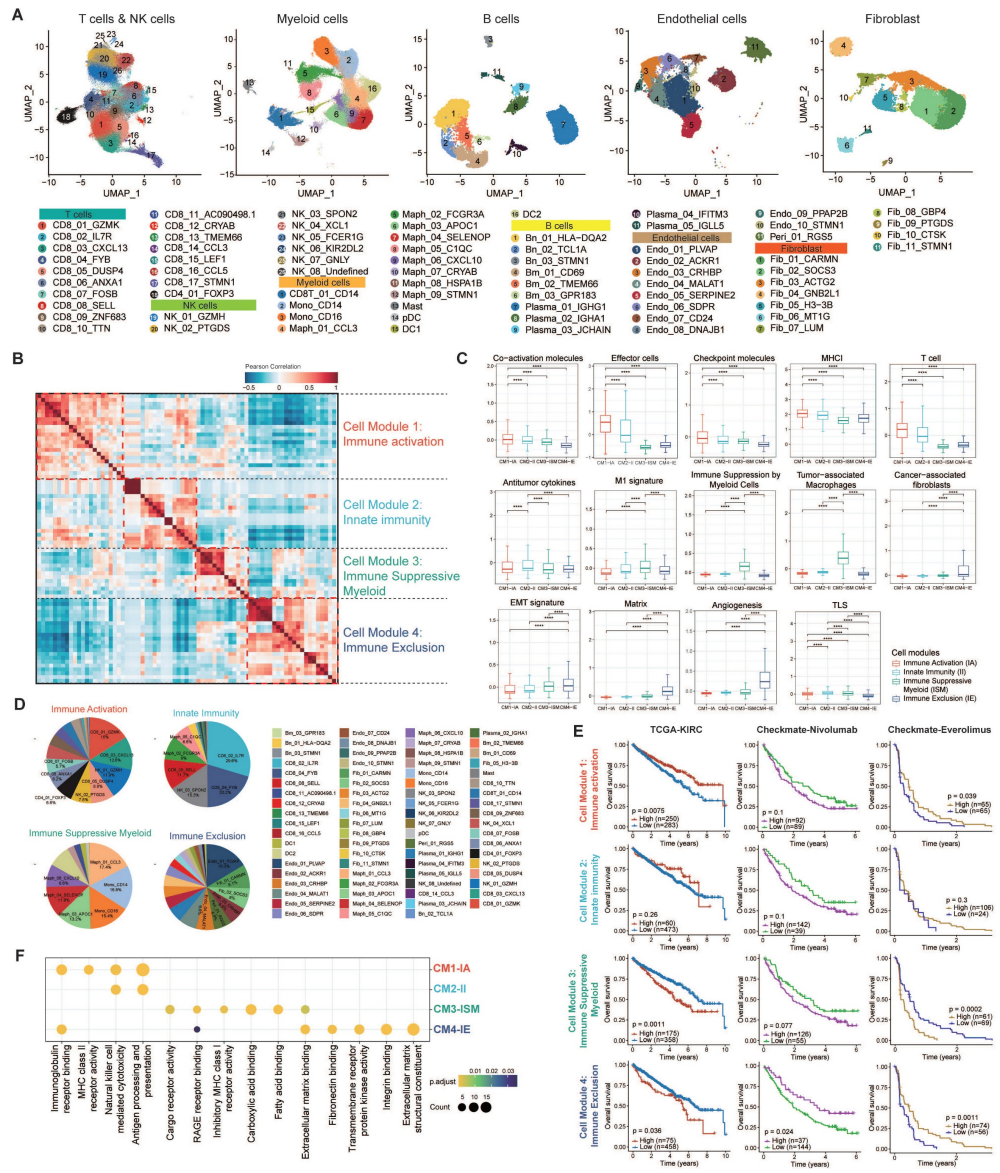
** Four distinct cellular modules of TIME in ccRCC.** (A) UMAP plots showing 75 TIME cell clusters. Cells are grouped into five panels: T cells and NK cells, myeloid cells, B cells, endothelial cells, and fibroblasts, with color coded by cluster ID. Bn, naïve B cell; Bm, memory B cell. (B) The four cellular modules based on Pearson correlations of cell clusters (shown in Fig. 2A) from samples. Each cellular module corresponds to a TIME subtype, in which the phenotype was designated based on four aspects shown in Fig. 2C-F. (C) Differential expression of immune-related signatures in four TIME subtypes. The Wilcoxon rank-sum test (two-sided) was applied for significance testing. *, *P* < 0.05; **, *P* < 0.01; ***, *P* < 0.001. IA, immune activation; II, innate immunity; ISM, immune suppressive myeloid; IE, immune exclusion. (IA, n = 28 cases, II, n = 7 cases, ISM, n = 6 cases, IE, n = 15 cases, n denotes biologically independent patients). (D) Pie charts showing the proportion of cell clusters from each cellular module, with key cell clusters annotated. (E) Prognostic relevance of four TIME subtypes. Overall survival of cases was stratified by each cellular module proportion. Log-rank test was used for statistical analysis. (F) Dot heatmap showing enriched Hallmark pathways across four TIME subtypes. P.adjust refers to the Benjamini-Hochberg-adjusted p-value.

Building on these insights into myeloid cell enrichment, we next sought to explore the broader cellular composition of TIME to understand its functional implications for therapy resistance. From a cohort of 530,848 single cells, we identified 75 distinct cell clusters (**Fig. 2A; Table S5**). Notably, tumors with high tertiary lymphoid structure (TLS) signature expression exhibited significant enrichment of antibody-secreting plasma cells (IgG, IgA, and IFITM3+ plasma cells) and GPR183+ memory B cells (**Fig. S4A-E**). These subsets expressed key germinal center B-cell markers (**Fig. S4F**), suggesting functional germinal center reactions within the TLS. This finding aligns with previous studies showing that in TLS+ RCCs, plasma cells secrete higher levels of IgG and IgA, supporting the role of TLS in anti-tumor immunity and its association with favorable immunotherapy responses 22.

**Figure 3:**
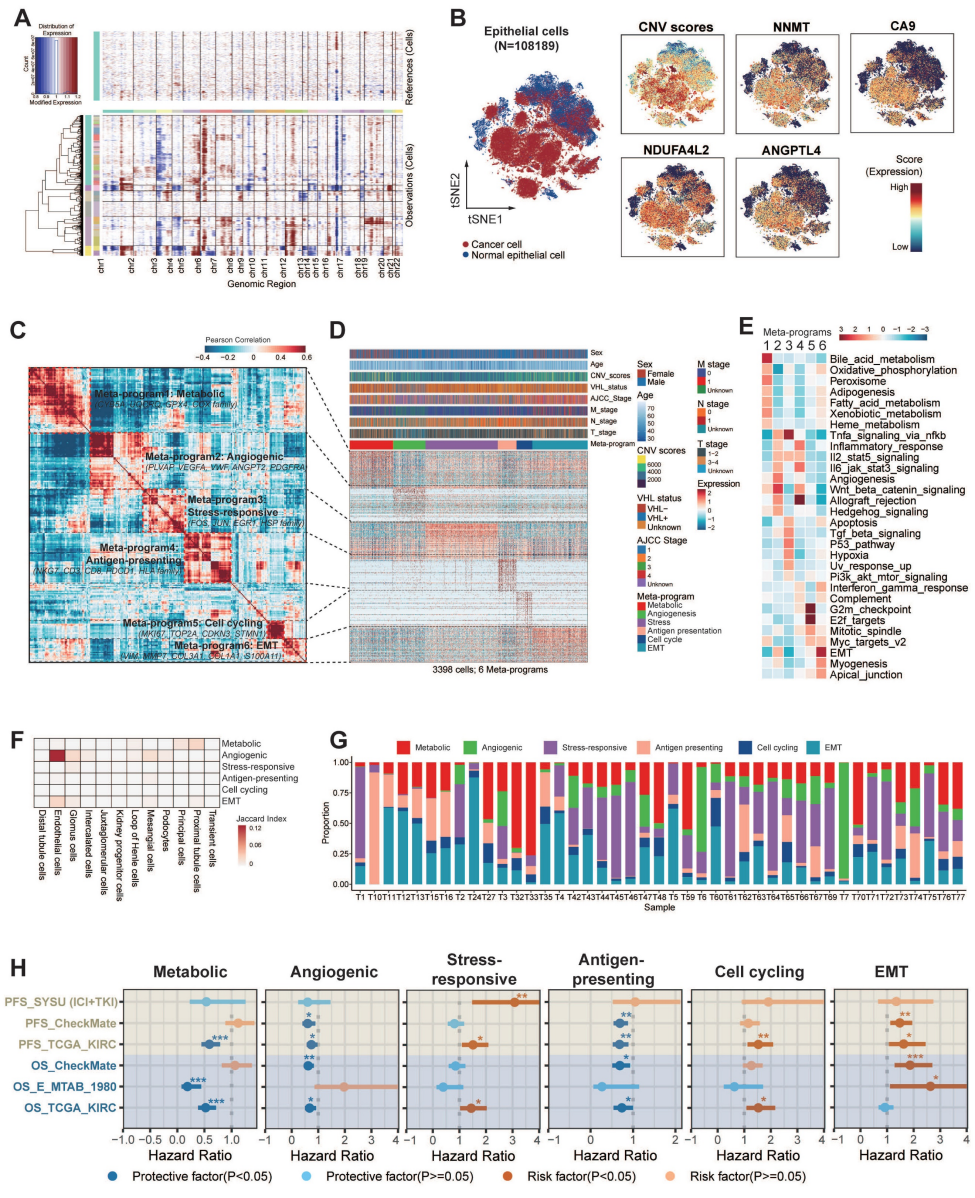
** Six common gene expression programs of epithelial cells in ccRCC tumors.** (A) Large-scale CNVs for ccRCC epithelial cells. Red regions stand for gene amplifications, while blue regions for gene deletions. The inferred CNV pattern of non-malignant epithelial cells is shown in the upper panel. Malignant cells from different patients are indicated by different color bars on the left of the heatmap. (B) t-SNE plot of all epithelial cells (n = 108,189), color-coded by cell types, CNV score, and expression levels of classic ccRCC biomarkers (NNMT, CA9, NDUFA4L2, ANGPTL4), respectively. Previously recognized markers reaffirm the accuracy of tumor cells identified by InferCNV. (C) The six malignant meta-programs (MPs) across the tumors. Each meta-program corresponds to a tumor cell state that was defined based on Fig. 3E-F. The hierarchical clustering heatmap shows the pairwise correlations of 250 gene expression programs across 43 tumors. (D) Heatmap depicting the expression of genes within each MP across single cells. Randomly selected 5% of cells (n = 3,398) for six MPs are shown, with annotated by cell state, T, N, M stage, AJCC stage, VHL status, CNV score, age, and gender. (E) Heatmap showing different pathways enriched in cells for each MP, colored by z-score normalized GSVA scores. (F) Jaccard similarities of the signatures between six MPs (y axis) and twelve renal cell types (x axis). (G) Stacked histograms showing the proportions of malignant epithelial cell states for each patient. (H) Forest plots showing overall survival in four ccRCC cohorts (TCGA-KIRC, E-MTAB-1980, CheckMate, and SYSU cohort). The high and low groups were divided by the best cutoff value of CIBERSORTx deconvoluted cell fractions corresponding to 6 MPs. *, *P* < 0.05; **, *P* < 0.01; ***, *P* < 0.001.

Hierarchical clustering was then applied to investigate the functional roles of all immune cell clusters. This helped us identify four stable cellular modules (CMs), CM1 to CM4 (**Fig. 2B**). The four CMs correspond to four TIME subtypes based on four criteria: (1) expression of TIME-related gene signatures (**Fig. 2C; Fig. S5A-B**), (2) cell cluster proportions (**Fig. 2D**), (3) prognostic and ICB responsive relevance (**Fig. 2E; Fig. S5C-D**), (4) functional signaling pathway enrichment (**Fig. 2F; Table S6**).

The CM1-IA subtype (immune activation, IA) exhibited prominent immune activation signatures encompassing co-stimulatory molecules, effector cells, checkpoint markers, and MHC-I/II pathways through immunoglobulin receptor binding. Dominated by effector memory (CD8+GZMK+/CXCL13+/DUSP4+), tissue-resident (CD8+ZNF683+) T cells, regulatory T cells (CD+FOXP3+), and cytotoxic NK subsets (GZMH+/PTGDS+), CM1-IA demonstrated prognostic significance with improved overall survival (OS) in TCGA cohort and enhanced mTOR inhibitor (everolimus) response efficacy.

The CM2-II subtype (innate immunity, II) displayed distinct anti-tumor cytokine signatures with enrichment of NK cells, CD16+ macrophages, and naïve CD8+ T cells (SELL+/IL7R+). Its association with immunological regulatory pathways, including TNF/NF-κB signaling pathways underscored its significance. BisqueRNA deconvolution revealed no significant survival variations in CM2-II cell populations.

The CM3-ISM subtype (immunosuppressive myeloid, ISM) demonstrated elevated myeloid-mediated immune suppression and tumor-associated macrophage signatures. Pathway enrichment analysis pointed to NOD-like receptor signaling pathway, which was reported to aggregate MDSCs and induce the M2 macrophage polarization, thereby forming an immunosuppressive microenvironment 58. CM3-ISM was notably enriched in ipilimumab/nivolumab-resistant patients. Elevated ISM abundance consistently predicted poor prognosis in both TCGA and everolimus-treated cohorts, and correlated with compromised anti-PD-1 response.

The CM4-IE subtype (immune exclusion, IE) exhibited robust expression signatures related to cancer-associated fibroblasts (CAFs), EMT, extracellular matrix remodeling, and angiogenesis. This fibroblast- and endothelial cell-enriched subtype showed activation of stromal reprogramming pathways. While CM4-IE enrichment correlated with poor prognosis in TCGA cohort, it paradoxically associated with improved outcomes in the treated patients (CheckMate). This improved response was linked to a distinct TME characterized by spatial co-localization of CM4-IE-high cells with CD8+ T cells and an enrichment of H3-3B+ fibroblasts, as revealed by our spatial transcriptomic analysis (**Fig. S4G-H**).

### Inter-tumor Heterogeneity of TIME Subtypes

Following the classification of TIME subtypes, we extended our investigation to examine the heterogeneity of TIME across 52 ccRCC patients (**Table S7**). Different tumor statuses and pathological grades exhibited obvious TIME preference, indicating an association with aetiologies and tumor progression (**Fig. S5E**). Analyses of cellular composition demonstrated a striking divergence in immune architecture: 30.8% (16/52) of cases exhibited monotypic TIME dominance (≥ 65% single-subtype infiltration), while 69.2% (36/52) displayed heterotypic TIME integration (**Fig. S5F**). This binary stratification uncovered significant clinical associations: the monotypic subgroup showed a higher prevalence of advanced-stage disease (Stage III/IV, *P* = 0.034) (**Fig. S5G**).

Our analysis of TIME composition reveals significant heterogeneity in ccRCC, with monotypic infiltration being linked to more aggressive disease. These findings highlight the importance of TIME patterns in tumor progression.

### Classification of Six Malignant Epithelial States

After investigating TIME heterogeneity, we next focused on malignant epithelial cell diversity in ccRCC. We identified malignant epithelial cells in ccRCC using copy number variant (CNV) score (**Fig. 3A**). CNV analysis revealed that most of these epithelial cells were tumor cells, showing high expression of ccRCC marker genes, including NNMT, CA9, NDUFA4L2, and ANGPTL4 17 (**Fig. 3B**). We then analyzed the transcriptional profiles of these cells to explore intra-tumor heterogeneity. Using cNMF, we clustered epithelial cells within each sample, resulting in 250 distinct subclusters across 114 ccRCC tumors. The heatmaps show the top 30 genes for each subcluster (**Fig. S6-16**). To further refine our analysis, we grouped these subclusters into six core meta-programs (MPs, MP1 to MP6; **Table S8**). Cells expressing ≥ 70% of program genes were defined as programmed cells (**Fig. 3C-D**). We further explored the most activated pathways in the programmed cells to define their specific states (**Fig. 3E; Fig. S17; Fig. S18A**).

Our analysis revealed that MP1-Metabolic was primarily characterized by gene sets associated with metabolism (e.g., CYB5A, GPX4, UQCRQ). Pathway analyses further demonstrated the activation of metabolic pathways, including oxidative phosphorylation, fatty acid metabolism, xenobiotic metabolism, and heme metabolism.

MP2-Angiogenic was enriched in genes associated with angiogenesis (e.g., PLVAP, VEGFA, ANGPT2) and exhibited upregulation of key signaling pathways such as angiogenesis, Wnt/β-catenin signaling, TNF-α/NF-κB signaling, IL-2/STAT5 signaling, and the IL-6/JAK/STAT3 signaling pathway.

MP3-Stress-responsive encapsulated immediate early genes (e.g., EGR1, JUN, FOS) that respond to cellular stimuli, with a notable upregulation of TNF-α signaling, UV response, p53, and apoptosis pathways.

MP4-Antigen-presenting was distinguished by increased expression of MHC-II molecules (e.g., CD3, PDCD1, HLA family), integral to the initiation of adaptive antitumor immune responses. The activation of immunity-related pathways, including allograft rejection, IFN-γ, IFN-α response, and the complement pathway, potentially indicated reactivity to tumor neoantigens.

MP5-Cell cycling was characterized by high expression of genes involved in cell proliferation (e.g., MKI67, TOP2A, STMN1), suggesting active tumor cell proliferation through the activation of E2F targets, G2M checkpoint, and MYC target pathways.

Finally, MP6-EMT was marked by the expression of stress keratins (e.g., VIM, COL3A1, COL1A1, KRT6), which are associated with keratinocyte hyperproliferation and could potentially enhance tumorigenesis and tumor growth.

### Differentiation Features of Six Malignant Epithelial States

To elucidate the differentiation characteristics of six malignant epithelial cell states in ccRCC, we integrated transcriptomic similarity analysis with stemness quantification. MP1-Metabolic exhibited striking molecular similarity with proximal tubular epithelial cells, preserving most of renal developmental transcription factors including HNF4A and PPARA. This molecular continuity supports the theory that ccRCC originates from tubular epithelial progenitors 16. MP2-Angiogenic demonstrated an unexpected transcriptional overlap with endothelial cells, particularly in VEGF signaling components (FLT1, KDR) and extracellular matrix remodeling enzymes (**Fig. 3F**).

CytoTRACE analysis revealed a stemness gradient across malignant cell states (**Fig. S18B**). MP1-Metabolic and MP2-Angiogenic exhibited the lowest stemness indices, indicating terminal differentiation states with low differentiation potential. MP6-EMT cells had significantly higher stemness scores than the other subtypes, indicating higher differentiation potential (*P* < 0.001). Notably, MP6-EMT dominated the tumor ecosystem, constituting most of total malignant cells across samples (**Fig. S18B** right panel). This stemness hierarchy showed striking clinical relevance: advanced-stage patients (T3-T4/N1/M1) harbored a higher proportion of MP6-EMT cells, and MP6-EMT enrichment conferred a higher risk of CTLA-4/PD-1 treatment failure (*P* < 0.05) (**Fig. S18C**). Taken together, the analysis suggests that the MP6-EMT tumor cells may have stronger self-renewal ability and therapeutic resistance potential.

These results revealed that the malignant cell states of ccRCC showed different differentiation characteristics. MP1-Metabolic/MP2-Angiogenic represented terminal differentiation states, characterized by high transcriptional concordance and low stemness. In contrast, MP6-EMT occupies a more primitive position, marked by stem-like features and transcriptional divergence. This systematic characterization delineates malignant plasticity along differentiation hierarchies and establishes prognostic correlations. It provides a conceptual framework for mapping tumor evolutionary trajectories to clinical outcomes.

### Clinical Association of Six Malignant Epithelial States

Our analysis revealed the clinical relevance of six heterogeneous malignant cell states (**Fig. 3G**). Further analysis revealed that tumor ecosystems exhibited state dominance patterns, with 68% of patients showing >50% prevalence of a specific state (**Fig. 3G, Fig. S18D**), establishing cell state composition as a novel stratification biomarker. Thus, we now aim to explore their stratification capacity for prognosis. Validation through CIBERSORTx deconvolution across four independent cohorts (TCGA-KIRC [n = 537], E-MTAB-1980 [n = 101], CheckMate [n = 121], SYSU-ICI+TKI [n = 60]) confirmed its prognostic stratification capacity (**Fig. 3H**). Samples were stratified into high and low groups based on optimal cutoff points for cell proportions corresponding to six MPs. Survival analysis revealed significant associations between elevated proportions of cycling, stress-responsive, and EMT states and poor prognosis, while increased proportions of metabolic, angiogenic, and antigen-presenting states were associated with a more favorable prognosis. Although certain cohorts showed limited significance (HRs 0.9-1.1), consistent trend directions suggested biological relevance beyond statistical power constraints.

Our analysis presents a high-resolution atlas of malignant epithelial diversity in ccRCC, categorizing tumor cells into six distinct transcriptional MPs. These MPs not only represent unique functional and differentiation states but also demonstrate significant prognostic relevance, offering valuable insights into tumor heterogeneity and potential therapeutic strategies.

### Detection of Four Tumor Ecosystem Subtypes

**Figure 4:**
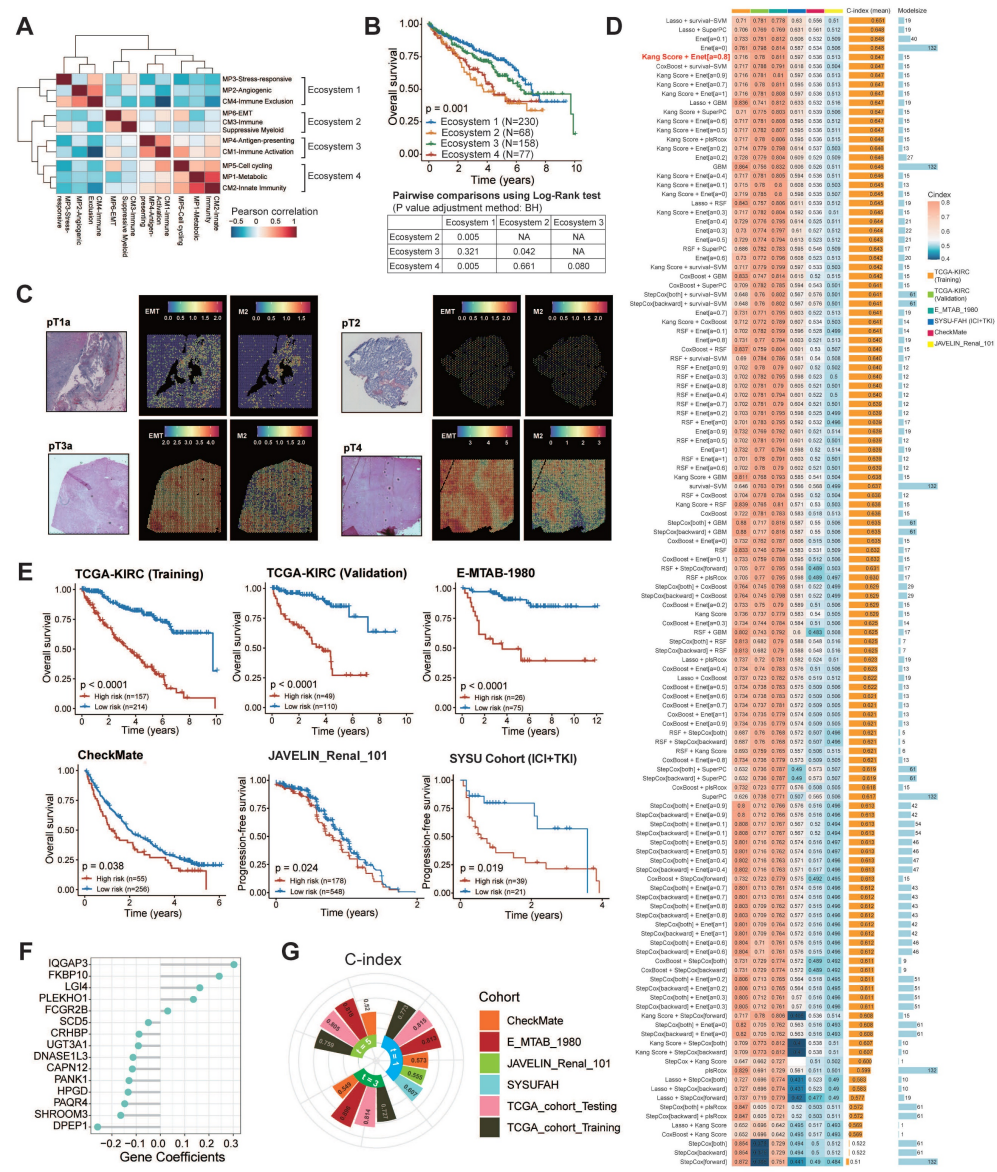
** Four ecosystem subtypes in ccRCC and construction of 126 machine learning-based models.** (A) Heatmap showing pairwise correlations of four cellular modules and six malignant meta-programs. Clustering identified four ecosystems across 43 tumors. (B) Kaplan-Meier survival curves showing overall survival of each TCGA-KIRC patient assigned to a single ecosystem. The table below shows the results of the pairwise tests between any two ecosystems. BH, Benjamini-Hochberg adjustment. (C) Spatial transcriptomic analysis shows that EMT-high tumor cells co-localize with M2 macrophages and tend to be located at the edge of tumor tissue. As the tumor stage progresses, the enrichment of EMT-high tumor cells and M2 macrophages becomes more pronounced. (D) Heatmap showing a total of 126 prediction models annotated with C-index. Histogram on the right shows the average C-index across five validating cohorts and the model size. Model size refers to the number of genes included in the models. The optimal model is marked in red. (E) Kaplan-Meier survival curves of overall survival according to the ISM-EMT-RS in TCGA-KIRC (n = 371), E-MTAB-1980 (n = 530), CheckMate cohorts (n = 311), and progression-free survival in JAVELIN-Renal-101 (n = 726), SYSU cohort (n = 60). Log-rank test was used. (F) 1-, 3-, and 5-year time-dependent C-index of ISM-EMT-RS across all datasets. (G) Coefficients of 15 mRNAs in the optimal model.

Building on our prior characterization of immune and tumor cell heterogeneity in ccRCC, we now integrate these components to map the complete tumor ecosystem. Through hierarchical clustering of six malignant cell states and four TIME subtypes, we identified four distinct ecosystems with functional interplay patterns (**Fig. 4A**).

To delineate ecosystem-specific communication patterns, we mapped ligand-receptor networks across the subtypes. Ecosystem 1 couples angiogenic/stress-responsive tumor cells with CM4-IE subtypes, revealing vascular niche co-evolution. Ecosystem 2 features aggressive EMT tumor cells synergizing with CM3-ISM subtypes, suggesting myeloid-mediated immune evasion. Ecosystem 3 combines antigen-presenting tumor cells with CM1-IA subtypes. In this ecosystem, tumor cells engage with cytotoxic effectors (NKT_01_GNLY, CD8_16_CCL3), potentially enabling localized immune activation. Ecosystem 4 aligns metabolic/cycling tumor cells with CM2-II subtypes, indicating proliferative-metabolic adaptation. Tumor cells in ecosystem 4 are connected to NK and naïve T cells (CD8_02_IL7R) (**Fig. S19**). These distinct cellular interactions are fundamental to the organization of the ecosystem subtypes.

Transcriptomic profiling revealed ecosystem-specific chemokine/cytokine networks that coordinate tumor-TIME crosstalk (**Fig. S20A-B**). In ecosystem 1, angiogenic/stress-responsive tumor cells interacted with endothelial cells through angiogenesis signaling molecules, such as VEGF-VEGFRs and CADM1-NECTIN3. Notably, in ecosystem 2, most chemokines tightly bind to DPP4, an enzyme known to negatively regulate lymphocyte trafficking, inhibit T cell migration, and impair tumor immunity by preserving the functional chemokine CXCL10 59. Ecosystems 3 and 4 demonstrated coordinated chemokine expression patterns between tumor and TIME cells, suggesting autocrine reinforcement loops (e.g., CXCL16-CXCR6/CXCL2-CXCR2 in ecosystem 3 and CXCL2/3/8-CXCR1/2 in ecosystem 4). These axis-specific signaling architectures underpin the functional specialization of the ecosystems.

At the clinical level, we constructed four ecosystem-specific signatures based on the expression profiles of TIME subtypes, MPs, and specific ligand-receptor pairs (**Table S9**). Stratification of the TCGA, E-MTAB-1980, ICGC, CheckMate, JAVELIN and SYSUFAH cohorts revealed consistent group distributions, indicating the robustness of this categorization (**Fig. S20C**). Stratification of the TCGA cohort using ssGSEA identified four distinct prognostic groups. Kaplan-Meier analysis revealed significant survival disparities (**Fig. 4B**), with ecosystem 2 demonstrating the worst clinical outcomes (log-rank *P* < 0.001), while ecosystems 1 and 3 exhibited a favorable prognosis. These findings collectively establish the clinical relevance of tumor ecosystem classification. The ecosystem-specific GSEA analysis revealed distinct enrichment profiles (**Fig. S20D; Fig. S21**). The ecosystem 1 signature was significantly and positively enriched in the angiogenesis phenotype. The ecosystem 2 signature showed significant positive enrichment in the Toll-like receptor and NOD-like receptor signaling pathways, indicating its suppression. The ecosystem 3 signature was positively enriched in antigen presentation and adaptive immune response, and the ecosystem 4 signature exhibited significant positive enrichment in the reactive oxygen species pathway. GSEA using our custom immune gene sets successfully identified distinct biological states, offering mechanistic insights into their clinical relevance.

At the spatial transcriptomic level, EMT-high tumor cells co-localized with M2 macrophages and CD8+ T cells, with a notable tendency to cluster at the edge of the tumor tissue in ecosystem 2. As the tumor stage progresses, the enrichment of EMT-high tumor cells and M2 macrophages becomes more pronounced (**Fig. 4C; Fig. S22**). Mechanistically, NicheNet analysis identified ecosystem 2-specific upregulation of EMT-inducing ligands (TGF-β1, IL-1β, OSM; **Fig. S23A**), indicating stromal-mediated EMT activation through IL-1β/TGF-β1 signaling. Metascape pathway enrichment analysis further highlighted the involvement of pathways related to cell migration, cytokine response, EMT, and immunosuppression (**Fig. S23B-D**). Together, our analyses suggest that EMT-high tumor cells interact with immune cells, particularly M2 macrophages, through cytokine signaling, which contributes to tumor progression and helps explain the poor survival outcomes observed in ecosystem 2 patients.

In summary, our analysis characterizes four distinct ccRCC tumor ecosystems, each defined by specific interactions between tumor cells and the TIME. These ecosystems exhibit unique activation patterns of signaling pathways that influence tumor behavior and immune dynamics. Notably, ecosystem 2 is associated with poor prognosis, underscoring its role in immune evasion.

### Construction and Validation of 126 Machine Learning Models

Our previous findings highlight the clinical relevance of tumor ecosystem classification as a predictive tool for patient stratification and potential therapeutic targeting. Given that samples from ecosystem 2 exhibited the worst survival outcomes, we established ISM-EMT-Sig using machine-learning framework described in method sections. We tested 126 different prediction models on the TCGA-KIRC dataset, calculating the C-index for each model across various validation datasets (**Fig. 4D**). The HRs and their confidence intervals for 126 prognostic models across training set, internal validation set, and four distinct external validation sets were presented in the forest plot (**Fig. S24A**). The optimal model was a combination of Kang's Model and Elastic Net (Enet) (α = 0.8) with 15 genes, yielding the highest average C-index (0.647), which showed a substantial C-index across all validation datasets. Ten-fold cross-validation was used to assess the robustness and generalizability of the optimal model (**Fig. S24B-C**). The optimal model demonstrated robust predictive performance, as evidenced by a mean coefficient of determination (R^2^) of 0.847.

Risk stratification using these weighted transcripts (**Table S10**) consistently discriminated high-risk patients with worse OS/PFS across five cohorts (log-rank *P* < 0.05), including ICB-treated populations (CheckMate, JAVALIN_Renal_101, SYSU cohort) (**Fig. 4E-F**). Multivariate Cox regression demonstrated that ISM-EMT risk score (ISM-EMT-RS) remained statistically significant (all *P* < 0.05) after adjusting for available clinical factors, such as age; gender; disease stage; PD-L1 status (**Fig. S25**). This confirmed the ISM-EMT-RS as an independent OS predictor (TCGA-KIRC: HR = 2.06, 95% CI 1.60-2.65, *P* < 0.001; E-MTAB-1980: HR = 2.08, 95% CI 1.08-3.98, *P* < 0.001; CheckMate: HR = 1.13, 95% CI 0.96-1.34, *P* = 0.137). For PFS, the ISM-EMT-RS showed some predictive value but did not reach statistical significance.

### Evaluation of the Optimal Machine Learning Model

Receiver operating characteristic (ROC) analysis measured the discrimination of ISM-EMT-RS, with 1-, 3-, and 5-year AUCs of 0.815, 0.814, and 0.805 in TCGA-KIRC; 0.813, 0.896, and 0.818 in E-MTAB-1980; 0.573, 0.549, and 0.520 in CheckMate, respectively. 1-year AUCs of SYSU cohort and JAVELIN cohort are 0.607 and 0.555, respectively (**Fig. 4G**). Furthermore, we compared the performance of ISM-EMT-RS with other clinical variables in predicting prognosis. As depicted in **Fig. S26**, ISM-EMT-RS exhibited distinctly superior accuracy compared to other variables, including age, gender, pathological grade, T, N, M, and AJCC stage (except for the comparison between ISM-EMT-RS and AJCC stage in the TCGA-KIRC-training and TCGA-KIRC-total cohort). These findings collectively suggest that the ISM-EMT-RS has stable and robust prognostic performance in multiple independent cohorts. AJCC stage is a commonly used prognostic tool for the clinical management of ccRCC, and multivariate Cox regression analysis demonstrated that AJCC stage was statistically significant across multiple cohorts. Thus, combining the ISM-EMT-RS with AJCC stage may further improve the predictive ability of our model.

In summary, we established the ISM-EMT-RS as a robust, independent prognostic tool for ccRCC, with superior predictive power across multiple cohorts. The optimal machine learning model, combining Kang's Model with ENet, provides reliable risk stratification for predicting overall survival.

### Expressive Validation of Prognostic Biomarkers at mRNA and Protein Resolution

**Figure 5:**
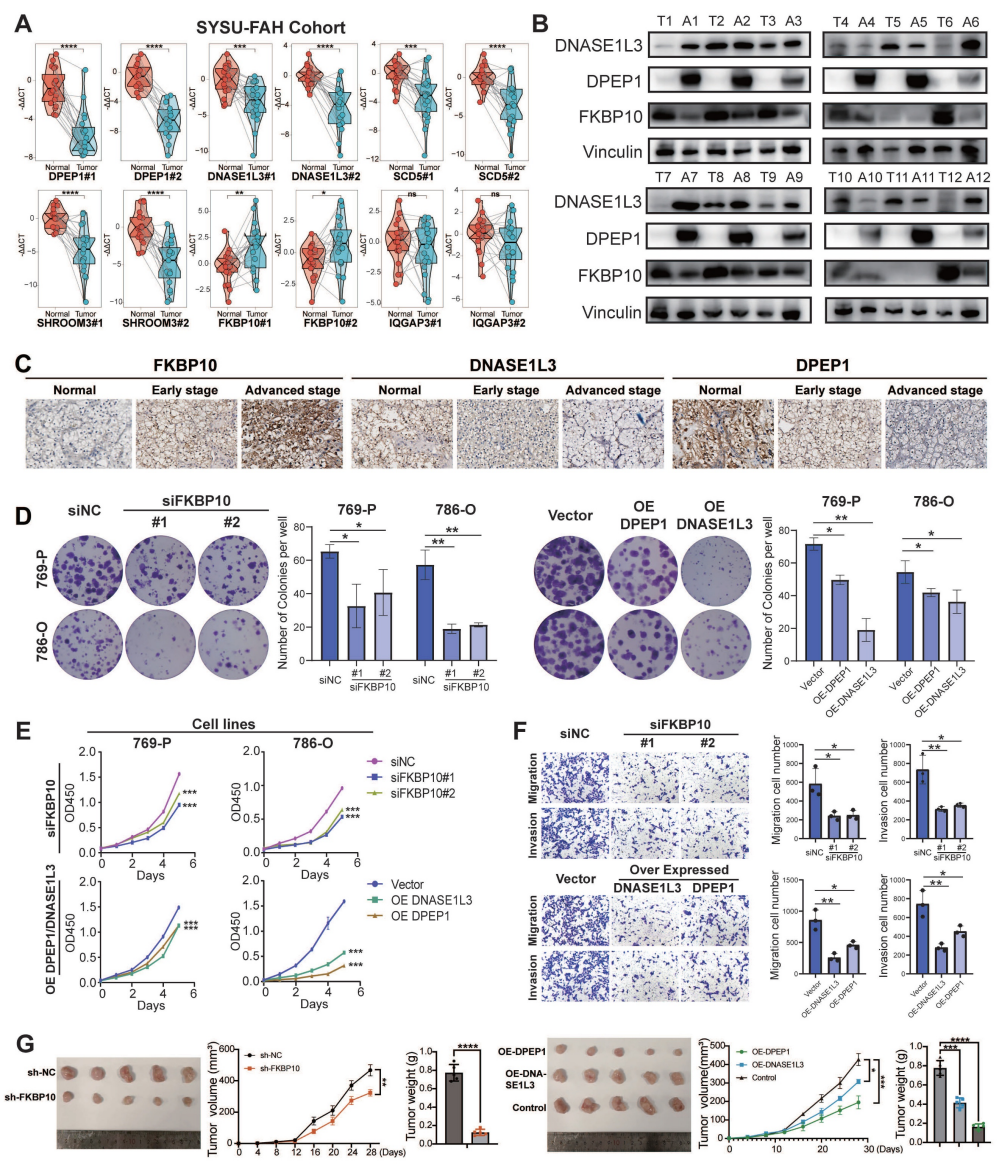
** Expression and functional analysis of FKBP10, DNASE1L3, and DPEP1 in ccRCC.** (A) mRNA expression levels of six candidate genes (FKBP10, IQGAP3, DNASE1L3, SCD5, SHROOM3, DPEP1) in ccRCC tumors versus paired normal adjacent tissues (n = 20 matched pairs). Data were quantified by qRT-PCR and analyzed using two-tailed paired Student's t-tests. (B) Representative western blot analyses of FKBP10, DNASE1L3 and DPEP1 protein expression in tumor/normal tissue pairs (n = 12 matched pairs). (C) Immunohistochemical staining of FKBP10, DNASE1L3 and DPEP1 across ccRCC clinical stages. (D, E) Colony formation (D) and CCK-8 proliferation assays (E) in 786-O and 769-P cells following FKBP10 knockdown (siFKBP10) or overexpression (OE) of DNASE1L3/DPEP1 (n = 3 independent experiments, mean ± SD). (F) Transwell migration (upper chamber without Matrigel) and invasion (with Matrigel coating) assays under indicated treatments (n = 3 independent experiments). (G) Subcutaneous xenograft growth curves (left) and final tumor weights (right) in mice (n = 5 per group) injected with: 1) Vector control, 2) FKBP10-knockdown, 3) DNASE1L3-overexpressing, or 4) DPEP1-overexpressing 786-O cells. Data were analyzed by mixed-effects model (growth curves) and two-tailed unpaired Student's t-tests (tumor weights). Data are presented as mean ± SD. *, *P* < 0.05; **, *P* < 0.01; ***, *P* < 0.001.

We selected six genes from ISM-EMT-Sig for downstream experimental validation. Specifically, we chose two genes with the highest positive coefficients and two with the highest negative coefficients, as these are likely to have the most significant impact on our model. Additionally, we included two genes with median coefficients to represent a range of moderate effects. qPCR revealed significant upregulation of FKBP10 and IQGAP3, and downregulation of DNASE1L3, SHROOM3, and DPEP1 in ccRCC tumors (*P* < 0.05) (**Fig. 5A**). Then the gene with the most significant differential expression within each of the three groups was selected for further protein expression analysis. WB results showed that FKBP10 was upregulated, while DNASE1L3 and DPEP1 were downregulated in ccRCC tissues (**Fig. 5B**).

IHC staining showed distinct expression patterns of DNASE1L3, DPEP1, and FKBP10 across normal, early, and advanced stages. DNASE1L3 and DPEP1 decreased progressively, while FKBP10 was elevated mainly in advanced stages (**Fig. 5C**).

### Functional Validation of FKBP10/DNASE1L3/DPEP1

To better understand the roles of FKBP10, DNASE1L3, and DPEP1 in RCC progression, we conducted a series of functional assays. Colony formation and CCK8 assays demonstrated that silencing FKBP10 and overexpressing DNASE1L3/DPEP1 significantly suppressed the proliferation of 786-O and 769-P cells (all *P* < 0.05) (**Fig. 5D-E**). Quantitative Transwell analysis demonstrated FKBP10 silencing reduced invasion by 54% (*P* < 0.05) and migration by 58% (*P* < 0.05), while DNASE1L3/DPEP1 overexpression attenuated invasion by 62%/39% and migration by 70%/46% compared to controls (*P* < 0.05) (**Fig. 5F**).

*In vivo* validation through subcutaneous xenograft models revealed phenotype concordance: DNASE1L3/DPEP1-overexpressing tumors showed a 49%/76% volume reduction (*P* < 0.001), while FKBP10-silenced models exhibited an 81% growth inhibition (*P* < 0.001) at endpoint compared to controls (**Fig. 5G**). These findings support FKBP10 as a metastasis driver and DNASE1L3/DPEP1 as tumor suppressors in the pathogenesis of ccRCC.

### FKBP10 as a Biomarker in Immunotherapy

Cox regression analysis across TCGA-KIRC, GSE167573, E-MTAB-1980, and ICGC-EU datasets confirmed FKBP10 as a risk factor for ccRCC, while DPEP1 and DNASE1L3 exhibited protective roles. GSEA of hallmark pathways identified EMT, KRAS signaling, and glycolysis as top enriched pathways in FKBP10-high tumors (**Fig. S27**).

**Figure 6:**
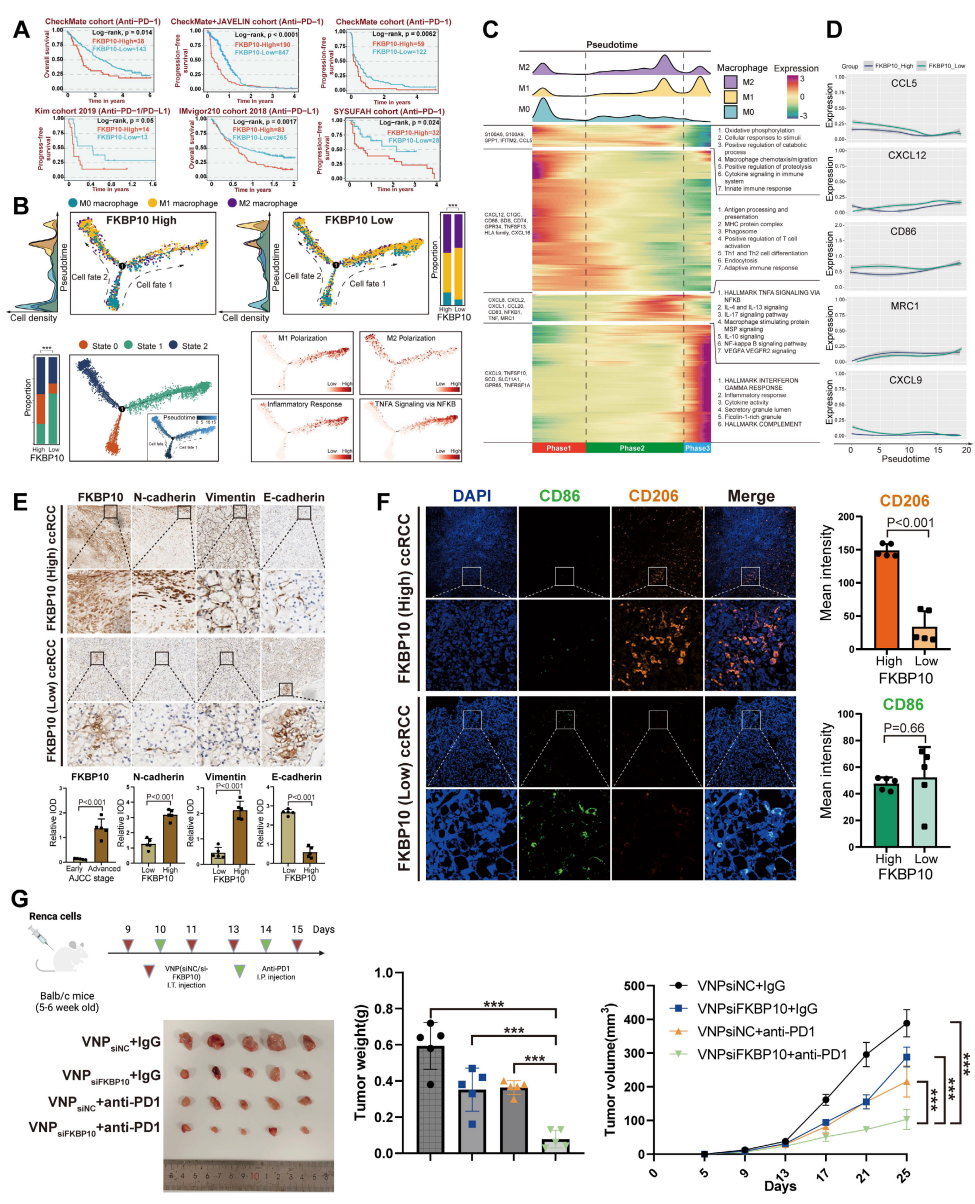
** FKBP10 shapes an immunosuppressive microenvironment and synergizes with anti-PD-1 therapy.** (A) Kaplan-Meier analysis of immunotherapy response (anti-PD-1: CheckMate cohort, n = 181; anti-PD-L1: IMvigor210, n = 348; anti-PD-1/PD-L1: Kim cohort, n = 27). Log-rank *P* values shown. (B) Monocle2-based pseudotime trajectories (n = 7,476 macrophages from 71 ccRCC samples) include: (Top) Phenotype distribution (M0/M1/M2) with cell density estimation. Stacked bars show phenotype proportions in FKBP10-high versus FKBP10-low groups (*χ²* test, ***, *P* < 0.001). (Bottom left) Two distinct differentiation states (State 1/2) identified by DDRTree dimensional reduction. Stacked bars show state proportions across groups (*χ²* test, ***, *P* < 0.001). (Bottom right) State-specific pathway activities calculated by AddModuleScore. Color scale: z-scored enrichment scores. (C) Heatmap of 1,000 differentially expressed genes (|log2FC| > 1, FDR < 0.05 by DESeq2) across pseudotime continuum (columns: 7,476 cells from 62 patients). Rows show z-score normalized expression. (Top) cell density estimation of M0/M1/M2 macrophage distribution along pseudotime. Dashed lines indicate polarization checkpoints. (Right) Top enriched pathways per gene module (FDR < 0.01). (D) Dynamic expression patterns of macrophage polarization markers along pseudotime. (E-F) Representative IHC staining (10×, 50×) of FKBP10 across AJCC stages (n = 5 per stage). Co-staining of macrophage markers CD206 (M2) and CD86 (M1) with EMT markers (N-cadherin, vimentin, and E-cadherin). (G) *In vivo* therapeutic efficacy in Renca-bearing Balb/c mice (n = 5 per group): Combination therapy (siFKBP10 + anti-PD-1) significantly reduced tumor weight and volume (***P < 0.001, two-way ANOVA) compared to monotherapies. siNC, scrambled control; i.t., intratumoral; i.p., intraperitoneal. *, *P* < 0.05; **, *P* < 0.01; ***, *P* < 0.001. All data represent mean ± SD from independent experiments unless specified. Statistical analyses performed using Benjamini-Hochberg correction for omics data.

Further survival analysis across multiple immunotherapeutic cohorts indicated FKBP10 expression as a pan-immunotherapy biomarker. Compared to DPEP1 and DNASE1L3, FKBP10 expression consistently distinguished immunotherapeutic efficacy across several cohorts, including CheckMate cohort treated with anti-PD-1 therapy (log-rank, OS: *P* = 0.014; PFS: *P* = 0.0062), CheckMate + JAVELIN cohort (log-rank, *P* < 0.0001); Kim cohort 2019 treated with anti-PD-1/PD-L1 therapy (log-rank, *P* = 0.05), IMvigor210 cohort 2018 treated with anti-PD-L1 therapy (log-rank, *P* = 0.0017), VanAllen cohort 2015 treated with anti-CTLA-4 therapy (log-rank, *P* = 0.00047), and SYSUFAH cohort treated with anti-PD-1 (log-rank, *P* = 0.024) (**Fig. 6A**, **Fig. S28**).

Multivariable Cox regression analysis revealed that high FKBP10 expression is an independent risk factor for ccRCC patients across multiple cohorts, including TCGA, E-MTAB-1980, CheckMate, JAVELIN, and SYSUFAH (**Fig. S29A**). Subgroup analysis in our multicenter SYSUFAH cohort, which received different immunotherapy regimens, further supported this finding (**Fig. S29B**). Specifically, FKBP10 was identified as a significant risk factor in the Axitinib plus Toripalimab treatment group (n = 49). In contrast, no significant association (*P* > 0.05) was observed in the Lenvatinib plus Pembrolizumab (n = 4) or Axitinib plus Pembrolizumab (n = 8) groups, likely due to the limited sample sizes.

The consistent predictive power of FKBP10 across these cohorts suggests its potential as a pan-immunotherapy biomarker. This prompted further investigation into the immunomodulatory role of FKBP10.

### FKBP10 Promotes M2 Polarization and EMT in RCC

To investigate the mechanistic role of FKBP10 in TME reprograming within ccRCC, we performed integrative scRNA-seq analyses across three independent validation cohorts (n = 429,854 cells). Notably, FKBP10_High tumors exhibited a significant depletion of total macrophage populations (*P* < 0.001, *χ²* test; **Fig. S29C**), accompanied by distinct TIME remodeling. Specifically, we observed marked reductions in anti-tumorigenic TIME subtypes and increases in the immunosuppressive CM3-ISM subtype in FKBP10_High tumors (**Fig. S29D-E**). Additionally, FKBP10 showed a significant positive correlation with the M2 polarization-promoting cytokine CXCL8 (**Fig. S29F**).

Pseudotime analysis revealed bifurcating macrophage differentiation trajectories, identifying three distinct cellular states (States 0-2). State 1 macrophages showed enrichment for M1 polarization markers (IRF5, IL12B) and pro-inflammatory TNF-α signaling pathways. State 2 cells were characterized by M2-associated markers (CD206, ARG1). FKBP10_High tumors exhibited a preferential accumulation of M2-polarized macrophages (*P* < 0.001; **Fig. 6B**).

Complementary heatmap analysis of pseudotime-dependent gene expression revealed four distinct transcriptional modules (Modules I-IV) along the macrophage differentiation continuum (**Fig. 6C**). Through kernel density estimation of M0/M1/M2 phenotypic distributions, we established three developmental phases: Phase 1 (early differentiation) predominated by M0 macrophages enriched in immune surveillance pathways (innate immune response: *P* = 4.42×10^-6^; macrophage chemotaxis/migration: *P* = 1.42×10^-3^); Phase 2 (intermediate) dominated by M2-polarized cells showing marked activation of immunosuppressive signaling (IL-4/IL-13: *P* = 2.88×10^-16^; IL-10: *P* = 3.93×10^-22^; IL-17: *P* = 6.77×10^-17^); and Phase 3 (terminal) enriched for M1-like macrophages upregulating pro-inflammatory mediators (IFN-γ response: *P* = 1.43×10^-12^; cytokine activity: *P* = 1.88×10^-5^). Pseudotime analysis demonstrated progressive activation of canonical M2 markers in FKBP10_High specimens, with MRC1 expression increasing from Phase 1 to Phase 3 (**Fig. 6D**). Conversely, FKBP10_High macrophages exhibited significant suppression of M1-associated effectors (CD86, CCL5, CXCL9, and CXCL12), indicating FKBP10's role in bidirectional regulation of macrophage polarization.

We further analyzed immune cell infiltration, stratified by FKBP10 expression levels, in the E-MTAB-1980, CheckMate, TCGA-KIRC, and JAVELIN cohorts using multiple algorithms (**Fig. S30-31**). The results showed that high FKBP10 expression was associated with a significant decrease in T cells. In contrast, there was an increase in macrophages, particularly M2 macrophages, and CAFs. ESTIMATE analysis revealed that the high FKBP10 group exhibited a significantly decreased immune score, and an increased stromal score. In summary, bioinformatic analysis suggests that FKBP10 contributes to an immunosuppressive microenvironment through macrophage reprogramming and matrix remodeling.

Consistent with the pro-metastatic role of EMT, IHC profiling revealed significant co-upregulation of mesenchymal markers N-cadherin (2.51-fold increase, *P* < 0.001) and Vimentin (4.69-fold, *P* < 0.001) in FKBP10_High tumors, concomitant with a collapse in epithelial marker (E-cadherin: 0.22-fold decrease, *P* < 0.001; **Fig. 6E**). This reciprocal regulation pattern supports FKBP10 as a potent EMT inducer in ccRCC. Multispectral immunofluorescence analysis quantitatively demonstrated the spatial enrichment of CD206+ M2 macrophages in FKBP10_High tumor stroma (mean intensity: 148.92 vs 33.69 in Low, *P* < 0.001; **Fig. 6F**). Furthermore, CD206+ clusters exhibited direct spatial adjacency to EMT+ tumor cells in the previous spatial transcriptomics analysis, suggesting paracrine crosstalk between FKBP10-reprogrammed M2 macrophages and progressing tumor cells. Our findings highlight the role of FKBP10 in creating a pro-metastatic niche through macrophage polarization and EMT.

### Combination Therapy of siFKBP10 and Anti-PD1 Shows Synergistic Effects in Cancer Treatment

Based on our findings that FKBP10 promotes M2 macrophage polarization and EMT in RCC, we next investigated whether targeting FKBP10 could enhance the efficacy of ICB. To this end, we established a syngeneic Renca renal carcinoma model in BALB/c mice (n = 5 per group) to assess the therapeutic potential of FKBP10 inhibition in combination with anti-PD-1 therapy (**Fig. 6G**). On days 9, 11, and 13, the mice received intra-tumoral injections of VNP (siNC/siFKBP10). On days 14 and 15, they were administered intraperitoneal injections of anti-PD-1. Tumor weight analysis revealed a marked reduction in tumor burden in the VNPsiFKBP10 + anti-PD-1 group compared to the control groups (VNPsiNC + IgG, VNPsiFKBP10 + IgG, and VNPsiNC + anti-PD-1). Consistently, longitudinal monitoring of tumor volume demonstrated significantly slower tumor growth in the combination group, indicating enhanced antitumor efficacy. Among all treatment arms, the VNPsiFKBP10 + anti-PD-1 group exhibited the most pronounced tumor suppression, suggesting a synergistic interaction between FKBP10 silencing and PD-1 blockade. Collectively, these results demonstrate that FKBP10 inhibition sensitizes tumors to ICB, supporting the therapeutic potential of combining siFKBP10 with anti-PD-1 treatment to improve clinical outcomes in renal cell carcinoma.

### CXCL8 is the Dominant FKBP10-Regulated Chemokine

**Figure 7:**
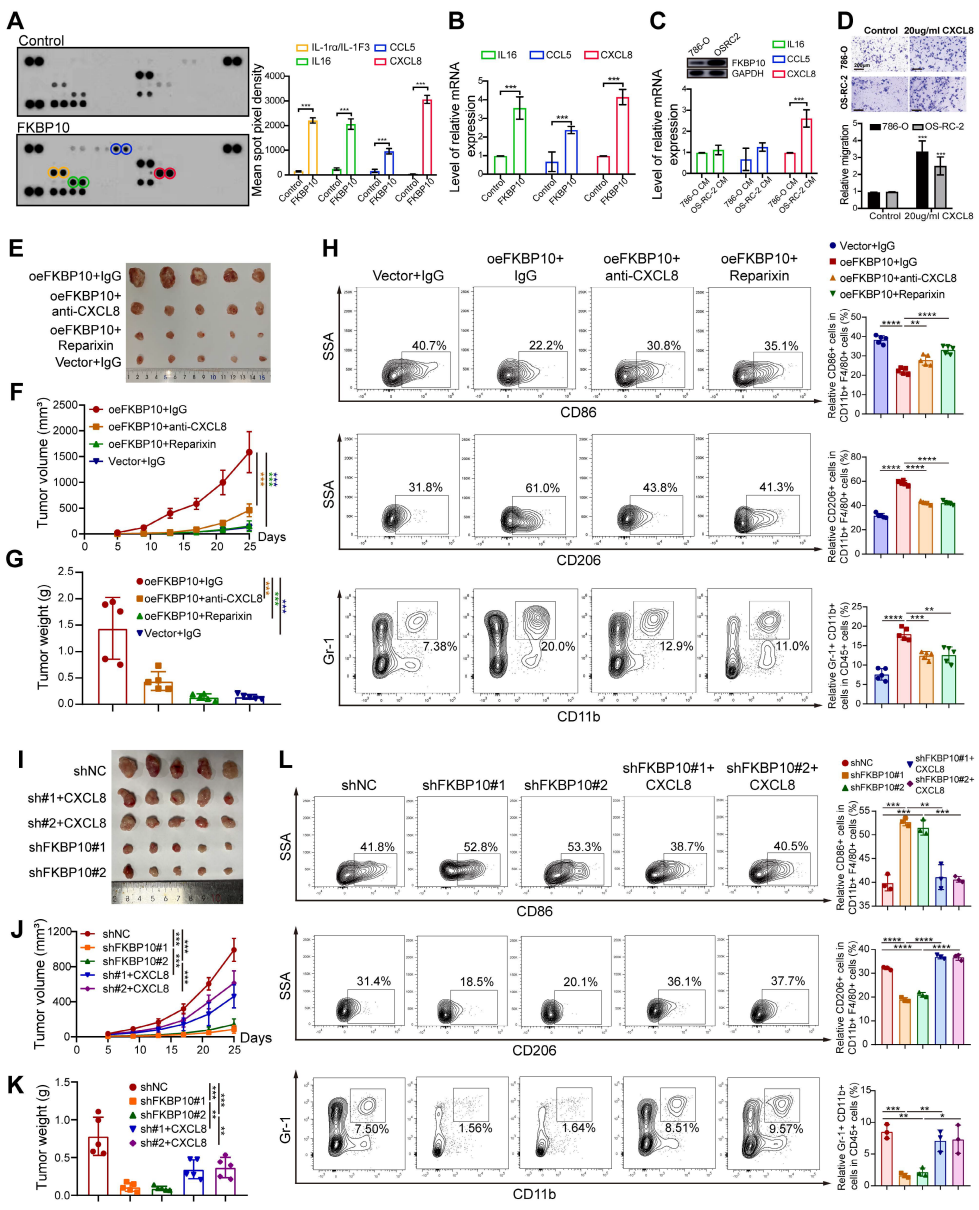
** FKBP10 modulates tumor growth and immune microenvironment through the CXCL8 signaling axis.** (A, B) Conditioned media from serum-starved 786-O cells treated with 1 μg/ml recombinant FKBP10 for 24 hours were analyzed using a human cytokine array (A). Corresponding mRNA levels of IL16, CCL5, and CXCL8 were quantified by qRT-PCR (B). (C) Western blot revealed higher endogenous FKBP10 expression in OS-RC-2 compared to 786-O cells (top). ELISA quantification showed significantly elevated CXCL8 (***, *P* < 0.001), but not IL16 or CCL5, in OS-RC-2 conditioned media (bottom). CM: conditioned media. (D) Transwell invasion assays demonstrating CXCL8-dependent macrophage-mediated invasion. Microscopic images and cumulative number of invaded RCC cells on the bottom surfaces of filters. (E-G) Representative images (E), tumor volume (F) and tumor weight (G) of excised tumors at the endpoint. Mice (n = 5 per group) were subcutaneously injected with cancer cells stably transduced with empty vector (Vector) or FKBP10-overexpressing vector (oeFKBP10). Treatments included isotype control IgG, anti-CXCL8 neutralizing antibodies, or the CXCR1/2 inhibitor Reparixin. Data are presented as mean ± SD. Statistical significance was determined by two-way ANOVA for tumor volume and one-way ANOVA with Tukey's test for tumor weight. *, *P* < 0.05; **, *P* < 0.01; ***, *P* < 0.001. (H) Flow cytometry analysis of tumor-infiltrating immune cells. Bar graphs show the proportions of M1 macrophages (CD86+), M2 macrophages (CD206+), and MDSCs (CD11b+Gr-1+). Data are mean ± SD of n = 5 biological replicates. *, *P* < 0.05; **, *P* < 0.01; ***, *P* < 0.001 (one-way ANOVA with Tukey's test). (I-K) Representative images (I), tumor growth curves (J) and tumor weight (K) of excised tumors at the endpoint. Mice (n = 5 per group) were subcutaneously injected with cancer cells stably transduced with a non-targeting control shRNA (shNC) or one of two distinct FKBP10-targeting shRNAs (shFKBP10#1, shFKBP10#2). Mice bearing shFKBP10 tumors received intratumoral injections of recombinant CXCL8. Data are mean ± SD. Statistical analysis as in E-G. (L) Flow cytometry analysis of tumor-infiltrating immune cells. Bar graphs show the proportions of M1 macrophages (CD86+), M2 macrophages (CD206+), and MDSCs (CD11b+Gr-1+). Data are mean ± SD. Statistical analysis as in H.

To explore the paracrine crosstalk between macrophages and tumor cells, we performed cytokine profiling in serum-starved RCC cells. Quantitative densitometry revealed significant upregulation of IL-1rα, IL16, CCL5 and CXCL8 compared to vehicle controls (*P* < 0.001) (**Fig. 7A**). Consistent with protein findings, qPCR demonstrated parallel mRNA induction (IL16, CCL5, CXCL8; all *P* < 0.001; **Fig. 7B**).

Baseline characterization of RCC cells revealed that 786-O cells exhibited low endogenous FKBP10 expression levels versus OS-RC-2 cells. Notably, CXCL8 secretion in OS-RC-2 conditioned media surpassed 786-O levels (*P* < 0.001), whereas IL-16 and CCL5 showed no inter-cellline disparity (*P* > 0.05) (**Fig. 7C**). These findings established CXCL8 as the dominant FKBP10-regulated chemokine in RCC.

### FKBP10 Modulates Tumor Growth and Immune Microenvironment Through CXCL8-CXCR1/2 Axis

Given that CXCL8 could induce M2 polarization 60, we established a macrophage-tumor co-culture system using Transwell assays to explore the role of CXCL8 in M2 polarization. Supplementation with CXCL8 significantly enhanced macrophage-mediated invasion, with invasion indices increasing 2-fold in OSRC2 cells and 3-fold in 786-O cells compared to baseline (**Fig. 7D**). This suggests that CXCL8 is a key factor in FKBP10-driven cancer progression.

In a subcutaneous xenograft model, overexpression of FKBP10 (oeFKBP10 + IgG) significantly promoted tumor growth, as indicated by larger tumor volumes and weights compared to the control group (Vector + IgG) (**Fig. 7E-G**). Flow cytometry of tumor tissues revealed that FKBP10 overexpression shifted the immune microenvironment toward immunosuppression, with reduced M1 macrophages and increased M2 macrophages and MDSCs. Importantly, this effect was dependent on the CXCL8-CXCR1/2 signaling pathway. Inhibition of this pathway using anti-CXCL8 neutralizing antibodies or CXCR1/2 inhibitors (Reparixin) significantly slowed tumor growth and reversed the immunosuppressive cell profile in the oeFKBP10 group (**Fig. 7H**).

Conversely, knockdown of FKBP10 (shFKBP10#1 and shFKBP10#2) significantly suppressed tumor growth compared to controls (shNC) (**Fig. 7I-K**). FKBP10 depletion resulted in a more immunostimulatory microenvironment, with increased M1 macrophages and decreased M2 macrophages and MDSCs. Importantly, exogenous CXCL8 administration reversed the effects of FKBP10 knockdown, restoring tumor growth and promoting an immunosuppressive microenvironment (**Fig. 7L**). This underscores CXCL8 as a critical mediator of FKBP10's function *in vivo*.

### FKBP10 Upregulates CXCL8 Through MEK/ERK Signaling

**Figure 8:**
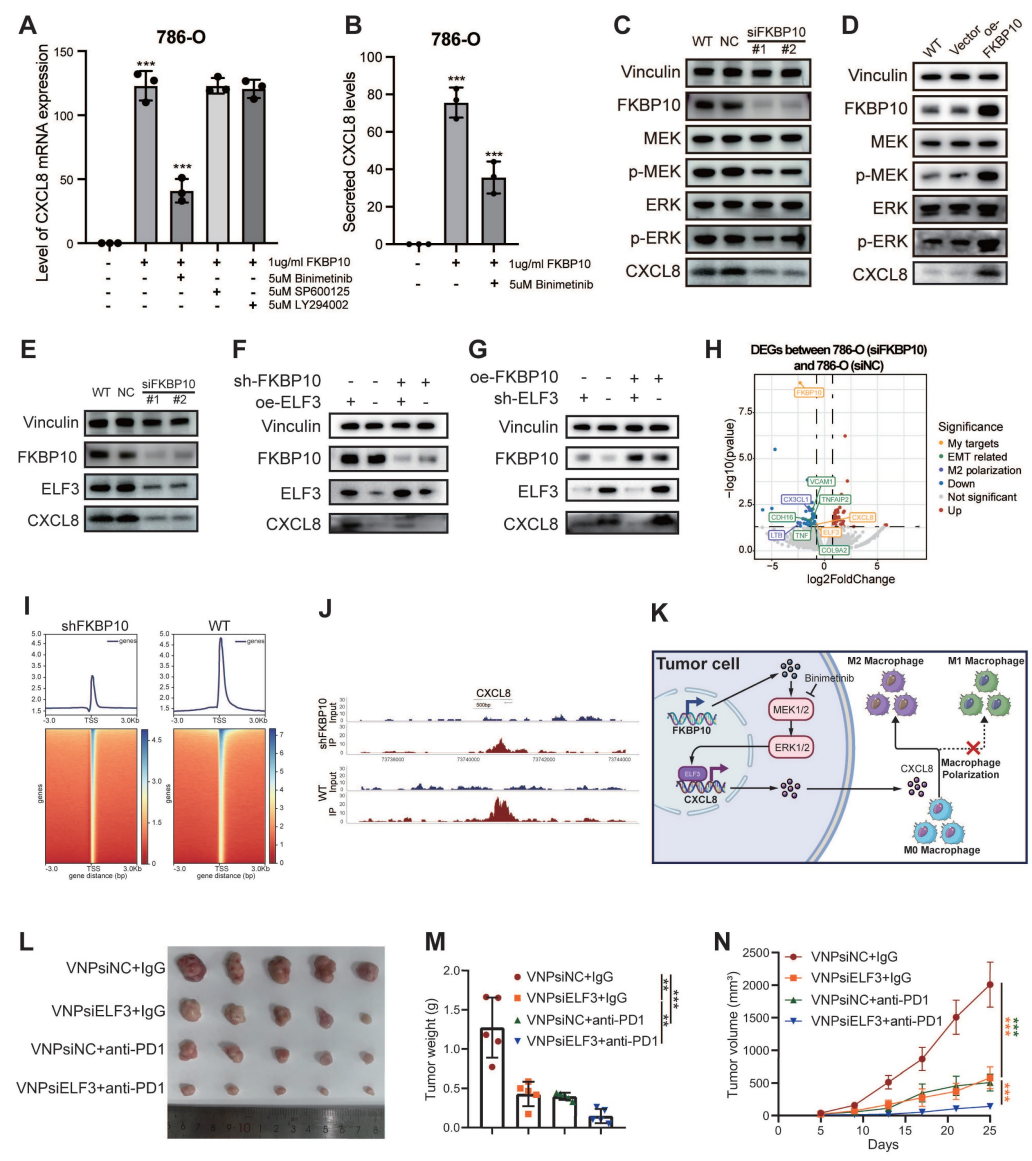
** FKBP10 upregulates CXCL8 expression via activation of the MEK/ERK/ELF3 signaling axis.** (A, B) Pretreatment of 786-O cells with Binimetinib (ERK inhibitor) prior to FKBP10 treatment significantly attenuated CXCL8 mRNA (A) and secreted protein (B) levels, as determined by qRT-PCR and ELISA, respectively. SP600125, a specific JNK inhibitor; LY294002, a specific PI-3K inhibitor. (C) Representative western blots showing MEK/ERK phosphorylation status in FKBP10-knockdown (siFKBP10), wild-type (WT), and scrambled control (siNC) 786-O cells. p-MEK, phosphorylated MEK; p-ERK, phosphorylated ERK. (D) Representative western blots showing MEK/ERK phosphorylation status in FKBP10-overexpression (oeFKBP10), wild-type (WT), and scrambled control (Vector) 786-O cells. (E) Western blot validation of ELF3 and CXCL8 downregulation in FKBP10-knockdown cells. (F) Western blot analysis showing that the downregulation of CXCL8 induced by FKBP10 knockdown (shFKBP10) was rescued by ELF3 overexpression (oeELF3). (G) The upregulation of CXCL8 induced by FKBP10 overexpression (oeFKBP10) was attenuated by ELF3 knockdown (shELF3). (H) Volcano plot of RNA-seq data (siFKBP10 versus siNC, n = 3) highlighting ELF3, CXCL8 and EMT/M2 polarization genes (|log2FC| > 1.5, FDR < 0.01). (I) Heatmap depicting the normalized ChIP-seq signal intensity of ELF3 binding peaks in regions centered on summits from shNC and shFKBP10 cells. The color scale indicates ChIP-seq enrichment, with blue representing high intensity and red representing low intensity. The experiment was performed with two biological replicates per condition. (J) Peak plots showed a reduction in ELF3 binding to the CXCL8 promoter region in shFKBP10 cells compared to WT cells. (K) Proposed mechanism of FKBP10-MEK-ERK-ELF3-CXCL8-M2 polarization axis in TME remodeling. (L-N) *In vivo* therapeutic efficacy in Renca-bearing Balb/c mice (n = 5 per group): Combination therapy (siELF3 + anti-PD-1) significantly reduced tumor volume (M) and weight (N) compared to monotherapies. siNC, non-targeting control. Data are presented as mean ± SD. Statistical significance for tumor volume was determined by two-way ANOVA; other comparisons were analyzed by one-way ANOVA. *, *P* < 0.05; **, *P* < 0.01; ***, *P* < 0.001.

To identify the signaling pathway responsible for FKBP10-driven CXCL8 induction, we conducted pharmacological inhibition experiments in 786-O cells. After 24 hours of serum starvation, the cells were pretreated with clinically relevant inhibitors targeting MEK (binimetinib, 5 μM), JNK (SP600125, 10 μM), and PI3K (LY294002, 20 μM) before FKBP10 stimulation. FKBP10 treatment significantly increased CXCL8 mRNA expression. Notably, binimetinib, a MEK inhibitor, strongly reduced FKBP10-induced CXCL8 expression, while the JNK and PI3K inhibitors had no effect (**Fig. 8A-B**). Further experiments showed that FKBP10 knockdown reduced phospho-MEK, phospho-ERK, and CXCL8 expression without affecting total MEK/ERK levels (**Fig. 8C**). Conversely, FKBP10 overexpression increased phospho-MEK, phospho-ERK, and CXCL8 expression, again without altering total MEK/ERK levels (**Fig. 8D**). These findings confirm that FKBP10 upregulates CXCL8 expression through the MEK/ERK signaling pathway in RCC cells.

### ELF3 Drives FKBP10-Mediated Transcriptional Activation of CXCL8

Integrative analysis of ENCODE ChIP-seq datasets identified 135 transcription factors (TFs) using MACS2 peak calling (q < 0.01). These TFs demonstrate coordinated chromatin binding at the CXCL8 locus, including active promoter regions (H3K4me3+/H3K27ac+) and distal enhancers (H3K4me1+) (**Fig. S32A**). Intersectional analysis with TCGA-KIRC differential expression profiles (|log2FC| > 1, FDR < 0.05) highlighted 18 clinically relevant TFs (**Fig. S32B**). Among these, only ELF3, ZNF331, and ATF3 showed significant co-expression with CXCL8 (*P* < 0.001) (**Fig. S32C**). Computational deconvolution using JASPAR confirmed that ELF3 binds to the CXCL8 promoter (-2,000 bp to +100 bp around TSS) with highly conserved motifs (relative score > 0.85/1.0) (**Fig. S32D**).

Western blot showed that FKBP10 knockdown reduced both ELF3 and CXCL8 protein levels (**Fig. 8E**). Rescue experiments showed that FKBP10 overexpression significantly increased CXCL8 expression. However, this effect was substantially diminished when ELF3 was also knocked down (**Fig. 8F**), indicating that FKBP10's induction of CXCL8 is ELF3-dependent. Conversely, FKBP10 knockdown led to a marked decrease in CXCL8 levels, but this reduction was reversed by ELF3 overexpression (**Fig. 8G**). Together, these results demonstrate that ELF3 is crucial for FKBP10-mediated CXCL8 expression. RNA-seq profiling (siFKBP10 vs. siNC, n = 3 biological replicates) confirmed the downregulation of the ELF3-CXCL8 axis, EMT master regulators, and M2 polarization effectors (**Fig. 8H**) ChIP-seq density and heatmaps showing FKBP10-associated chromatin occupancy around transcription start sites (±3 kb) in WT and FKBP10-knockdown (shFKBP10) cells. Compared to WT, FKBP10 depletion markedly reduced enrichment at TSS regions, indicating a loss of FKBP10-dependent promoter binding (**Fig. 8I**). Peak plots revealed a reduction in ELF3 binding to the CXCL8 promoter region in shFKBP10 cells compared to WT cells (**Fig. 8J**). Taken as a whole, FKBP10 activates the MEK/ERK/ELF3 signaling cascade to transcriptionally upregulate CXCL8, which is secreted into the TME. This paracrine CXCL8 drives M2 macrophage polarization, thereby fostering an immunosuppressive niche that accelerates tumor immune evasion and metastatic outgrowth (**Fig. 8K**).

### Synergistic Antitumor Effects of siELF3 and Anti-PD-1 Therapy

We investigated whether targeting ELF3 could enhance the effectiveness of ICB therapy. Using a syngeneic Renca renal carcinoma model in BALB/c mice (n = 5 per group), we tested the combination of ELF3 inhibition with anti-PD-1 therapy (**Fig. 8L**).

The combination of VNPsiELF3 and anti-PD-1 led to a significant reduction in tumor weight compared to the control groups (VNPsiNC + IgG, VNPsiELF3 + IgG, and VNPsiNC + anti-PD-1) (**Fig. 8M**). Tumor volume measurements over time showed slower tumor growth in the combination therapy group (**Fig. 8N**). The VNPsiELF3 + anti-PD-1 group showed the most significant tumor suppression. These results support the potential of combining siELF3 with anti-PD-1 therapy as a promising strategy for improving treatment outcomes in RCC.

## Discussion

This study systematically identified four TIME subtypes and six tumor cell states with distinct prognostic values in ccRCC by analyzing over one million single cells across ten cohorts. Additionally, through cell communication analysis, we delineated four distinct tumor ecosystems. We constructed a robust 126-gene prognostic model based on the ecosystem with the poorest prognosis. Mechanistically, we identified FKBP10 as a core pathogenic gene and revealed a novel mechanism by which it upregulates CXCL8 secretion through the MEK/ERK/ELF3 signaling axis, thereby remodeling the immunosuppressive microenvironment and promoting tumor progression.

The present study has provided several significant findings. First, our integrative analysis delineates four archetypes of TIME heterogeneity in ccRCC, revealing clinically associated biological features. The correction of batch effects and the inclusion of abundant non-malignant cell populations allowed us to uncover these previously unreported features 13, 14, 16-19, 21, 33. The CM1-IA subtype displayed an immunologically active phenotype linked to improved survival and a favorable response to the mTOR inhibitor everolimus. However, its limited predictive value for PD-1 blockade (nivolumab) outcomes likely reflects fundamental differences in therapeutic mechanisms. Everolimus primarily targets tumor-intrinsic pathways by inhibiting angiogenesis 61. In contrast, nivolumab's efficacy depends on the dynamic equilibrium within the TIME. Its clinical effectiveness is concurrently modulated by pre-existing effector T cells, T cell exhaustion 62, immunosuppressive cell infiltration, antigen presentation capacity, etc. 63. These coexisting regulatory networks may collectively diminish the prognostic significance of CM1-IA signatures in anti-PD-1 therapy. Contrastingly, the CM2-II subtype exhibited prominent innate immune activity. Since innate immune responses primarily modulate adaptive immunity rather than exert direct cytotoxic effects 64, 65, this may explain its weak association with prognosis and immunotherapy response in ccRCC. The CM3-ISM subtype, characterized by dense myeloid infiltration, was associated with poor prognosis and resistance to both mTOR inhibitors and PD-1 blockade, consistent with previous studies 8, 9. Notably, our comprehensive analysis revealed mechanisms of tumor progression and treatment resistance. In CM3-ISM, most macrophages express high levels of macrophage polarization regulators (e.g., APOC1, SELENOP) that have been shown to promote M2 polarization of macrophages and play a tumor-promoting role by regulating cancer cell proliferation, metastasis, and angiogenesis 66-68. Other macrophages overexpressing CCL3/CCL4 have been reported to augment tumor metastasis by promoting neovascularization, recruiting Tregs, and recruiting pro-tumorigenic macrophages via CCL3/CCL4-CCR5 axis 69, 70. The CM4-IE subtype, composed primarily of fibroblasts and endothelial cells, influences angiogenesis, cell adhesion, and migration. These cells have strong immunomodulatory capacities that contribute to immune evasion 62, 71. The CM4-IE signature and its associated improvement in immunotherapy response may be attributed to a spatially coordinated TME, where ICB has the potential to reverse T cell dysfunction by modulating cell-cell interactions 17, 72. Consequently, the CM4-IE signature may represent a contextual biomarker indicative of a TME susceptible to immune reactivation. Overall, this work provides the first comprehensive delineation of TIME heterogeneity in ccRCC, paralleling similar immune landscapes observed in liver cancer 39. This work advances precision medicine and immuno-oncology by helping identify patients who are responsive to immunotherapy. It provides targets and pathways for developing combination therapies to overcome future treatment resistance.

Second, we deciphered the intra-tumor expression heterogeneity of malignant epithelial cells and categorized ccRCC tumor cells into six states. A previous study has reported six conserved MPs that distinguish ccRCC tumor cell functions, including stress response, proximal tubule, EMT, cell death, MHC-II, and cell cycle 32. However, it only included ten samples, which may not adequately represent the full spectrum of ccRCC heterogeneity. These MPs align with those found in our analysis, except for proximal tubule, cell death, and MHC-II. The proximal tubule meta-program shared transcriptional features with our metabolic meta-program, while the MHC-II meta-program was similar to our antigen-presenting meta-program. Our analysis, which includes a larger sample size and diverse tumor cell populations, revealed a previously unreported angiogenic state. Patients in the angiogenic state demonstrated better responses to VEGF-targeting TKIs, revealing pathway-specific therapeutic efficacy 73. Classifying ccRCC tumor cells in the angiogenic state could be invaluable for identifying patients who are sensitive to TKI therapy. EMT tumor cells in ccRCC tended to localize to the tumor-normal interface, which is the leading and migratory edge of a tumor, potentially enabling the collective migration and invasion of tumor cells. High EMT scores in tumor cells correlate with increased metastatic potential, assisting in the identification of patients at higher risk for advanced disease. Given that immunotherapy is often reserved for advanced-stage ccRCC, this finding indirectly helps in selecting patients who may benefit from immunotherapeutic approaches. As for tumor cells in the metabolic and antigen-presenting state, our analysis indicated that these cells retained PT cell characteristics and antigen-presenting functions, suggesting a lower grade of malignancy and a more favorable prognosis. Moreover, the transcriptional features shared between cells in the metabolic state and the PT signature support the hypothesis that PT cells are a potential cell of origin for ccRCC 17. Our comprehensive scRNA-seq analysis, augmented by deconvoluted bulk RNA-seq, connects ITH with clinical heterogeneity. This connection is vital for understanding the progression and treatment response of ccRCC and for guiding the development of personalized therapeutic strategies.

Third, this study presents a tumor ecosystem framework that enhances our understanding of ccRCC progression and mechanisms of resistance to immunotherapy, enabling more accurate predictions of immunotherapy efficacy. Unlike traditional scoring systems that focus on the abundance of a single cell type, such as CD8+ T cells, our ecosystem-based classification offers distinct advantages. Single-cell infiltration scores fail to differentiate between functionally distinct cell states (e.g., exhausted vs. effector T cells) or capture the interactions among cells 74. For instance, a tumor with high CD8+ T cell infiltration and concurrent M2 macrophage enrichment (e.g., ecosystem 2) may exhibit a more severe immunosuppressive environment and worse prognosis compared to a tumor with lower infiltration but no such inhibitory factors (e.g., ecosystem 1). Our classification method integrates cell types, functional states, and their interactions, providing a more comprehensive view of the TME. This approach explains why ecosystem-based classification provides more accurate prognostic predictions. The four ecosystems identified in this study illuminate the variable clinical course of ccRCC. Specifically, myeloid immunosuppressive ecosystems, which correlate with poor prognosis, illustrate tumor immune evasion mechanisms: tumor cells promote invasion through EMT and suppress immune responses by recruiting MDSCs and polarizing M2 macrophages 75, 76. These two mechanisms work synergistically to create a formidable barrier to immunotherapy, explaining primary resistance to ICB. Our findings suggest that targeting specific components of these ecosystems, such as EMT or M2 macrophages, could improve treatment strategies. By shifting the perspective from a static “cell list” to a dynamic, interactive “ecosystem”, we provide a theoretical foundation for developing more precise prognostic models and combination therapies in ccRCC.

Fourth, our study not only identified FKBP10 as an oncogene in ccRCC, but also further revealed its novel downstream mechanism. FKBP10 has been found to be overexpressed in several cancers, including colorectal cancer, KRAS-mutant lung adenocarcinoma, renal cell carcinoma, and gastric cancer, and the knockdown of FKBP10 is sufficient to inhibit the proliferation of tumor cells 77-80. Consistent with these findings, our study shows that FKBP10 is highly expressed in ccRCC tissues, and its knockdown suppresses the malignant features of ccRCC cells both *in vitro* and *in vivo*. Interestingly, while the immunological role of FKBP10 in ccRCC has been little explored, we reveal its significant role in CXCL8 secretion via MEK/ERK activation, a well-characterized MAPK pathway involved in cell proliferation and survival 81. FKBP10 likely activates MEK/ERK through its PPIase domain, which catalyzes the cis/trans isomerization of proline residues, altering protein conformation, stability, and kinase activity 24, 25. Furthermore, we confirm that CXCL8 induces M2 macrophage polarization, aligning with prior studies 32, 82. This work provides a novel insight into how FKBP10 promotes tumor cell secretion of CXCL8, which in turn drives macrophage polarization toward the immunosuppressive M2 phenotype. This autocrine-paracrine loop sustains TME immunosuppression and may explain the correlation between high FKBP10 expression and disease progression in ccRCC patients. Given the well-characterized functional domains of FKBP10 80, the synergistic effect of siFKBP10 and anti-PD-1 therapy in our preclinical models indicates that FKBP10 might be used as a target in combination with anti-PD-1 regimens, to overcome resistance in tumors with high FKBP10 expression. Furthermore, our preclinical models also suggest that Reparixin, a CXCR1/2 antagonist currently in clinical trials 83, enhances the efficacy of anti-PD-1 therapy in ccRCC. Our study identifies several promising therapeutic targets that could offer new strategies to overcome treatment resistance in ccRCC, warranting further in-depth preclinical and clinical investigations.

Although our study provides new avenues for understanding ccRCC, several limitations must be acknowledged. First, in scRNA-seq data analysis, the removal of batch effects might inadvertently eliminate some biological signals. This issue, coupled with the limited sample size of our study, may result in an underestimation of heterogeneity in ccRCC. The functions of the identified CMs and tumor cell states are primarily based on bioinformatics analysis, and further experimental validation is required to confirm these findings. Second, the retrospective nature of our sample collection underscores the necessity for prospective validation in multicenter cohort studies to ensure the robustness and generalizability of our findings. This limitation highlights the potential for selection bias, as the datasets used may not be fully representative of the broader ccRCC patient population. It not only affects the model's stability but may also impact the generalizability of study results, reducing the model's performance on independent validation sets. Additionally, our study did not fully explore the mechanistic basis for the observed effects on immunotherapeutic efficacy, which warrants additional investigation. Furthermore, although the *in vitro* and *in vivo* experiments provide evidence for the role of FKBP10 in ccRCC, the lack of clinical trial data limits the translation of these findings into clinical practice. Future research should address these limitations by incorporating prospective data collection, improving data completeness and diversity, exploring relevant biological mechanisms, and validating our findings in larger and more diverse patient populations to enhance clinical utility. Besides, rigorous future clinical trials are essential to determine the safety and efficacy of combining immunotherapy with targeted FKBP10 therapy in ccRCC.

Building on existing research, this study systematically characterized the heterogeneity of the TME in ccRCC. Our integrated analysis of tumors and their microenvironment provides new insights into ccRCC progression and identifies potential targets for precision therapy. Notably, we uncovered a novel FKBP10-MEK/ERK-ELF3-CXCL8 signaling axis that plays a pivotal role in disease progression. Components of this pathway, including FKBP10, ELF3, CXCL8, and its receptor, represent promising therapeutic targets, warranting further in-depth preclinical and clinical research.

## Supplementary Material

Supplementary figures and materials and methods.

Supplementary tables.

## Abbreviations

ccRCC: clear cell renal cell carcinoma
TIME: tumor immune microenvironment
ISM: immunosuppressive myeloid cells
EMT: epithelial-mesenchymal transition
MDSC: myeloid-derived suppressor cell
ICB: immune checkpoint blockade
TME: tumor microenvironment
PPIase: peptidyl prolyl isomerases
scRNA-seq: single-cell RNA-sequencing
TCGA: The Cancer Genome Atlas
TKI: tyrosine kinase inhibitor
cNMF: consensus non-negative matrix factorization
GEP: gene expression program
MP: meta-program
ISM-EMT-Sig: immunosuppressive myeloid and epithelial mesenchymal transition interactive signature
ISM-EMT-RS: immunosuppressive myeloid and epithelial mesenchymal transition interactive risk score
Enet: Elastic Net
RSF: Random Survival Forest
SuperPC: Supervised Principal Component Analysis
plsRcox: Partial Least Squares Regression for Cox models
survival-SVM: Survival Support Vector Machine
GBM: Generalised Boosted Regression Modelling
DEG: differentially expressed gene
HR: hazard ratio
C-index: Harrell concordance index
VNP: vesicle-like PLGA-based nanoparticle
TSS: transcription start site
TF: transcriptional factors
TLS: tertiary lymphoid structure
CM: cellular modules
IA: immune activation
II: innate immunity
IE: immune exclusion
CAF: cancer-associated fibroblast
CNV: copy number variant
ITH: intra-tumor heterogeneity
OS: overall survival
PFS: progression-free survival
ROC: Receiver operating characteristic
