# Supplementary material for: FKBP10 promotes M2 polarization of macrophage via MEK/ERK/CXCL8 axis and facilitates tumor progression in clear cell renal cell carcinoma
Source: Int J Biol Sci. 2026 Jan 15;22(4):1807–33. doi: 10.7150/ijbs.117535 (PMC12905587; doi:10.7150/ijbs.117535)
Supplement: Supplementary file 1 — Supplementary figures and materials and methods. [file ijbsv22p1807s1.pdf]

Supplementary Materials

FKBP10 promotes M2 polarization of macrophage via MEK/ERK/CXCL8 axis and facilitates tumor progression in clear cell renal cell carcinoma

Supplementary Materials Checklist

<u>Supplementary Methods</u>

<u>Supplementary Tables</u>

Table S1 Key resources table

Table S2 Gene list of published immune-related signatures for the TIME classification.

Table S3 Gene lists of published 12 renal cell signatures for the Jaccard analysis.

Table S4 Clinical phenotype data of seven single-cell sequencing datasets utilized in the study.

Table S5 List of signature genes expressed in 74 defined cell clusters in ccRCC.

Table S6a Gene list of four TIME subtypes related signatures.

Table S6b Total results of GO and KEGG enrichment analysis of TIME related signatures.

Table S7 TIME subtypes of ccRCC patients.

Table S8a List of gene loadings within each gene expression programs that involved in meta-program 1.

Table S8b List of gene loadings within each gene expression programs that involved in meta-program 2.

Table S8c List of gene loadings within each gene expression programs that involved in meta-program 3.

Table S8d List of gene loadings within each gene expression programs that involved in meta-program 4.

Table S8e List of gene loadings within each gene expression programs that involved in meta-program 5.

Table S8f List of gene loadings within each gene expression programs that involved in meta-program 6.

Table S9 List of signature genes for ecosystem subtypes.

Table S10 Detail information of 15 genes involved in the optimal prognostic model.

<u>Supplementary Figures</u>

Figure S1 Flowchart summarizing the entire study design.

Figure S2 Quality control metrics of single-cell RNA sequencing data across all samples prior to filtering.

Figure S3 Quality control metrics for single-cell RNA sequencing data after filtering.

Figure S4 Comparative analysis of immune and stromal cell proportions between TLS-high and TLS-low groups.

Figure S5 Distinct characteristics of TIME subtypes.

Figure S6-S16 Gene expression within each gene expression program in each sample.

Figure S17 Hallmark pathways enrichment of six malignant meta-programs.

Figure S18 Distributions and clinical associations of six program tumor cells.

Figure S19 Analysis of ligand-receptor interactions in tumor ecosystems using CellphoneDB.

Figure S20 Ligand-receptor interactions, distribution, and signaling pathways across tumor ecosystems.

Figure S21 GSEA ridge plot analysis of tumor ecosystem signatures across GO, KEGG, and Hallmark gene sets.

Figure S22 Spatial co-localization analysis between EMT-high tumor cells and specific immune cell types.

Figure S23 NicheNet analysis and functional enrichment of the ecosystem 2 signature.

Figure S24. Assessment of prognostic model performance through forest plot and 10-fold cross-validation.

Figure S25 Forest plots of univariable and multivariable Cox regression analyses for the prognostic model across multiple cohorts.

Figure S26 Validation and evaluation of ISM-EMTRS prognostic potentials.

Figure S27 Validation of the role of FKBP10, DPEP1 and DNASE1L3 in ccRCC across 6 cohorts.

Figure S28 Association of FKBP10, DPEP1, and DNASE1L3 expression with survival outcomes in immunotherapy cohorts.

Figure S29 FKBP10 is an independent prognostic factor and remodels the tumor immune microenvironment in ccRCC.

Figure S30 Analysis of immune cell infiltration stratified by FKBP10 expression levels in E-MTAB-1980 and Checkmate cohorts.

Figure S31 Analysis of immune cell infiltration stratified by FKBP10 expression levels in TCGA-KIRC and JAVELIN cohorts.

Figure S32 Analysis of transcription factor binding and prioritization of a key regulator at the CXCL8 locus.

Supplementary Method

scRNA-seq Data Processing

The initial step involved the exclusion of peripheral blood mononuclear cell data to emphasize the tumor-infiltrating immune cells. Low quality cells (<300 or >5,000 genes detected; <500 UMIs; >20% (for tumor samples) or >80% (for benign samples) mitochondrial reads; >1% hemoglobin gene content) were also excluded before being merged together. The mitochondrial gene content restrictions were based on the observed mitochondrial read disparities in prior kidney scRNA-seq and bulk RNA-seq studies [1-4]. Subsequently, DoubletFinder was employed for potential doublet cell filtration [5]. Normalization was achieved using NormalizeData function, with 3,000 highly variable genes (HVGs) identified using the FindVariableFeatures function, and gene scaling to unit variance. Cell clusters were delineated through principal component analysis (PCA) of the top 20 principal components (PCs), integrating batch effect rectification via the harmony package. The Louvain method was employed for clustering, and dimensionality reduction was performed using Uniform Manifold Approximation and Projection (UMAP).

We annotated the six major cell types identified in our dataset on the basis of well-known marker genes, including CD3D, CD3E, CD8A, CD4, NKG7 for T lymphoid cells; CD79A, CD79B, CD19, MS4A1 for B lymphoid cells; C1QA, C1QB, C1QC, CD68, ITGAX for myeloid lineage; PECAM1, KDR, AQP1, PLVAP for endothelial cells; COL1AL, COL3A1, ACTA2, PDGFRB for fibroblast; EPCAM, PAX8, KRT18, KRT8, NNMT for epithelial cells. Among these epithelial cells, malignant cells were further distinguished from non-malignant cells by inferring large-scale CNVs using inferCNV. Non-malignant epithelial cells derived from normal samples were used as references for CNV estimation.

We conducted a second round of clustering to further characterize the subpopulations of major cell types. Owing to the variable amount and properties of cells in each major cell type, different parameters for clustering were used. For the clustering of NK or T cells, the top 20 PCs were selected based on 3,000 HVGs (resolution=0.8). For clustering of B cells, the top 15 PCs were selected on the basis of 2,000 HVGs (resolution=0.6). For myeloid cells, the top 20 PCs were selected on based on 3,000 HVGs (resolution=0.5). For endothelial cells, the top 15 PCs were selected based on 2,000 HVGs (resolution=0.4). For fibroblasts, the top 15 PCs were selected based on 1,000 HVGs (resolution=0.4).

As a result, 75 cell clusters were identified. Annotation relied on the most highly expressed immune-related genes in each cluster, with the gene list curated according to previous findings [6]. To facilitate data visualization, cells were re-clustered into five embeddings using Seurat, including (1) NK and T cells, (2) myeloid cells, (3) B cells, (4) endothelial cells, (5) fibroblast. Next, we used the FindAllMarkers function to identify differentially expressed genes (DEGs) with adjusted $P<0.05$ using the Bonferroni correction.

CytoTRACE Stemness Analysis

CytoTRACE was utilized to assess cellular stemness within the epithelial cluster, with higher gene counts in single cells indicating lesser differentiation and elevated stemness [7]. This algorithm aids in identifying genes

that significantly correlate with cellular gene counts and stemness, thereby enabling precise stemness scoring.

Immune Infiltration Analysis

To comprehensively characterize the immune cell composition in the tumor microenvironment, we applied multiple computational algorithms, including ESTIMATE, Cibersort, MCP-counter, EPIC, and xCell. These methods leverage distinct principles to infer immune cell abundances from bulk transcriptomic data, providing complementary insights into stromal, immune, and malignant cell proportions.

The ESTIMATE algorithm employs single-sample gene set enrichment analysis (ssGSEA) to generate stromal, immune, and combined ESTIMATE scores, reflecting the presence of stromal and immune cells in tumor tissues. Tumor purity was derived from the ESTIMATE score using the formula: Tumor Purity = cos (0.6049872018 + 0.0001467884 × ESTIMATE score) [8]. Cibersort utilizes support vector regression to deconvolute expression data against the LM22 leukocyte signature matrix, quantifying 22 immune cell subtypes [9]. MCP-counter calculates the geometric mean of marker gene expressions to estimate absolute abundances of 8 immune and 2 stromal cell types [10]. EPIC employs constrained least squares regression to infer proportions of immune and cancer cells [11], while xCell applies ssGSEA-based enrichment to score 64 immune and stromal cell types [12]. The integration of these methods ensures robust and complementary evaluation of immune infiltration.

Sample Collection and Cell Culture

ccRCC and corresponding para-carcinoma tissues were collected directly from the operating room during nephrectomy. At the time of specimen extraction, samples of around 1-1.5 cm were obtained by the treating surgeon from tumor regions and para-tumor regions (at least 1 cm apart). Once in the operating room, small tissue aliquots (of around 5 mm^3) were separated from each sample for subsequent experiments. One aliquot was placed in 10% formalin for immunohistochemistry (IHC). The remainder of the tissue was placed in liquid nitrogen and transported on regular ice to the laboratory for subsequent quantitative real-time polymerase chain reaction (qPCR) and western blotting analysis.

The human renal cell carcinoma cell lines 769-P, 786-O and OS-RC-2 were used for experimental validation. All cell lines were cultured in RPMI1640 Medium, supplemented with 10% Fetal Bovine Serum (FBS) and 1% penicillin/streptomycin, and were maintained in an incubator at 37℃ and 5% CO2. Cell lines were sub-cultured by detaching cells using 0.25% trypsin EDTA, quenching, washing, and resuspension of the cell pellet in fresh media.

Quantitative Real-time Polymerase Chain Reaction

Tissues were homogenized and incubated for the indicated times with either compound or vehicle, and RNA was isolated using a universal RNA purification kit. cDNA was generated using a reverse transcription kit. qPCR was executed in triplicate using SYBR Green Master Mix on Quant-Studio 5 System. Gene expression

levels were quantitatively assessed with the method of $2^{-\Delta\Delta Ct}$.

Protein Extraction and Western Blot Analysis

For proteins extraction, tissues or cells were homogenized in RIPA lysis buffer containing 150 mM NaCl, 1.0% IGEPAL CA-630, 0.5% sodium deoxycholate, 0.1% SDS, and 50 mM Tris (pH 8.0), supplemented with protease and phosphatase inhibitors, and incubated on ice for 30 minutes to ensure complete lysis. The homogenates were then centrifuged to remove the cellular debris. The protein concentration in the supernatants was quantified using a BCA Protein Assay Kit. For electrophoresis, lysates were prepared by mixing 2× sample buffer (0.125 M Tris-HCl, pH 6.8, 4% SDS, 20% glycerol) enriched with 4% β-mercaptoethanol, followed by denaturation at 100°C for 10 minutes. Denatured proteins were resolved on SDS-PAGE gels and subsequently transferred onto PVDF membranes. Non-specific binding sites on the membranes were blocked with 5% skim milk at room temperature. Primary antibodies against FKBP10 (1:5000), DNASE1L3 (1:1000), DPEP1 (1:2000), MEK (1:10000), ERK (1:3000), p-MEK (1:1000), p-ERK (1:2000), ELF3 (1:200), Vinculin (1:20000), and CXCL8 (1:1000) were applied to the blocked membranes and incubated overnight at 4°C. After incubating with horseradish peroxidase-conjugated secondary antibodies, the protein bands were visualized using the Amersham ImageQuant 800 imaging system.

Immunohistochemical Staining

Formalin-fixed, paraffin-embedded (FFPE) sections of ccRCC tissues (4 μm thickness) were subjected to IHC staining using standard protocols. Briefly, sections were deparaffinized in xylene and rehydrated through a graded ethanol series. Antigen retrieval was performed by incubating slides in citrate buffer (pH 6.0) at 95°C using a microwave heating system. Endogenous peroxidase activity was quenched with 3% hydrogen peroxide, followed by blocking with 5% BSA.

Primary antibodies against FKBP10 (1:200), DNASE1L3 (1:200), DPEP1 (1:1000), N-cadherin (1:4000), Vimentin (1:5000), and E-cadherin (1:5000) were applied to tissue sections and incubated overnight at 4°C. After washing with PBS, slides were incubated with HRP-conjugated secondary antibodies. Diaminobenzidine (DAB) was used as the chromogen, and hematoxylin was applied for nuclear counterstaining.

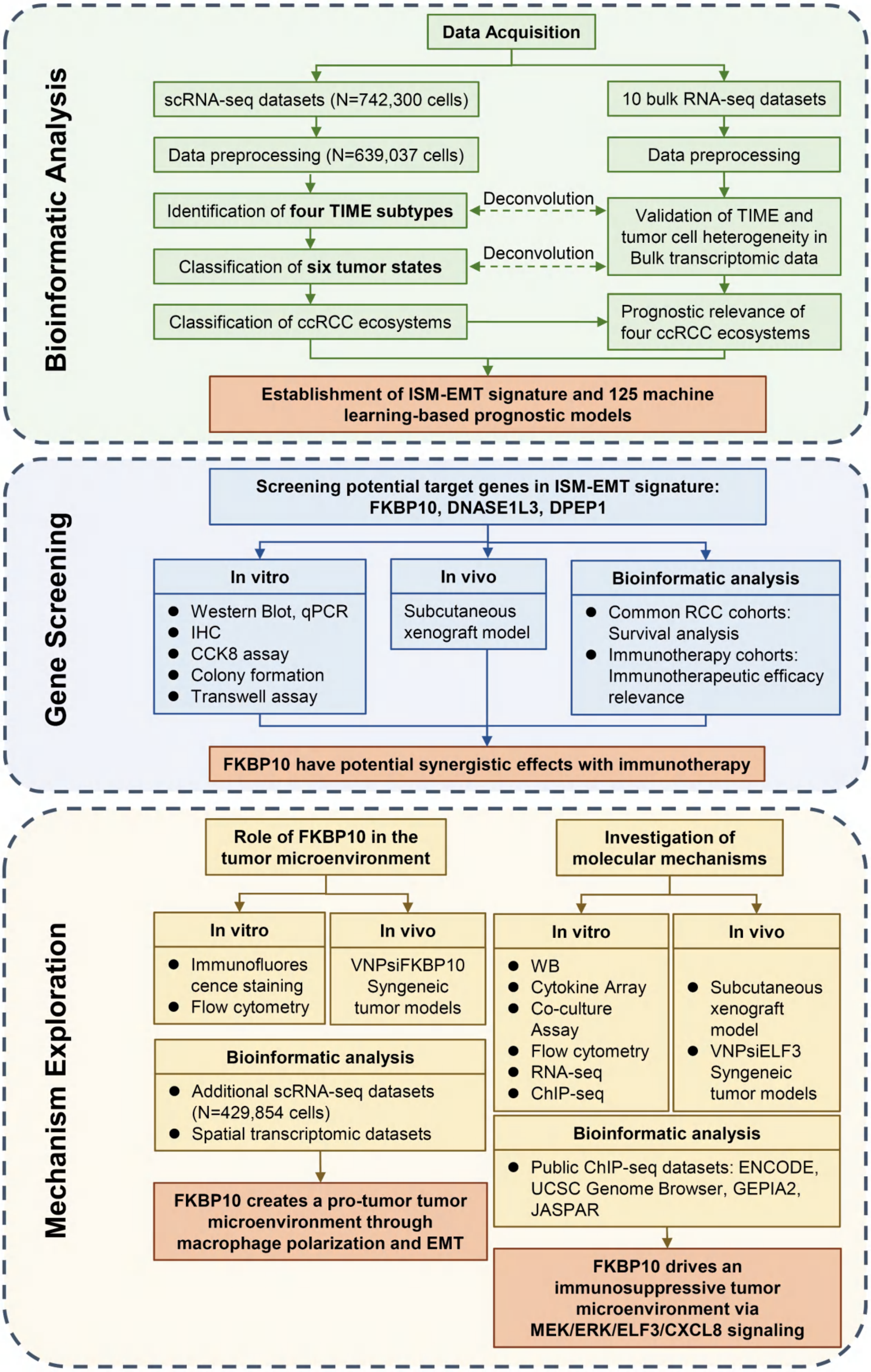

Figure S1 Flowchart summarizing the entire study design.

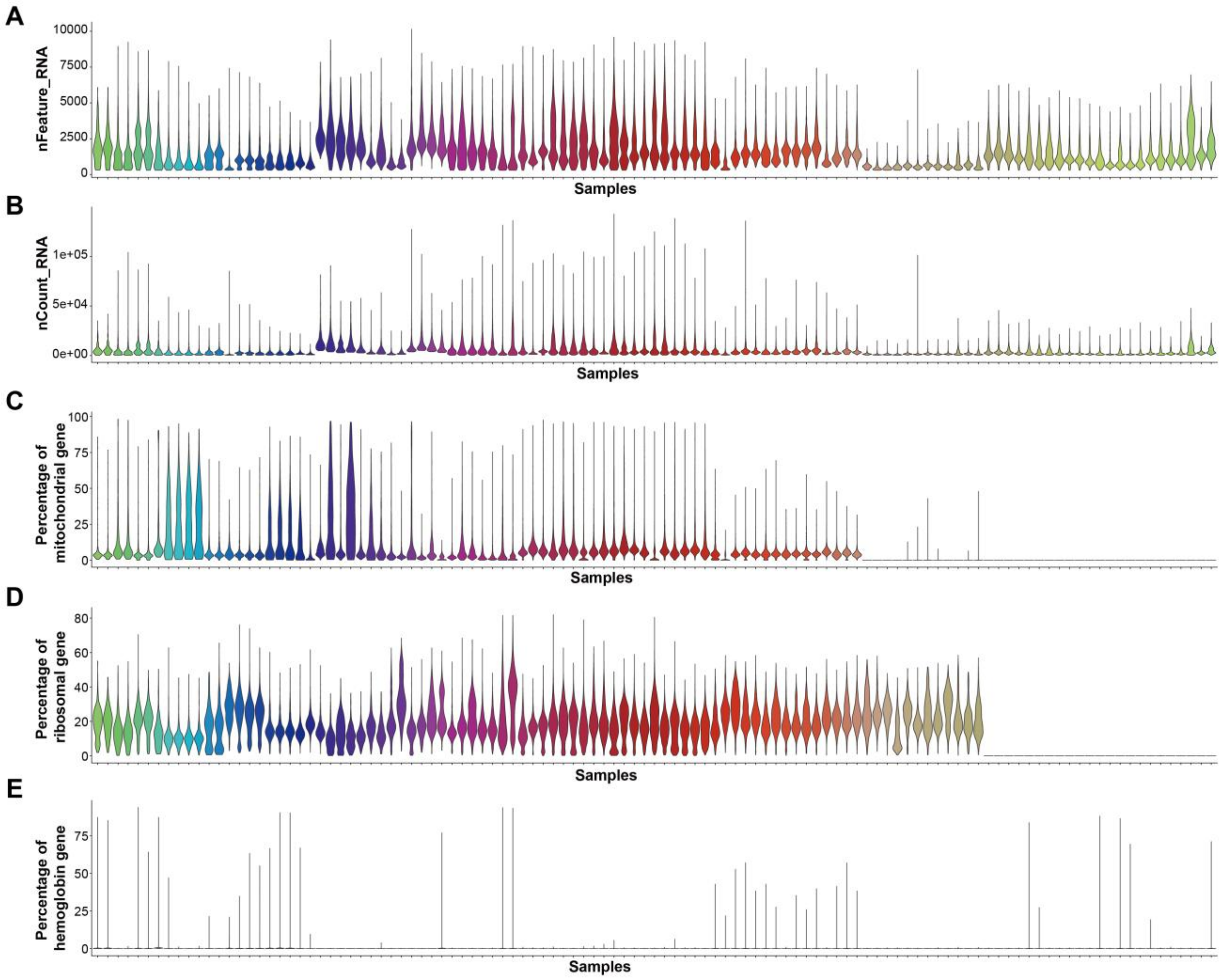

Figure S2 Quality control metrics of single-cell RNA sequencing data across all samples prior to filtering. Violin plots display the distribution of five key QC metrics for each sample: (A) nFeature_RNA: the number of unique genes detected per cell. A low count may indicate a damaged or low-quality cell. (B) nCount_RNA: the total number of molecules (UMIs) detected per cell. Extreme values (very low or very high) can suggest poor cell quality or the presence of multiple cells (doublets), respectively. (C) Percentage of mitochondrial gene: the percentage of cellular transcripts mapping to the mitochondrial genome. A high percentage is indicative of cellular stress or apoptosis. (D) Percentage of ribosomal gene: the percentage of transcripts mapping to ribosomal protein genes. (E) Percentage of hemoglobin gene: the percentage of transcripts mapping to hemoglobin genes. The shape of each violin represents the kernel density estimation of the data distribution. These metrics are crucial for identifying and removing low-quality cells to prevent technical artifacts from influencing downstream analyses.

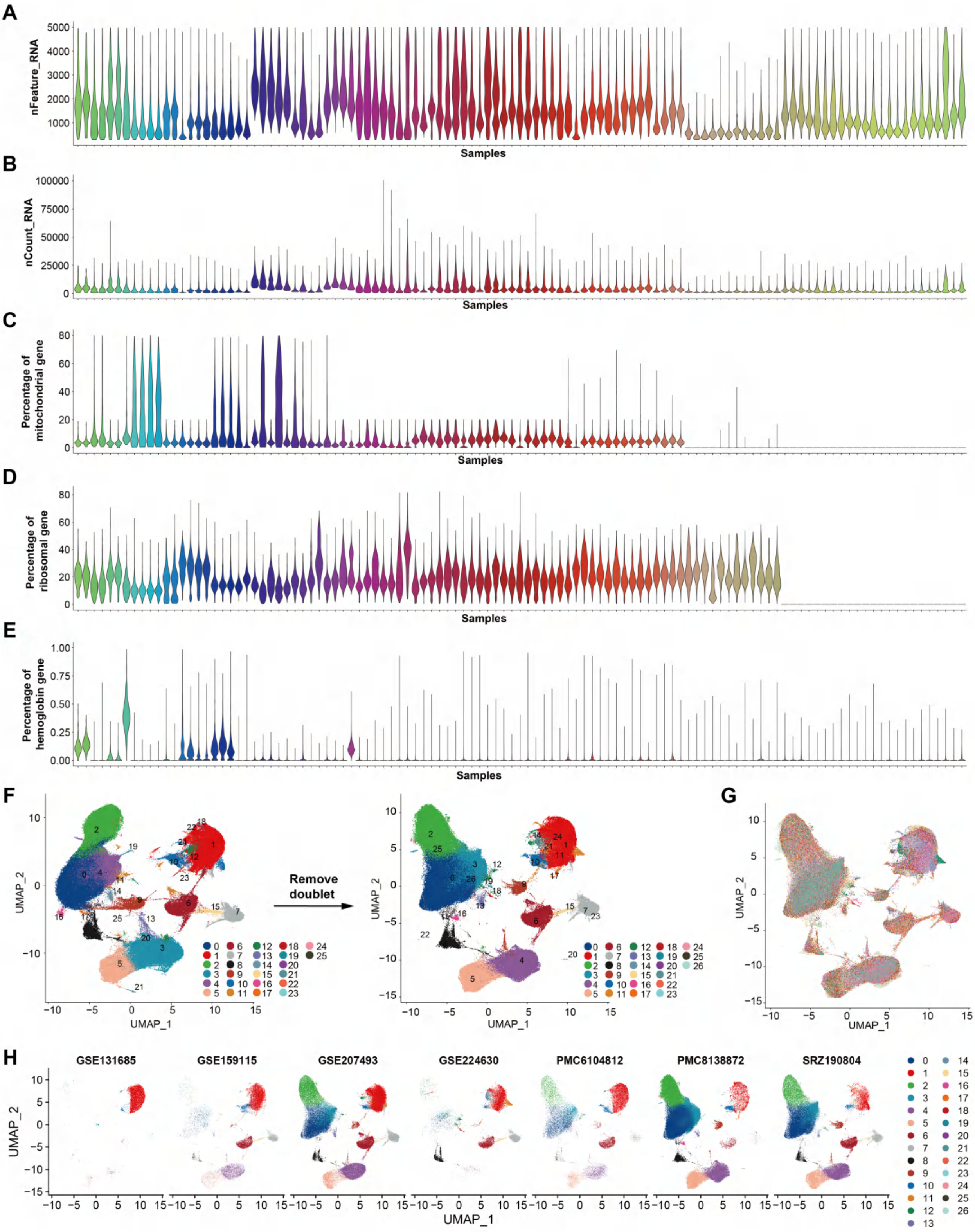

Figure S3 Quality control metrics for single-cell RNA sequencing data after filtering.

(A-E) Violin plots display the distribution of key QC metrics across all samples after quality filtering. Violin plots show number of detected genes per cell (nFeature_RNA), total UMI counts per cell (nCount_RNA), and the percentage of reads mapping to mitochondrial, ribosomal, and hemoglobin genes. Cells falling outside defined thresholds for these parameters were filtered to ensure data quality for downstream analysis. (F) UMAP plot showing clusters of all cells from seven public datasets before doublet cell filtration (left), and after doublet cell filtration (right). Dots represent individual cells, and colors represent different clusters. (G)

UMAP plot showing clusters of all cells, with color coded by samples. It can be seen that the Harmony algorithm effectively eliminates the batch effect of different samples. (H) UMAP visualization after Harmony integration, with cells split by dataset. Harmony effectively aligns datasets by removing technical variations while preserving biological structures, as evidenced by the overlapping distribution of analogous cell populations across samples.

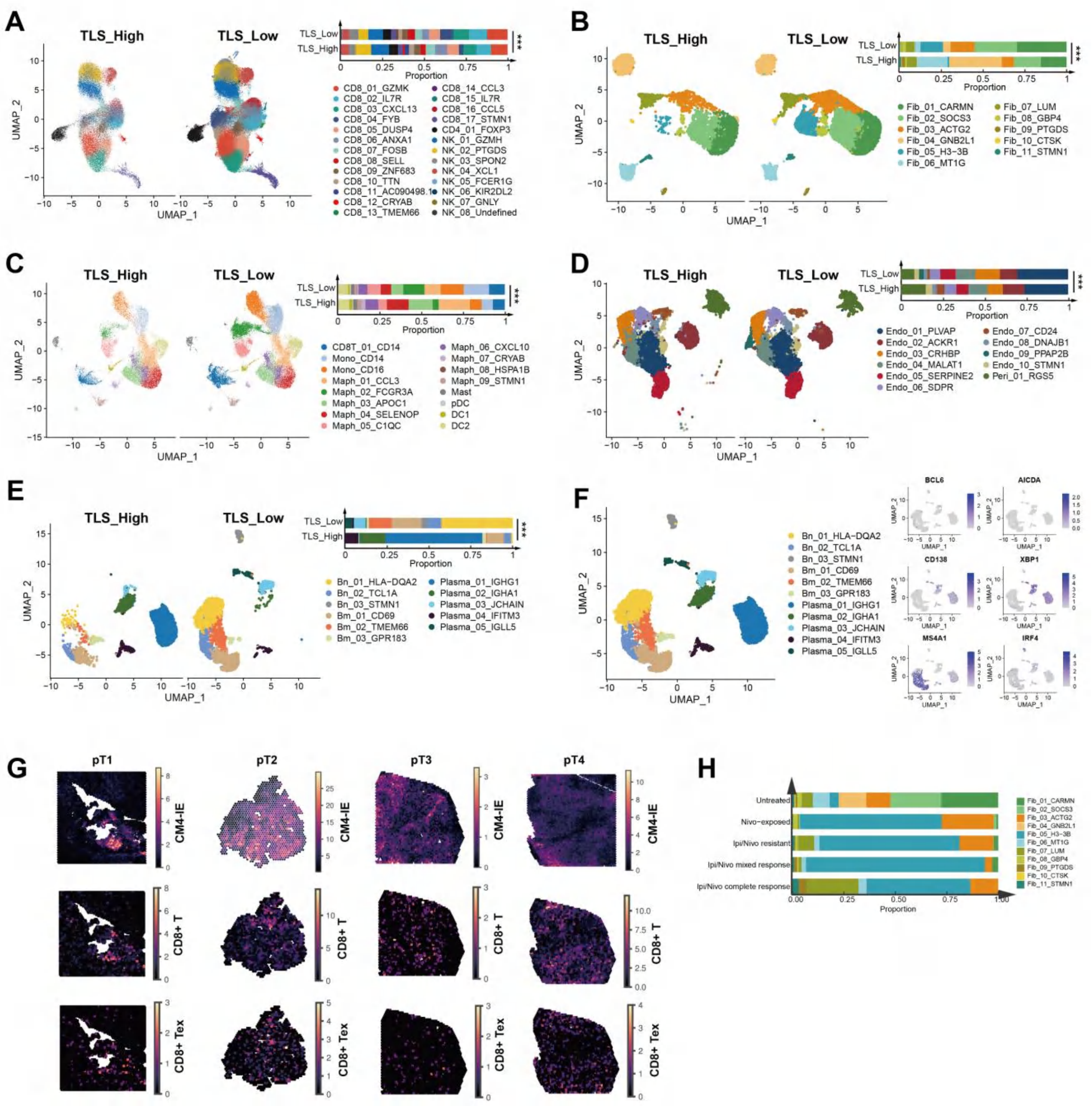

Figure S4 Comparative analysis of immune and stromal cell proportions between TLS-high and TLS-low groups.

(A-E) DimPlot illustrating the distribution of NK/T cells (A), fibroblasts (B), myeloid cells (C), endothelial cells (D), and B cells (E) across TLS-high and TLS-low groups. Cells are colored by cell type, and groups are segregated based on TLS signature scores. Stacked bar plot quantifying the relative proportions of each cell type within TLS-high and TLS-low groups. The B-cell compartment demonstrates the most notable shift, with

altered subset composition in TLS-high samples. Statistical significance was assessed using Chi-square test. TLS, tertiary lymphoid structure. (F) FeaturePlot visualization of germinal center (GC) gene expression levels for BCL6, AICDA, MS4A1, CD138, XBP1, and IRF4 across the B-cell subsets. Expression gradients are depicted from low (gray) to high (blue). (G) Spatial co-localization analysis between CM4-IE cells cells and specific T cell types. Representative spatial transcriptomics map showing the distribution of CM4-IE-high cells, CD8+ T cells, and exhausted CD8+ T cells within the tumor microenvironment. Color gradient indicates signature score intensity. CM4-IE-high cells exhibit spatial proximity to CD8+ T cells and exhausted CD8+ T cells. (H) Immunotherapeutic relevance of fibroblast clusters. Stacked histogram showing the fibroblast clusters proportion in ICB-treated and ICB-untreated cohort. Fib_05_H3-3B was found to significantly enriched in ICB-treated samples. Chi-square test was used for statistical analysis. Nivo, Nivolumab (anti-PD-1 monotherapy); Ipi, Ipilimumab (anti-CTLA-4 monotherapy).

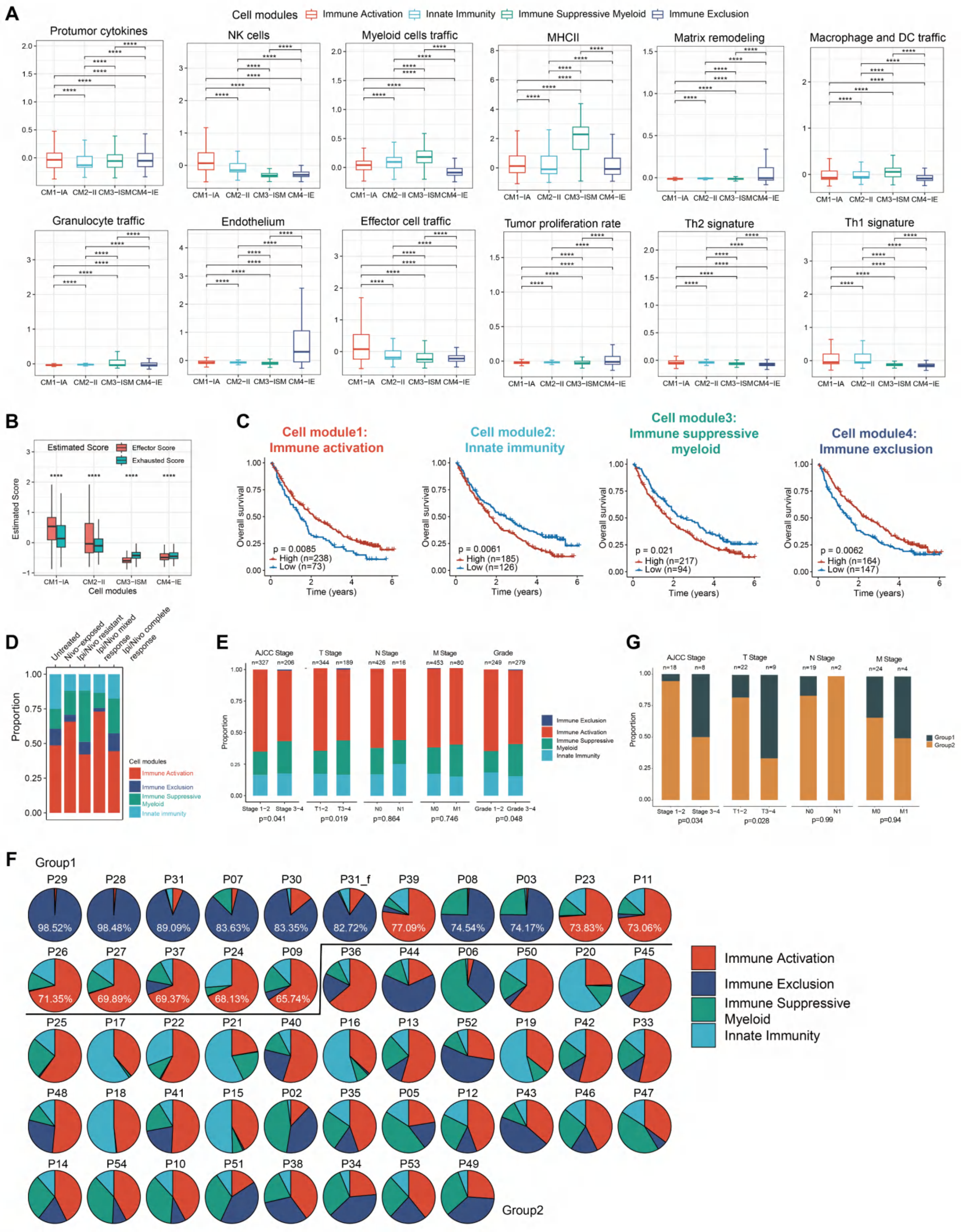

Figure S5 Distinct characteristics of TIME subtypes.

(A) Boxplots showing the expression of supplementary immune-related signatures in different TIME subtypes. Wilcoxon rank-sum test (two-sided) was applied for significance test. *, $P < 0.05$; **, $P < 0.01$; ***, $P < 0.001$.

IA, immune activation; II, innate immunity; ISM, immune suppressive myeloid; IE, immune exclusion. (B) Boxplots showing the expression of exhausted and effector signatures in different TIME subtypes. Wilcoxon rank-sum test (two-sided) was applied for significance test. *, $P < 0.05$; **, $P < 0.01$; ***, $P < 0.001$. (C) Overall survival (OS) of cases stratified by BisqueRNA predicted TIME-specific cell types proportion in Checkmate cohort (including all samples). Log-rank test was used for statistical analysis. (D) Stacked histogram showing the TIME proportion in ICB-treated cohort. (E) Stacked histograms showing TIME-specific cell types proportion across different tumor stages and pathological grades. Cell types proportion were predicted by BisqueRNA in TCGA-KIRC cohort. Chi-square test was used for statistical analysis. (F) Pie charts showing the proportion of TIME specific cell types across 52 patients. Patients with cells (≥65%) from sole individual TIME subtype were classified as group 1 (monotypic TIME dominance) (n = 16), while patients with cellular composition shared by multiple TIME subtypes were classified as group 2 (heterotypic TIME integration) (n = 36). (G) Stacked histograms showing group cluster proportion across different tumor stages in our integrated scRNA-seq datasets. Chi-square test was used for statistical analysis.

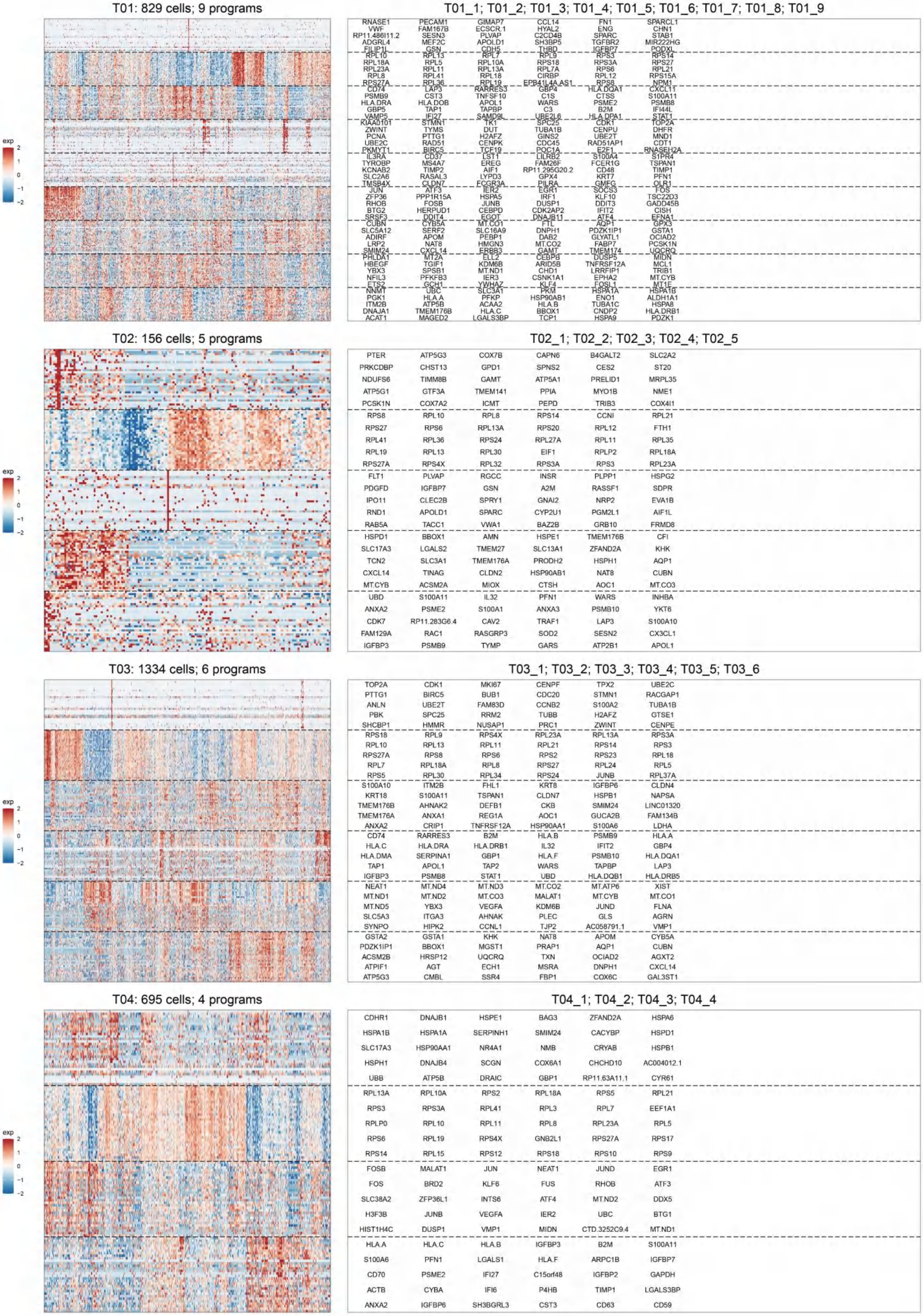
T01: 829 cells; 9 programs
exp
2
1
0
−1
−2
T01_1; T01_2; T01_3; T01_4; T01_5; T01_6; T01_7; T01_8; T01_9
RNASE1 PECAM1 GIMAP7 CCL14 FN1 SPARCL1
VWF FAM167B ECSCR.1 HYAL2 ENG CHN1
RP11.486I11.2 SESN3 PLVAP C2CD4B SPARC STAB1
ADGRL4 MEF2C APOLD1 SH3BP5 TGFBR2 MIR222HG
FILIP1L GSN CDH5 THBD IGFBP7 PODXL
RPL10 RPL13 RPL7 RPL9 RPS3 RPS14
RPL18A RPL5 RPL10A RPS18 RPS3A RPS27
RPL23A RPL11 RPL13A RPL7A RPS6 RPL21
RPL8 RPL41 RPL18 CIRBP RPL12 RPS15A
RPS27A RPL36 RPL19 EPB41L4A.AS1 RPS8 NPM1
CD74 LAP3 RARRES3 GBP4 HLA.DQA1 CXCL11
PSMB9 CST3 TNFSF10 C1S CTSS S100A11
HLA.DRA HLA.DOB APOL1 WARS PSME2 PSMB8
GBP5 TAP1 TAPBP C3 B2M IFI44L
VAMP5 IFI27 SAMD9L UBE2L6 HLA.DPA1 STAT1
KIAA0101 STMN1 TK1 SPC25 CDK1 TOP2A
ZWINT TYMS DUT TUBA1B CENPU DHFR
PCNA PTTG1 H2AFZ GINS2 UBE2T MND1
UBE2C RAD51 CENPK CDC45 RAD51AP1 CDT1
PKMYT1 BIRC5 TCF19 POC1A E2F1 RNASEH2A
IL3RA CD37 LST1 LILRB2 S100A4 S1PR4
TYROBP MS4A7 EREG FAM26F FCER1G TSPAN1
KCNAB2 TIMP2 AIF1 RP11.295G20.2 CD48 TIMP1
SLC2A6 RASAL3 LYPD3 GPX4 KRT7 PFN1
TMSB4X CLDN7 FCGR3A PILRA GMFG OLR1
JUN ATF3 IER2 EGR1 SOCS3 FOS
ZFP36 PPP1R15A HSPA5 IRF1 KLF10 TSC22D3
RHOB FOSB JUNB DUSP1 DDIT3 GADD45B
BTG2 HERPUD1 CEBPD CDK2AP2 IFIT2 CISH
SRSF3 DDIT4 EGOT DNAJB11 ATF4 EFNA1
CUBN CYB5A MT.CO1 FTL AQP1 GPX3
SLC5A12 SERF2 SLC16A9 DNPH1 PDZK1IP1 GSTA1
ADIRF APOM PEBP1 DAB2 GLYATL1 OCIAD2
LRP2 NAT8 HMGN3 MT.CO2 FABP7 PCSK1N
SMIM24 CXCL14 ERBB3 GAMT TMEM174 UQCRQ
PHLDA1 MT2A ELL2 CEBPB DUSP5 MIDN
HBEGF TGIF1 KDM6B ARID5B TNFRSF12A MCL1
YBX3 SPSB1 MT.ND1 CHD1 LRRFIP1 TRIB1
NFIL3 PFKFB3 IER3 CSNK1A1 EPHA2 MT.CYB
ETS2 GCH1 YWHAZ KLF4 FOSL1 MT1E
NNMT UBC SLC3A1 PKM HSPA1A HSPA1B
PGK1 HLA.A PFKP HSP90AB1 ENO1 ALDH1A1
ITM2B ATP5B ACAA2 HLA.B TUBA1C HSPA8
DNAJA1 TMEM176B HLA.C BBOX1 CNDP2 HLA.DRB1
ACAT1 MAGED2 LGALS3BP TCP1 HSPA9 PDZK1
T02: 156 cells; 5 programs
exp
2
1
0
−1
−2
T02_1; T02_2; T02_3; T02_4; T02_5
PTER ATP5G3 COX7B CAPN6 B4GALT2 SLC2A2
PRKCDBP CHST13 GPD1 SPNS2 CES2 ST20
NDUFS6 TIMM8B GAMT ATP5A1 PRELID1 MRPL35
ATP5G1 GTF3A TMEM141 PPIA MYO1B NME1
PCSK1N COX7A2 ICMT PEPD TRIB3 COX4I1
RPS8 RPL10 RPL8 RPS14 CCNI RPL21
RPS27 RPS6 RPL13A RPS20 RPL12 FTH1
RPL41 RPL36 RPS24 RPL27A RPL11 RPL35
RPL19 RPL13 RPL30 EIF1 RPLP2 RPL18A
RPS27A RPS4X RPL32 RPS3A RPS3 RPL23A
FLT1 PLVAP RGCC INSR PLPP1 HSPG2
PDGFD IGFBP7 GSN A2M RASSF1 SDPR
IPO11 CLEC2B SPRY1 GNAI2 NRP2 EVA1B
RND1 APOLD1 SPARC CYP2U1 PGM2L1 AIF1L
RAB5A TACC1 VWA1 BAZ2B GRB10 FRMD8
HSPD1 BBOX1 AMN HSPE1 TMEM176B CFI
SLC17A3 LGALS2 TMEM27 SLC13A1 ZFAND2A KHK
TCN2 SLC3A1 TMEM176A PRODH2 HSPH1 AQP1
CXCL14 TINAG CLDN2 HSP90AB1 NAT8 CUBN
MT.CYB ACSM2A MIOX CTSH AOC1 MT.CO3
UBD S100A11 IL32 PFN1 WARS INHBA
ANXA2 PSME2 S100A1 ANXA3 PSMB10 YKT6
CDK7 RP11.283G6.4 CAV2 TRAF1 LAP3 S100A10
FAM129A RAC1 RASGRP3 SOD2 SESN2 CX3CL1
IGFBP3 PSMB9 TYMP GARS ATP2B1 APOL1
T03: 1334 cells; 6 programs
exp
2
1
0
−1
−2
T03_1; T03_2; T03_3; T03_4; T03_5; T03_6
TOP2A CDK1 MKI67 CENPF TPX2 UBE2C
PTTG1 BIRC5 BUB1 CDC20 STMN1 RACGAP1
ANLN UBE2T FAM83D CCNB2 S100A2 TUBA1B
PBK SPC25 RRM2 TUBB H2AFZ GTSE1
SHCBP1 HMMR NUSAP1 PRC1 ZWINT CENPE
RPS18 RPL9 RPS4X RPL23A RPL13A RPS3A
RPL10 RPL13 RPL11 RPL21 RPS14 RPS3
RPS27A RPS8 RPS6 RPS2 RPS23 RPL18
RPL7 RPL18A RPL8 RPS27 RPL24 RPL5
RPS5 RPL30 RPL34 RPS24 JUNB RPL37A
S100A10 ITM2B FHL1 KRT8 IGFBP6 CLDN4
KRT18 S100A11 TSPAN1 CLDN7 HSPB1 NAPSA
TMEM176B AHNAK2 DEFB1 CKB SMIM24 LINC01320
TMEM176A ANXA1 REG1A AOC1 GUCA2B FAM134B
ANXA2 CRIP1 TNFRSF12A HSP90AA1 S100A6 LDHA
CD74 RARRES3 B2M HLA.B PSMB9 HLA.A
HLA.C HLA.DRA HLA.DRB1 IL32 IFIT2 GBP4
HLA.DMA SERPINA1 GBP1 HLA.F PSMB10 HLA.DQA1
TAP1 APOL1 TAP2 WARS TAPBP LAP3
IGFBP3 PSMB8 STAT1 UBD HLA.DQB1 HLA.DRB5
NEAT1 MT.ND4 MT.ND3 MT.CO2 MT.ATP6 XIST
MT.ND1 MT.ND2 MT.CO3 MALAT1 MT.CYB MT.CO1
MT.ND5 YBX3 VEGFA KDM6B JUND FLNA
SLC5A3 ITGA3 AHNAK PLEC GLS AGRN
SYNPO HIPK2 CCNL1 TJP2 AC058791.1 VMP1
GSTA2 GSTA1 KHK NAT8 APOM CYB5A
PDZK1IP1 BBOX1 MGST1 PRAP1 AQP1 CUBN
ACSM2B HRSP12 UQCRQ TXN OCIAD2 AGXT2
ATPIF1 AGT ECH1 MSRA DNPH1 CXCL14
ATP5G3 CMBL SSR4 FBP1 COX6C GAL3ST1
T04: 695 cells; 4 programs
exp
2
1
0
−1
−2
T04_1; T04_2; T04_3; T04_4
CDHR1 DNAJB1 HSPE1 BAG3 ZFAND2A HSPA6
HSPA1B HSPA1A SERPINH1 SMIM24 CACYBP HSPD1
SLC17A3 HSP90AA1 NR4A1 NMB CRYAB HSPB1
HSPH1 DNAJB4 SCGN COX6A1 CHCHD10 AC004012.1
UBB ATP5B DRAIC GBP1 RP11.63A11.1 CYR61
RPL13A RPL10A RPS2 RPL18A RPS5 RPL21
RPS3 RPS3A RPL41 RPL3 RPL7 EEF1A1
RPLP0 RPL10 RPL11 RPL8 RPL23A RPL5
RPS6 RPL19 RPS4X GNB2L1 RPS27A RPS17
RPS14 RPL15 RPS12 RPS18 RPS10 RPS9
FOSB MALAT1 JUN NEAT1 JUND EGR1
FOS BRD2 KLF6 FUS RHOB ATF3
SLC38A2 ZFP36L1 INTS6 ATF4 MT.ND2 DDX5
H3F3B JUNB VEGFA IER2 UBC BTG1
HIST1H4C DUSP1 VMP1 MIDN CTD.3252C9.4 MT.ND1
HLA.A HLA.C HLA.B IGFBP3 B2M S100A11
S100A6 PFN1 LGALS1 HLA.F ARPC1B IGFBP7
CD70 PSME2 IFI27 C15orf48 IGFBP2 GAPDH
ACTB CYBA IFI6 P4HB TIMP1 LGALS3BP
ANXA2 IGFBP6 SH3BGRL3 CST3 CD63 CD59

T05: 1051 cells; 5 programs

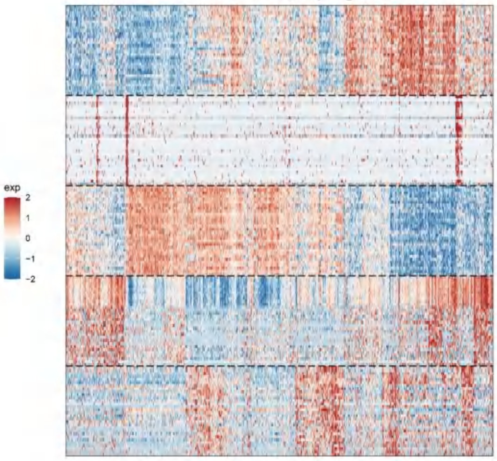

T05_1; T05_2; T05_3; T05_4; T05_5

BAG3	HSPH1	HSPD1	HSP90AB1	XBP1	HSPB1
CRYAB	ZFAND2A	DNAJB4	DDX5	DNAJA1	SELK
HSPE1	DNAJB1	CLK1	HSP90AA1	HSPA5	MCL1
CCNL1	SERPINH1	DDIT3	HSPA1B	PPP1R15A	HSPA8
ATF3	EGR1	HSPA4	UBC	DEDD2	H3F3B
UBE2C	CCNB2	CCNB1	CENPF	NEK2	PLK1
TOP2A	PTTG1	NUF2	HMMR	STMN1	UBE2T
CDK1	H2AFZ	CDCA8	CEP55	MKI67	HJURP
PRC1	AURKB	ASPM	NMU	CDC20	CKAP2
KIF20A	CDCA3	TPX2	FAM64A	CENPW	ARL6IP1
ALDOA	EEF1A1	PKM	RPL10A	BST2	RPL3
PGAM1	TMEM59	TPI1	HINT1	RPS3A	RPL4
RPL7	GAPDH	RPL5	SKP1	LAPTM4A	RPL21
RPL13A	PGK1	HLA.C	RPS5	GNB2L1	LDHB
FGG	RPS3	RPS27A	EIF3E	RPS2	RPS4Y1
MT.ND5	MT.ATP6	MT.ND4	MT.ND1	MT.CO2	MT.ND2
MT.CO1	MT.CYB	MT.ND3	MT.CO3	MT.ND4L	ST5
KCNQ1OT1	RRBP1	POLR2J3	INTS6	PLCG2	C1orf186
AGRN	RGS16	RP11.467L13.7	CSAD	RP11.412D9.4	LINC01320
RPL22L1	PAX2	CH17.189H20.1	HILPDA	RP11.796E2.4	HOOK2
MT2A	MT1E	PHLDA2	PHLDA1	LRRFIP1	CEBPB
MT1X	S100A16	PIM3	YWHAQ	CFLAR	TM4SF1
SERPINE1	FAM110C	FTH1	MAP1B	SMIM3	ODC1
S100A10	G0S2	FOXC2	FKBP5	SOD2	TXNRD1
BIRC3	DUSP5	TUBB2A	MSC	BHLHE40	PTPN1

T07: 1161 cells; 7 programs

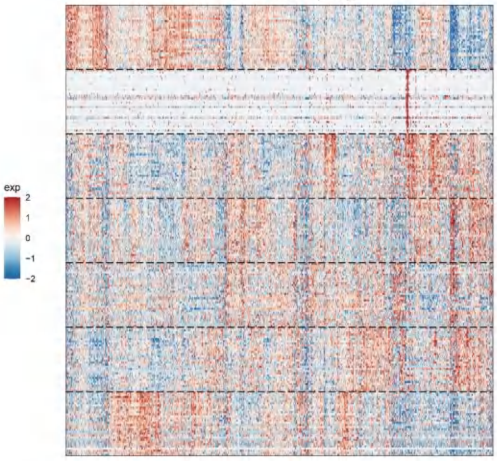

T07_1; T07_2; T07_3; T07_4; T07_5; T07_6; T07_7

RPS4X	RPS6	RPL10A	RPS14	RPL18A	RPS3
RPL10	NPM1	RPS2	GNB2L1	RPS15A	RPL23A
RPL11	RPL41	RPS18	RPL7A	RPS3A	RPL14
RPL35A	RPL6	RPL13	RPS23	RPL3	RPL5
RPL31	RPS13	BTF3	RPS7	RPL18	RPL12
PTTG1	MKI67	NEK2	UBE2C	TOP2A	HJURP
TROAP	KIF14	SPC25	CDKN3	RP11.844P9.2	ASPM
CCNB1	HIST1H4C	CENPF	UBE2T	CENPA	STMN1
PBK	HMMR	CDC20	NUF2	TUBA1B	CDCA3
CENPW	BIRC5	CDK1	KIF20A	NUSAP1	PLK1
S100A11	MT2A	S100A10	TIMP1	ANXA2	FTH1
MT1X	EMP3	S100A6	TMSB10	LGALS1	CD63
CYBA	SPP1	PKM	GAPDH	S100A16	CRYAB
IL32	SERPINA1	CLIC1	ACTB	MT1E	IGFBP3
PLP2	HLA.A	CD44	SERPINE2	PFN1	ENO1
NFKBIA	EGR1	CXCL2	SDC4	IER3	PPP1R15A
FOSB	ATF3	IRF1	NFKBIZ	MCL1	NFKBID
TNFAIP3	MAFF	JUNB	BTG2	C11orf96	JUND
PNRC1	CYLD	FSTL3	TNFAIP2	CXCL8	MIDN
ICAM1	SOD2	CXCL3	XBP1	ABL2	GEM
CP	UQCRB	MALAT1	FGB	AC159540.1	VCAN
VEGFA	ABCC3	IRS2	PNISR	ANKRD12	N4BP2L2
NEAT1	SON	APOB	NPTX2	RPL39	PDK4
NAP1L1	HSP90B1	HSPE1	RPS24	TXNIP	KLF6
SPINK13	DDX17	ADM	KCNQ1OT1	GOLGA4	RP5.1021I20.2
KHK	CYB5A	CD74	LGALS2	HLA.DRA	NNMT
HLA.A.1	HLA.DPA1	B2M	TMEM176B	RBP4	TMEM176A
HLA.DRB1	CXCL14	MYH8	SEPP1	HLA.C	TMEM37
REG1A	NDUFA4L2	NAT8	HMGN3	TPI1	HLA.B
CLU	GPX3	ECH1	PGK1	CD24	ACMSD
TCEA1	CHCHD10	SPINK1	RNF187	TSLP	NUPR1
RPLP1	SLC3A2	FTL	BEX2	RPS16	EIF1
HSPA9	EIF2S2	RPL13A	LARP6	RPS27	REXO2
GFPT1	CEBPG	RPL30	CLGN	CCPG1	SARS
MTHFD2	SEC11C	CTC.573N18.1	RPL37A	NACA	SHMT2

T10: 146 cells; 7 programs

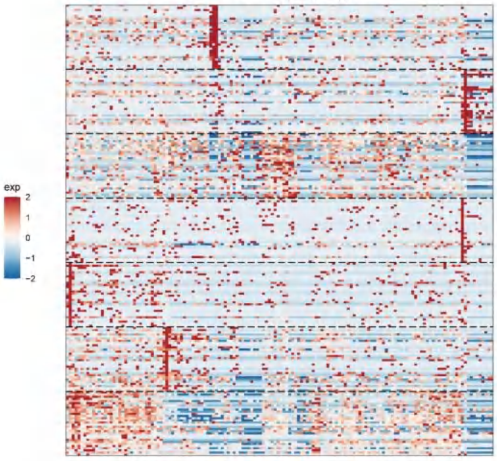

T10_1; T10_2; T10_3; T10_4; T10_5; T10_6; T10_7

MARCKSL1	BCL2A1	EBI3	LAMP3	CD40	TUBB6
TRAF1	SRGN	BIRC3	RASSF4	EPSTI1	CCR7
LITAF	NUB1	PLEK	NFKBIA	POGLUT1	SAMSN1
CD83	N4BP2L1	CKB	GSN	IRF8	MGLL
IRF1	TXN	RASSF2	CD86	MIR23AHG	SLC5A6
H4C3	SPAG4	NDUFA4L2	MT2A	PBK	PIMREG
CKS2	TUBA1C	IGFBP7	CRYAB	VIM	TUBA1B
CAV1	ANGPTL4	CDCA3	ANXA2	EGLN3	FHL2
ATP8B3	KRT8	RARRES2	FOS	CALD1	RHEB
ATF3	GAPDH	PPFIBP1	PHLDA2	ENPEP	MIF
CORO1A	ARHGDIB	SGMS1	EVL	YWHAZ	TRAF3IP3
GNAI2	CNN2	SELPLG	BIN2	RAC2	LCK
ITGAL	CXCR6	SYNE1	RCSD1	ZC3HAV1	ITGB2
VAMP4	ARL4C	THEMIS	IQGAP1	RFX7	ARFGAP1
TMSB4X	ZFP36L2	TRMT2B	TAGAP	GIMAP4	GIMAP7
FGL2	SAMHD1	FAM13A	FUBP3	SMIM14	HPS5
SLC22A5	GCLC	SCD	APBB3	KLHL24	SKIL
EFR3A	SLC39A10	MS4A6A	NUDT16	MYOF	ZBTB4
ABL2	VCL	SGK1	HLA.DRA	GRN	FAM91A1
SPRYD7	RBBP9	ZNF852	WARS1	TNKS1BP1	DIXDC1
CASZ1	RAMP1	MAVS	LARP1B	FLAD1	NDUFAF7
CTTN	COQ2	DDIT3	TOPORS	TMEM97	RASGEF1A
PSMA8	STAMBPL1	STX11	PRPF18	CNNM2	RIPK2
CGAS	BAG3	PRTFDC1	NFE2L3	BCAT1	SCNN1B
CCDC150	PMAIP1	USP25	SCPEP1	EAF2	ZNF689
IL2RA	LTA	FOXP3	C1QBP	TNFRSF4	CORO1B
EIF5A	TTLL12	MCRIP2	SOCAR	NPM1	NOP16
MYC	RAB33A	LTB	UTP11	CEP72	ZBTB32
RRP9	WDR77	LAIR2	HMGA1	PAFAH1B3	ATP5MC3
APRT	SLIRP	WDR43	IMPDH2	HACD1	SNRPG
PON2	HLA.DQA2	RAMP1.1	CTHRC1	CST7	XCL1
CMC1	PON3	HLA.DQA1	HLA.DMA	FAM174B	HLA.DRB1
CD27	HLA.DRB5	SYNGR2	PMCH	DCTPP1	ITM2A
SEC11C	DBI	NMNAT3	TAF13	VCAM1	CTSW
PPIA	XCL2	ARF5	COCH	TYMS	NAA60

T06: 3301 cells; 6 programs

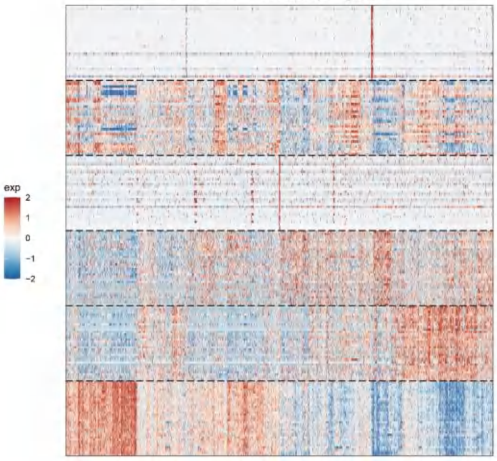

T06_1; T06_2; T06_3; T06_4; T06_5; T06_6

TOP2A	PTTG1	UBE2C	CCNB2	TPX2	CDKN3
CDCA3	CDK1	HJURP	CDC20	BIRC5	MKI67
PBK	CENPF	HMMR	GTSE1	ASPM	DLGAP5
NUF2	STMN1	HMGB3	TROAP	ANLN	SPC25
AURKB	NUSAP1	CEP55	UBE2T	TUBA1B	CENPA
RPL8	RPL7	MT.ND2	UQCRB	MT.ND5	MT.ND3
CD74	RPL12	RPL30	HLA.DQA1	FXYD2	EEF1D
HLA.DPA1	PABPC1	RARRES3	EIF3E	GNB2L1	RARRES2
B2M	MT.CO2	HLA.DRA	RPL10	HLA.A	HINT1
HLA.DPB1	RPS2	RPS20	RPLP0	RPL7A	HLA.B
TYROBP	C1QB	C1QA	SRGN	EREG	C1QC
CCL4	GPR183	MS4A6A	TNFAIP3	IL1B	CCL4L2
APOC1	NLRP3	LAPTM5	REL	PLEK	RGS1
DUSP2	FAM26F	TMSB4X	LST1	MS4A7	DUSP4
FCER1G	AIF1	PTPRC	CCR7	TCHH	CSF1R
CCL2	KDM6B	IER3	CDKN1A	PLEKHA1	YBX3
H3F3B	MIDN	PFKFB3	IGFBP3	SOD2	MIR22HG
ZFP36L1	TNFRSF12A	ODC1	MCL1	NEAT1	NAMPT
LMNA	ETS1	HMGN3	PTRF	FOSL2	CITED4
ITGA3	ANGPTL4	AHNAK	TNFRSF21	NFIL3	MAP2K3
TSPAN1	CDHR5	CES2	PDZK1IP1	CYB5A	NAT8
AMN	KRT18	SMIM24	AOC1	PCSK1N	TMEM176A
LGALS2	ECH1	TMEM176B	CXCL14	AKR7A3	BSG
KHK	SLC2A5	ACSM2B	HLA.DRB1	TM4SF5	SLC6A13
ITM2B	ACAT1	MGST1	ADIRF	HSPE1	PEBP1
RPL18	RPL13A	RPL36	RPS27	RPLP2	RPL21
RPL9	RPS16	RPL13	RPS3	RPL19	RPS18
RPS25	RPS13	RPL27A	RPL18A	RPL5	RPS5
RPL15	RPL38	RPL23A	NPM1	RPL3	RPS27A
RPS6	RPL35	RPL11	RPS3A	RPS14	RPS23

T11: 845 cells; 5 programs

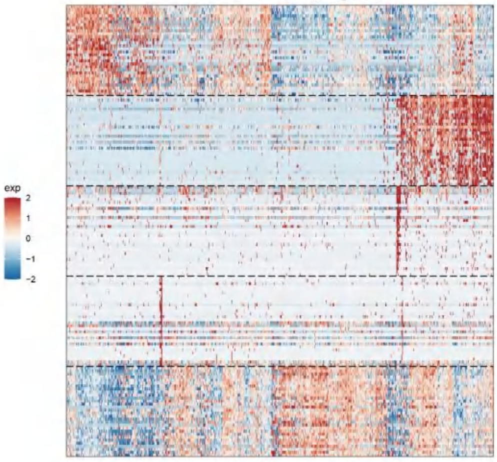

T11_1; T11_2; T11_3; T11_4; T11_5

PDZK1IP1	CYB5A	ATP5MC3	CXCL14	PCSK1N	CLTRN
KHK	UQCRQ	SMIM24	HINT1	DEFB1	TRIM54
GPD1	GAL3ST1	AZGP1	COX6A1	SLC23A3	ADIRF
SLC5A12	SLC39A5	TNFRSF12A	TSPAN1	NAT8	GPX3
GSTK1	UQCR10	SLC37A4	COX5B	CUBN	CYB5R3
CD3E	TMSB4X	SRGN	ARHGDIB	ZFP36L2	HCST
CORO1A	PTPRCAP	IL2RG	CD52	HLA.C	CD37
CD3D	HLA.E	CD69	JUNB	LSP1	BTG1
CYTIP	LCP1	LCK	CD2	PTPRC	CD53
ALOX5AP	EVL	CD7	TNFAIP3	CD8B	CD96
HLA.DRA	HLA.DPB1	HLA.DPA1	HLA.DQA1	LYZ	TYROBP
FN1	HLA.DRB1	C1QA	VSIG4	HLA.DRB5	FCER1G
MS4A6A	HLA.DQB1	S100A9	HLA.DQA2	C1QB	CD86
FCGR3A	C1QC	HLA.DMB	RNASE6	AIF1	C1orf162
SPI1	FGR	FCGR2B	CTSS	SIGLEC10	SLAMF8
ASPM	CENPF	PTTG1	CDCA3	MKI67	UBE2T
CENPE	TACC3	CDKN3	STMN1	NMU	RRM2
PHF19	CCNB1	FAM83D	H2AZ1	TUBB4B	PRC1
ARL6IP1	ANLN	MYH9	PRR11	TUBA1B	APOBEC3B
INCENP	UBE2C	CCNB2	PCLAF	LGALS1	LMNA
SQSTM1	ENO2	SARS1	PLIN2	EIF1	NPM1
VIM	CCNI	EEF1D	CNBP	EIF4A2	S100A6
PTGES3	UCHL1	RACK1	ALDOA	FTL	TCEA3
TCEA1	BEX2	PCBP2	NOP53	RBCK1	TAGLN2
U2AF1	PSAP	EPRS1	EEF2	NUPR1	TNIP1

T13: 958 cells; 4 programs

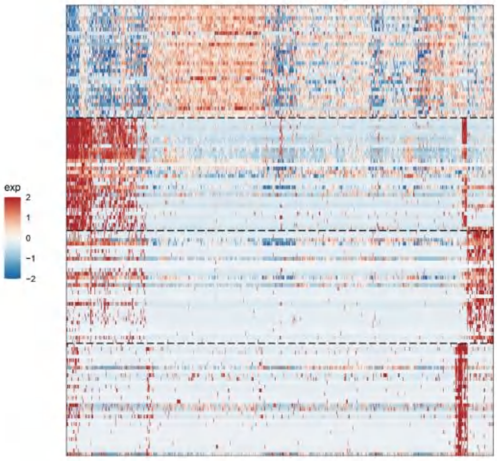

T13_1; T13_2; T13_3; T13_4

PDZK1IP1	HINT1	COX7C	ANGPTL4	PPIB	GAPDH
ATP5MC3	TRABD2B	ALDOA	MIF	DEFB1	COX5B
CD151	MGST1	PRELID1	TNFRSF12A	S100A1	COX6A1
NNMT	ALKAL2	CXCL14	TPI1	RBP5	S100A10
CA12	ADIRF	MLEC	AQP1	TMEM176B	HSPB1
C1QA	C1QB	APOE	C1QC	TYROBP	HLA.DPA1
HLA.DPB1	RNASE1	HLA.DRB1	HLA.DRA	HLA.DRB5	CST3
S100A8	GPX1	FTL	CD74	FCER1G	AIF1
TMSB4X	HLA.DQB1	GRN	MS4A6A	HLA.DQA1	LAPTM5
C1orf162	PLTP	APOC1	TREM2	LYZ	S100A4
PTPRCAP	CD3E	BTG1	TMSB4X.1	SIRPG	CD69
CST7	JUNB	SLAMF1	P2RY10	CD52	SRGN
HLA.C	LCK	ZFP36L2	DUSP2	CD2	MEI1
CD3D	ARHGDIB	KLRB1	GZMA	CD7	CYTIP
NKG7	TMIGD2	CD8A	SPNS3	CORO1A	CD27
HMMR	PTTG1	CCNB1	BUB1B	CENPF	CDC20
H2AZ1	PIMREG	UBE2S	CKS2	CDC25C	MKI67
PCLAF	BUB1	CCNB2	TRIP13	TUBB4B	ARL6IP1
ASPM	STMN1	CDCA8	TPX2	OIP5	CDCA3
TROAP	KIF20A	PRR11	FOXM1	PLK1	HMGN2

T15: 247 cells; 5 programs

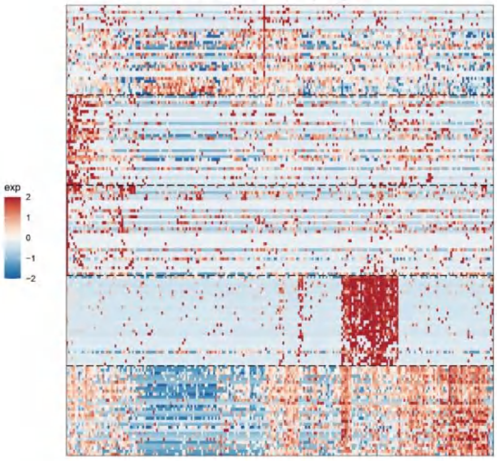

T15_1; T15_2; T15_3; T15_4; T15_5

TM4SF1	AKR1C1	AKR1B1	SERPINE2	HMOX1	AKR1C3
PRSS23	PYGL	NQO1	ATP5MC3	ATP5F1C	MAL2
TXN	ANXA2	ANXA5	WLS	SLC25A5	AHCY
G6PD	ACTG1	TUBB	COX5A	CHD1L	DERL3
NDUFA4	PRDX5	PPIA	FTL	FTH1	ATP5MF
SLC3A1	MSLN	LINC01320	NAT8	BAIAP2L2	RDH5
RNF186	METAP1D	CDHR5	PDZK1	NBPF1	FCGRT
EFNA1	TMEM176B	BBOX1	KCNQ1OT1	PCK1	ZNF639
ATP1B1	SLC23A1	RTN4	TMEM176A	CNTNAP5	CCN1
UGT2B7	STX16	APLN	KHK	SLC5A1	FAM13A
CA12	VEGFA	NDRG1	MT1X	DEPP1	TGFBR2
SERPINB9	MYEOV	PLOD2	SEMA4B	SOX4	SYCE1L
LGALS1	FCAMR	MT2A	ANGPTL4	KLF13	ITK
TNFRSF1B	GMEB1	MDC1	MGLL	TNS1	ARRDC2
TMSB10	CMTM3	SREBF2	C16orf74	F2R	IGFBP3
TMSB4X	CD3E	PTPRCAP	NKG7	PTPRC	RAC2
CST7	CD8A	CD27	CORO1A	CD3D	GZMK
HCST	GZMA	CD2	CXCR3	LAPTM5	GPSM3
LSP1	ARHGDIB	CTSW	SIT1	TRAF3IP3	RGS1
PRF1	SH3BGRL3	LCP2	LCP1	ITM2A	ITGA4
HLA.DRB1	HLA.DRB5	CD74	VCAM1	HLA.DQA1	C1S
HLA.A	SYNGR2	CD151	HLA.C	PDIA6	MAL
CFB	HLA.B	HLA.DRA	TAP1	SERPING1	CLU
IL18BP	HLA.DQA2	SERPINA1	CTSS	HLA.DQB1	C3
WARS1	SPINT2	FN1	TNFSF10	HLA.DPA1	PSAP

T12: 1318 cells; 5 programs

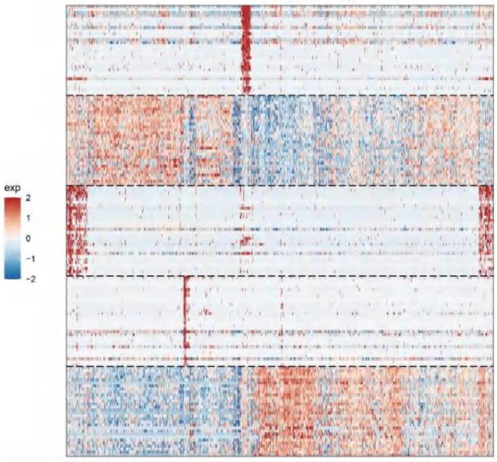

T12_1; T12_2; T12_3; T12_4; T12_5

HLA.DPA1	TYROBP	HLA.DRA	HLA.DPB1	MS4A6A	AIF1
HLA.DQA1	HLA.DRB5	FCGR2B	C1QC	HLA.DQA2	HLA.DQB1
HLA.DRB1	C1orf162	FCER1G	RNASE6	MS4A7	C1QB
IGSF6	FGL2	CTSS	LYZ	FCER1A	PLD4
LAPTM5	CLEC7A	CALHM6	C1QA	DOK2	CD1E
VIM	NPM1	DUSP1	TNIP1	CCNI	ENO2
PCBP2	TAGLN2	CNBP	EEF1A1	EEF1D	EEF2
SQSTM1	RHOB	NOL3	S100A6	UBC	CAV1
HSP90AB1	RACK1	EIF4A2	TGFBI	GLUL	EFEMP1
TCEA3	EIF3L	CPE	ANXA4	NOP53	PKM
CD69	PTPRCAP	CD3E	CD3D	CD96	XCL1
CD2	ARHGDIB	CD8B	CD52	IL2RG	CD8A
XCL2	LCK	BTG1	KLRD1	CD7	RGS1
CORO1A	EVL	SPOCK2	KLRB1	TMSB4X	CD160
HOPX	TBC1D10C	SLAMF6	CD3G	LTB	TMIGD2
PTTG1	PIMREG	CENPA	KIF20A	CENPF	CDKN3
BIRC5	DEPDC1	PLK1	CDC20	MKI67	CCNB2
CCNB1	CDCA3	CDC25C	CEP55	TOP2A	HMMR
H2AZ1	UBE2S	ASPM	TPX2	HMGB3	STMN1
KIF14	SHCBP1	NEK2	TUBA1B	SGO1	UBE2C
ATP5MC3	PRELID1	UQCRQ	PDZK1IP1	GCHFR	GPD1
CXCL14	PEBP1	CYB5A	NAT8	COX4I1	GSTK1
PEPD	COX6A1	SLC39A5	SLC5A12	COX5B	GSTA2
KHK	ANPEP	ASPDH	UQCR10	AQP1	TMEM174
GPX3	DDT	AKR1A1	ATP5PF	CLTRN	TRIM54

T16: 108 cells; 4 programs

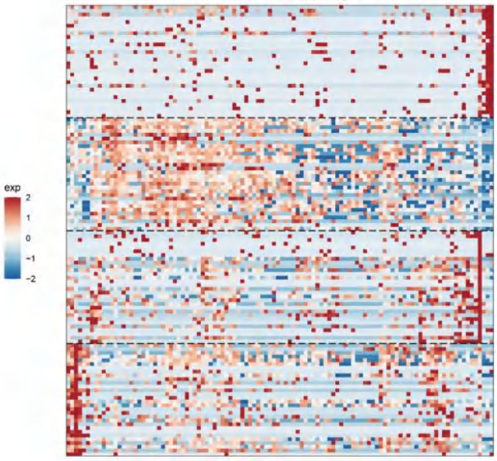

T16_1; T16_2; T16_3; T16_4

FUS	KLF6	SULT1C2	SLC25A16	PUM2	NFIB
RHPN2	BHMT2	YPEL2	ACSM2B	ADAMTS9.AS1	EXOC4
FNDC3B	CCNL1	SORBS2	LIFR	PKN2	TIPARP
RBM6	RALGAPB	DST	ACSM2A	IFRD1	TTC14
PTPRK	SRSF1	NUMB	BLNK	SEL1L3	LINC00937
FXYD2	ATP5MC3	COA3	UQCRQ	NDUFS3	NCBP2AS2
TMEM14B	ATP5MC1	COX5A	ATP5PB	COX7B	ATP5F1C
NIPSNAP2	MRPS26	C4orf3	UQCR11	UQCRB	FABP3
LDHB	CRYAB	NDUFB5	DLD	FAM162A	PRDX2
CYB5A	TMEM256	RBP5	CYC1	ALDOC	COX4I1
APOC1	APOE	C1QA	TYROBP	C1QC	C1QB
FCER1G	HLA.DPA1	SAT1	HLA.DPB1	HLA.DRA	MS4A6A
CD74	LYZ	RGS1	HLA.DRB5	CXCL13	TMSB4X
HLA.DMA	HLA.DRB1	GBP5	ZFP36L1	HLA.DQB1	SRGN
FXYD5	NPC2	PDK4	CELF2	HLA.DQA1	CTSW
UCHL1	LGALS1	ANXA2	SQSTM1	YWHAQ	S100A10
MYEOV	MCUB	S100A11	PHLDA2	CAP1	STIP1
PSMB10	CAV1	BID	PKM	S100A6	ISG20
GBP1	PSMD13	TALDO1	LMNA	SLC35A4	IDO1
WARS1	PFN1	VAMP5	VIM	S100A16	BCAP29

T27: 262 cells; 6 programs

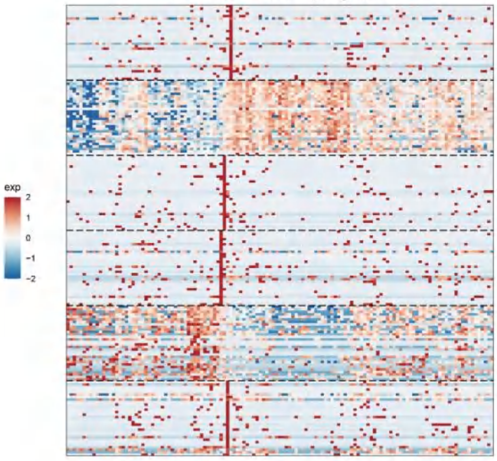

T27_1; T27_2; T27_3; T27_4; T27_5; T27_6

FBXO30	TMEM79	RP11.354K1.1	DNAJC18	C6orf203	UGT2B7
KIAA1586	BATF3	KDM1B	ALS2	BNIP1	PVRL3
ASAP3	FAM43A	RP11.359P5.1	FGA	CD55	IL1RL1
ZBTB49	LPIN1	CUL9	RP11.1C1.4	RP11.326I11.3	RP3.395M20.12
LEPROT	RP1.232P20.1	DNAJB11	CCBL2	CTC.338M12.5	ZC3H8
RPS3A	EEF1A1	RPS8	RPL11	GNB2L1	HINT1
RPS18	RPL5	RPS12	RPS23	RPL15	MARCKSL1
BTF3	RPL10A	NPM1	EEF1B2	RPL9	RPL34
RPL14	RPL32	HMGN2	RPL37	RPS27A	SRP9
RPS14	RPS7	NDUFS5	HIST1H4C	RPL24	CCDC148
LGALSL	PCDP1	MAEL	CNOT10	LRRN2	RP11.545I5.3
DIRC3	CCDC30	KCTD18	RP11.488L18.10	RP5.864K19.4	ISY1
SNX18	SPRED2	CISH	MRPS9	HOXD.AS2	ZBTB49.1
RARRES1	E2F6	C3orf38	ATAD3A	ATAD3B	LRP11
ACY1	ENAH	KDM5B	CTB.178M22.2	HS2ST1	RP11.490M8.1
SST	TRAF3IP1	RP11.145A3.1	CNOT10.1	CDK7	IL1RL1.1
CCDC138	IKZF2	CDKN1A	E2F6.1	RP11.488L18.10.1	IGIP
TMEM17	C2orf74	TUBA4A	PCYT1A	STT3B	HOXD1
TAF9	SPINK1	RND3	MAP7D1	NBPF12	MAP4K3
UBE2E2	ACOX2	EGR1	HHATL	RP11.417J8.3	AHI1
ENPEP	ATP1B1	CHPF	SLC6A3	CP	DPP4
F5	AC074289.1	LPCAT1	KCNK3	QSOX1	KIAA1244
SLC36A1	TGFA	ELK4	ADAMTSL4	CXCR4	NRP2
NBL1	MICAL1	LRRC41	NIPAL3	VEGFA	ECE1
SLC12A7	MST1	CERS2	AGRN	C6orf106	TNS1
IDH1.AS1	CAPG	KALRN	SCLY	AC079354.2	LAPTM4A
FBXO30.1	FHL2	ANKRD31	RIOK2	ABRACL	C4orf6
ZKSCAN4	WWC2.AS2	GCA	NMNAT1	DBT	ANP32E
PSORS1C1	PEX13	CYB5R4	OSGEPL1	PPP1R7	B3GALT4
EFNA4	CEP57L1	FDPS	TSPAN1	DNPH1	NAT8

T32: 1683 cells; 6 programs

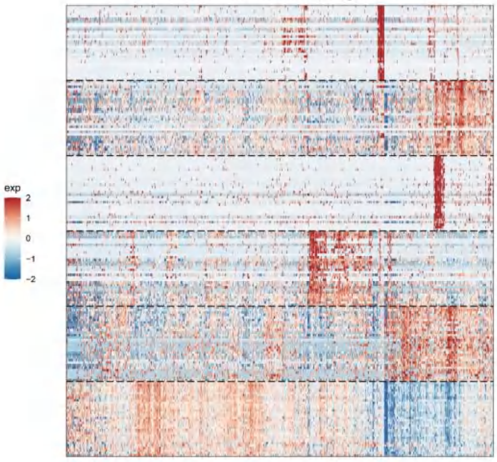

T32_1; T32_2; T32_3; T32_4; T32_5; T32_6

LAPTM5	FCER1G	C1QC	C1QA	C1QB	CD74
HLA.DPB1	LST1	STAB1	HLA.DPA1	HLA.DQA1	IL1B
HLA.DRB1	TREM2	HLA.DRA	HLA.DQB1	CD84	HLA.DQA2
HLA.DRB5	AIF1	S100A4	MNDA	C1orf162	FCGR1B
FAM198B	HLA.DMB	RGS1	FCGR3A	HCLS1	CD53
S100A11	ANXA3	SH3BGRL3	LAMC2	HMGA1	S100A16
CLIC1	WDR1	PSMD2	TAGLN2	ANXA5	ACTR3
YWHAQ	HBEGF	S100A10	UCHL1	S100A6	SFN
PRDX1	PNPLA1	PLK2	IL8	ARPC2	MAL
SSR3	CALM2	TPM3	PRELID1	HNRNPAB	YBX1
CENPA	NUF2	HJURP	CDC20	CCNA2	CENPF
KIF14	CKAP2L	SPC25	CENPE	ASPM	PTTG1
TACC3	CDCA8	SGOL2	STMN1	CCNB1	DEPDC1
H2AFZ	TRIP13	HMMR	KIF20A	NEK2	BUB1
SMC4	NCAPH	HIST1H4C	NCAPG	KIF2C	HIST1H1B
GSTA1	NAT8	LRP2	CYP4A11	CTXN3	GSTA2
C1orf210	HAO2	AGMAT	GC	FMO1	MPC2
PDZK1	KHK	APOM	ALPL	AC009014.3	DNPH1
SLC23A3	PLCH2	DAB2	AKR1A1	ACMSD	ACOT7
SMPDL3A	LAMTOR5	HMGN3	BHMT2	HSPE1	SLC3A1
AGRN	IGFBP7	FAT1	FSTL1	ITGAV	SPTBN1
CDH6	DST	VEGFA	TNFSF10	MACF1	CP
ATP13A3	PXDN	MUC1	LAMA4	CLDN1	KCNK3
LOX	SLC2A1	CRIM1	HLA.A	HSPG2	TAPBP
GOLGB1	LPCAT1	LAMC1	SLC34A2	TRABD2B	EIF4G1
RPS3A	RPS27	RPL9	RPL10A	RPS8	RPL5
EEF1A1	BTF3	RPS23	RPS14	RPS27A	RPL14
RPL32	RPL35A	CCNI	RPS18	RPL24	RPL15
RPL29	NPM1	RPL11	RPL34	GNB2L1	RPL22
CNBP	DUSP1	RPL37	RPL37A	EEF1B2	EPB41L4A.AS1

T24: 2718 cells; 7 programs

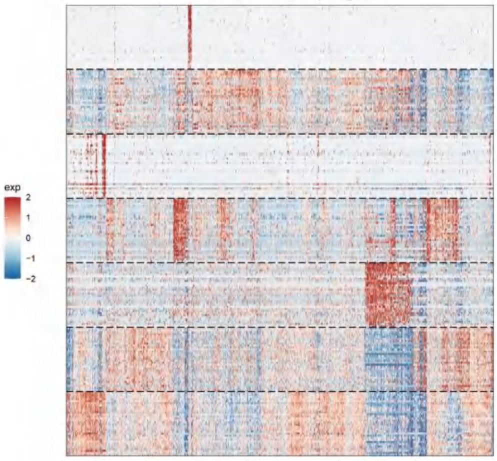

T24_1; T24_2; T24_3; T24_4; T24_5; T24_6; T24_7

CD8A	NKG7	CD3E	GZMK	GZMA	CD3D
PTPRCAP	IL2RG	FYB1	CD27	CST7	HCST
LTB	CD53	CTSW	CD2	GMFG	CORO1A
PTPRC	CD8B	CD37	ARHGDIB	TRAF3IP3	CD3G
LCP1	HAVCR2	CD52	GZMH	DOK2	CD7
EIF1	FTL	FTH1	GABARAP	PPP1R15A	PCBP2
RGS2	KDELR1	CCNI	EPB41L4A.AS1	BTG1	ZFAS1
PMM1	RHOB	VDAC2	ZFP36	NR4A2	PFDN5
IGBP1	BTG2	ATP5MC2	C1orf43	SQSTM1	EIF4A2
CCDC28A	BAG3	EIF4EBP3	ATF3	ALDOA	UBC
PTTG1	CENPF	ASPM	KIF20A	MKI67	BIRC5
HMMR	TPX2	CCNB2	CCNB1	CDC20	PLK1
NUF2	NUSAP1	PIMREG	NEK2	GTSE1	TK1
CDCA3	PBK	CDKN3	KIF14	TOP2A	STMN1
CKAP2L	H2AZ1	HJURP	UBE2T	CENPE	TROAP
OAS1	MX1	OASL	IFI35	ISG15	RSAD2
MX2	LY6E	IFITM3	IFI6	IFIT1	IFITM2
SAMD9L	UBE2L6	ISG20	PSMB9	IRF7	IFI27
IFIT3	TRIM22	TNFSF10	DDX58	PLSCR1	ETV7
XAF1	SAMD9	IFIT2	VAMP5	OAS2	GBP4
EEF1A1	TPT1	CRYL1	C3	PTMA	PLCB4
RBP5	CLU	COMMD6	P3H2	HNMT	HSP90AB1
BGN	EEF1D	SLC38A1	GLUL	RARRES2	PELO
CD74	EEF1B2	ARID5B	ITM2B	CRYAB	NOP53
NNMT	MAP3K7CL	MID1	VIM	IGFBP7	EFEMP1
DCBLD2	VCAN	CDK6	MYLK	MET	TRIO
LRP1	MACF1	NAV1	SLIT3	DCDC2	FAT1
COL21A1	MYOF	AHNAK2	DST	CD81	EPHA7
DDX17	AHNAK	GLS	PLOD2	WWTR1	MTRNR2L8
ATP2B4	ANO6	MTRNR2L12	ITGA3	MRTFA	AKT3
ACTB	CFL1	PFN1	RAN	PKM	TNFRSF12A
S100A16	EIF5A	HINT1	SLC25A5	ANXA2	ARPC2
ACTG1	PGAM1	MYL12A	CSRP1	ATP5MC3	SLC25A3
COX5A	CNN2	SYNGR2	VDAC1	RHOA	S100A11
MYL12B	SLC9A3R1	YIF1A	TUBA1B	SH3BGRL3	MYDGF

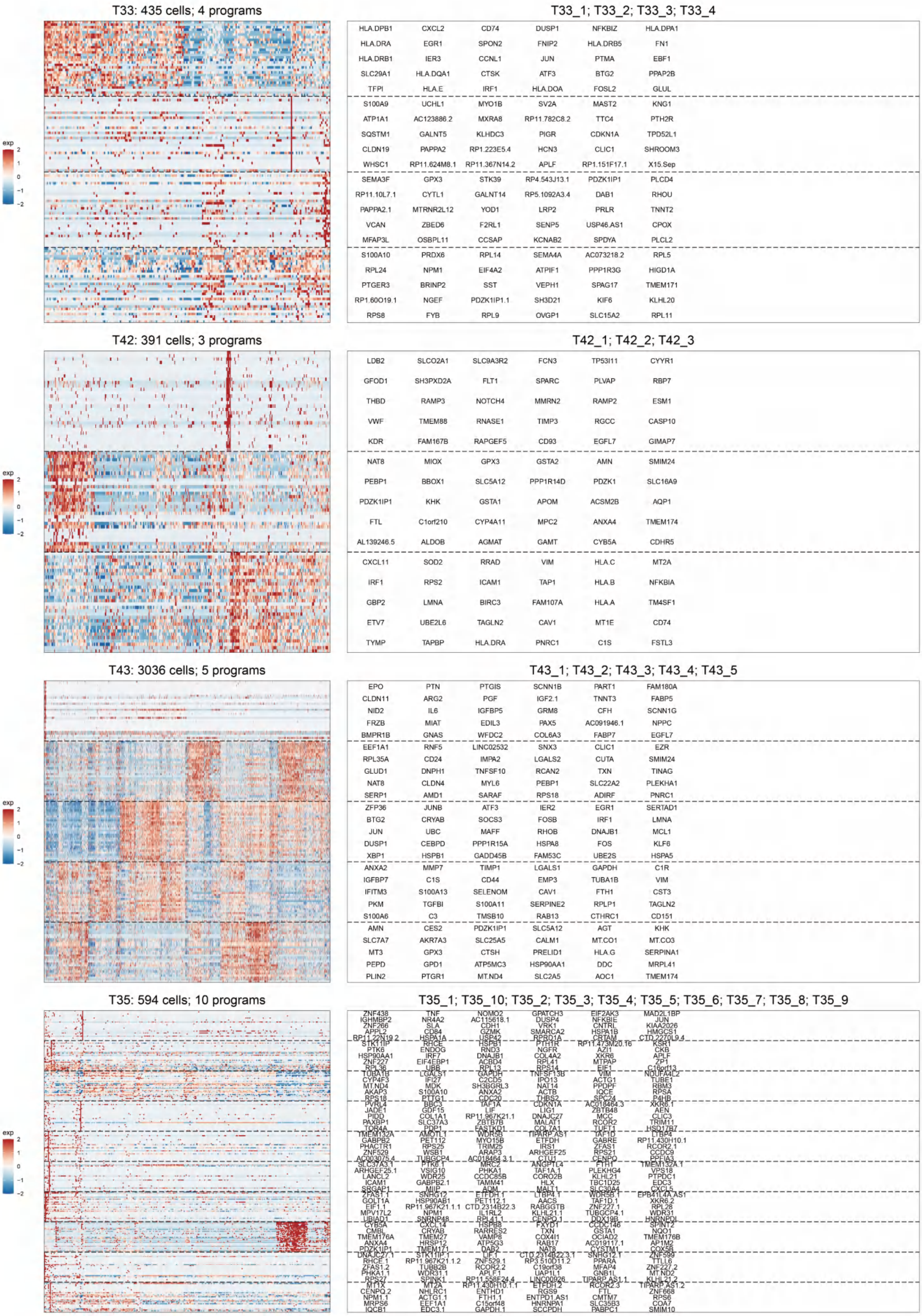

T33: 435 cells; 4 programs
exp 2 1 0 −1 −2
T33_1; T33_2; T33_3; T33_4
HLA.DPB1 CXCL2 CD74 DUSP1 NFKBIZ HLA.DPA1
HLA.DRA EGR1 SPON2 FNIP2 HLA.DRB5 FN1
HLA.DRB1 IER3 CCNL1 JUN PTMA EBF1
SLC29A1 HLA.DQA1 CTSK ATF3 BTG2 PPAP2B
TFPI HLA.E IRF1 HLA.DOA FOSL2 GLUL
S100A9 UCHL1 MYO1B SV2A MAST2 KNG1
ATP1A1 AC123886.2 MXRA8 RP11.782C8.2 TTC4 PTH2R
SQSTM1 GALNT5 KLHDC3 PIGR CDKN1A TPD52L1
CLDN19 PAPPA2 RP1.223E5.4 HCN3 CLIC1 SHROOM3
WHSC1 RP11.624M8.1 RP11.367N14.2 APLF RP11.151F17.1 X15.Sep
SEMA3F GPX3 STK39 RP4.543J13.1 PDZK1IP1 PLCD4
RP11.10L7.1 CYTL1 GALNT14 RP5.1092A3.4 DAB1 RHOU
PAPPA2.1 MTRNR2L12 YOD1 LRP2 PRLR TNNT2
VCAN ZBED6 F2RL1 SENP5 USP46.AS1 CPOX
MFAP3L OSBPL11 CCSAP KCNAB2 SPDYA PLCL2
S100A10 PRDX6 RPL14 SEMA4A AC073218.2 RPL5
RPL24 NPM1 EIF4A2 ATPIF1 PPP1R3G HIGD1A
PTGER3 BRINP2 SST VEPH1 SPAG17 TMEM171
RP1.60O19.1 NGEF PDZK1IP1.1 SH3D21 KIF6 KLHL20
RPS8 FYB RPL9 OVGP1 SLC15A2 RPL11
T42: 391 cells; 3 programs
exp 2 1 0 −1 −2
T42_1; T42_2; T42_3
LDB2 SLCO2A1 SLC9A3R2 FCN3 TP53I11 CYYR1
GFOD1 SH3PXD2A FLT1 SPARC PLVAP RBP7
THBD RAMP3 NOTCH4 MMRN2 RAMP2 ESM1
VWF TMEM88 RNASE1 TIMP3 RGCC CASP10
KDR FAM167B RAPGEF5 CD93 EGFL7 GIMAP7
NAT8 MIOX GPX3 GSTA2 AMN SMIM24
PEBP1 BBOX1 SLC5A12 PPP1R14D PDZK1 SLC16A9
PDZK1IP1 KHK GSTA1 APOM ACSM2B AQP1
FTL C1orf210 CYP4A11 MPC2 ANXA4 TMEM174
AL139246.5 ALDOB AGMAT GAMT CYB5A CDHR5
CXCL11 SOD2 RRAD VIM HLA.C MT2A
IRF1 RPS2 ICAM1 TAP1 HLA.B NFKBIA
GBP2 LMNA BIRC3 FAM107A HLA.A TM4SF1
ETV7 UBE2L6 TAGLN2 CAV1 MT1E CD74
TYMP TAPBP HLA.DRA PNRC1 C1S FSTL3
T43: 3036 cells; 5 programs
exp 2 1 0 −1 −2
T43_1; T43_2; T43_3; T43_4; T43_5
EPO PTN PTGIS SCNN1B PART1 FAM180A
CLDN11 ARG2 PGF IGF2.1 TNNT3 FABP5
NID2 IL6 IGFBP5 GRM8 CFH SCNN1G
FRZB MIAT EDIL3 PAX5 AC091946.1 NPPC
BMPR1B GNAS WFDC2 COL6A3 FABP7 EGFL7
EEF1A1 RNF5 LINC02532 SNX3 CLIC1 EZR
RPL35A CD24 IMPA2 LGALS2 CUTA SMIM24
GLUD1 DNPH1 TNFSF10 RCAN2 TXN TINAG
NAT8 CLDN4 MYL6 PEBP1 SLC22A2 PLEKHA1
SERP1 AMD1 SARAF RPS18 ADIRF PNRC1
ZFP36 JUNB ATF3 IER2 EGR1 SERTAD1
BTG2 CRYAB SOCS3 FOSB IRF1 LMNA
JUN UBC MAFF RHOB DNAJB1 MCL1
DUSP1 CEBPD PPP1R15A HSPA8 FOS KLF6
XBP1 HSPB1 GADD45B FAM53C UBE2S HSPA5
ANXA2 MMP7 TIMP1 LGALS1 GAPDH C1R
IGFBP7 C1S CD44 EMP3 TUBA1B VIM
IFITM3 S100A13 SELENOM CAV1 FTH1 CST3
PKM TGFBI S100A11 SERPINE2 RPLP1 TAGLN2
S100A6 C3 TMSB10 RAB13 CTHRC1 CD151
AMN CES2 PDZK1IP1 SLC5A12 AGT KHK
SLC7A7 AKR7A3 SLC25A5 CALM1 MT.CO1 MT.CO3
MT3 GPX3 CTSH PRELID1 HLA.G SERPINA1
PEPD GPD1 ATP5MC3 HSP90AA1 DDC MRPL41
PLIN2 PTGR1 MT.ND4 SLC2A5 AOC1 TMEM174
T35: 594 cells; 10 programs
exp 2 1 0 −1 −2
T35_1; T35_10; T35_2; T35_3; T35_4; T35_5; T35_6; T35_7; T35_8; T35_9

T45: 1902 cells; 5 programs

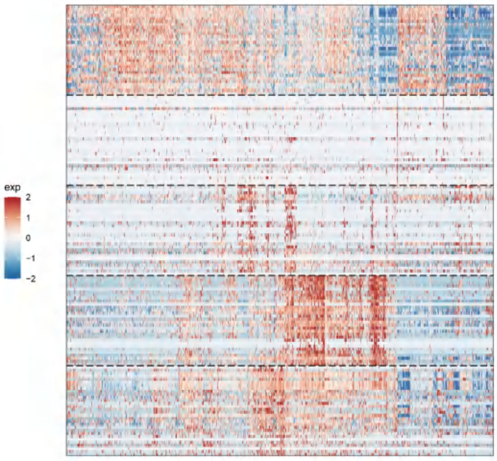

T45_1; T45_2; T45_3; T45_4; T45_5

CRYAB	ZFP36	FOSB	JUNB	DDX5	SERTAD1
UBC	MYC	CYR61	SOCS3	DNAJA1	EIF1
HSPA8	NNMT	EGR1	MAFF	ATF3	HSP90AB1
HSPB1	ACTG1	HSPA1B	GADD45B	DNAJB4	RPS27
FOS	CDKN1A	RPL21	RPL9	HSPA1A	RPS3
SFRP2	COL3A1	COL1A2	DKK3	TIMP1	CD44
COL1A1	CPE	F2R	CTGF	SERPINE1	COL6A2
TSPAN2	COL6A3	SPARC	COL8A1	TSHZ2	CFH
BGN	EFEMP1	SEMA3C	C1R	MFSD2A	IGFBP7
MT1G	LOXL2	JMY	CTHRC1	CYP1B1	CHST7
EEF1A1	GPR137B	GNG11	VIM	RPS12	IFI27L2
ANK2	HAVCR2	PTGS1	KMO	ALDH8A1	UGT3A1
ZFAND2A	CA2	LIPC	PAH	TEX41	NPTX2
GPM6A	RPL12	QPRT	S100A13	GPX3	IRF8
MOXD1	EIF4EBP1	B2M	HPGD	SERPING1	TUBA3D
CES2	AMN	KHK	ACSM2B	CYP4A11	AGT
CDHR5	PEPD	BBOX1	SLC22A6	SMIM24	TXN
GPX4	GPD1	SLC6A13	PEBP1	PDZK1IP1	APOM
CYB5A	GSTA2	SLC16A9	SLC5A12	G6PC	ASPDH
RAB11FIP3	AZGP1	MIOX	MT.CYB	ACSM2A	FBP1
AC022509.2	BIRC3	MT.CO3	MT.CO1	IGFBP3	MT.ND4
MT.CO2	MT.ND3	SDC4	ZFP36L1	IL32	WTAP
SOX9	MT.ATP6	FSTL3	SOD2	MT.CYB.1	CFLAR
TPM1	PFN1	NBL1	TYMP	ATP1B1	THBS1
IFFO2	KDM6B	SET	VXN	SLC5A3	FLNA

T47: 3197 cells; 7 programs

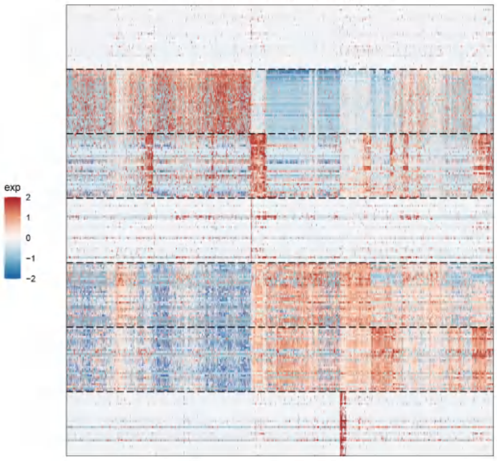

T47_1; T47_2; T47_3; T47_4; T47_5; T47_6; T47_7

DCN	MEG3	COL1A1	ITGBL1	INHBA	LUM
COL1A2	SFRP4	MXRA5	SFRP2	COL8A1	THBS2
PHACTR2	ID3	FERMT1	BGN	PRRX1	COL12A1
COL11A1	TIMP3	COLEC12	RGS16	AL356512.1	SPARC
MFAP5	COMP	RAB31	OLFML2B	LINC01521	MFAP2
NEAT1	MALAT1	N4BP2L2	VMP1	P3H2	XIST
ZBTB38	ABCC3	RHEX	BHMT2	PAG1	AL713998.1
ZBTB20	SLC25A37	SPINK13	MUC20.OT1	KLF6	DDX17
CFLAR	PDK4	DNAH11	CPD	CES4A	MBNL1
PAX2	PPP2R3A	QTRT1	VEGFA	PNISR	MAST4
JUNB	FOS	JUN	EGR1	DNAJB1	TPT1
PPP1R15A	SERTAD1	ATF3	FOSB	GADD45B	IER2
RHOB	DUSP1	AC020916.1	HSPA1B	HSPA1A	RPL6
KLF4	ZFP36	BTG2	CRYAB	INTS6	HSPA8
DNAJB4	DNAJA1	HSP90AA1	RPL21	UBC	SERPINA1
PIGR	CLIC6	LTF	MMP7	LINC00284	TMEM98
LCN2	CCL2	SERPINA1.1	SAA2	ZNF667.AS1	CXCL3
KCNJ15	CXCL2	CXCL1	RIPOR3	CLDN7	PHACTR2.1
IGFBP7	PROM1	VCAM1	PCSK1N	PACSIN3	IER3
CYR61	SFRP2.1	BGN.1	SAA1	MAP7	ANK3
GSTA1	GAPDH	SPP1	RPS2	LGALS3	HP
CRABP1	GSTA2	APCS	ARG2	LDHB	RPL10A
RPL7A	CD24	RACK1	PRDX1	RPS4X	RPS14
AGT	RPL3	HINT1	AKR1B1	NNMT	RPS3
CLU	ATP6V0E1	HNRNPA1	BTF3	FGG	SLC25A6
FTH1	EIF1	ZFAS1	VIM	RPS27	PNRC1
RPS21	RPS13	RPS12	RPL18A	HLA.C	HLA.DQA1
BTG1	CCNI	FTL	CD74	RPL30	RPL19
RPL41	RPS27A	RPL27	PSMA7	RPL9	SQSTM1
RPS29	RPL10	HLA.B	RPLP1	HLA.DRA	RPS26
NUF2	DEPDC1	CDKN3	CCNB1	NEK2	PTTG1
CKAP2L	NDC80	NCAPH	PIMREG	SPC25	HMMR
AURKA	STMN1	BIRC5	TOP2A	H2AFZ	CCNB2
SGO1	CKS2	HJURP	UBE2C	TUBA1B	PBK
CENPA	GTSE1	ASPM	BUB1B	CDCA8	ASF1B

T44: 1562 cells; 5 programs

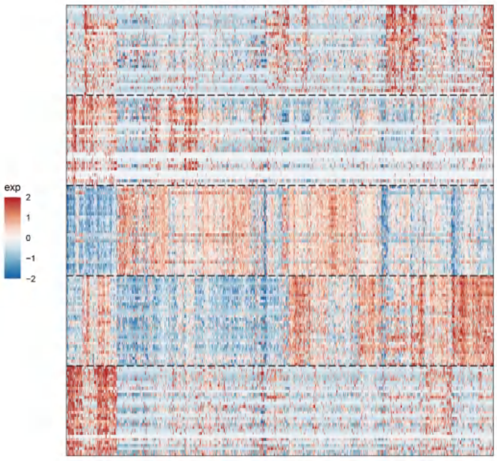

T44_1; T44_2; T44_3; T44_4; T44_5

MT2A	C11orf96	CP	MT1X	CEBPB	PNRC1
PGF	SOD2	TGFBI	NPTX2	RPS12	CDKN1A
DUSP1	IGFBP7	CTHRC1	TMSB10	RHOB	MT1F
YBX3	CXCR4	RARRES2	ELL2	STC1	NAMPT
C1R	ZFP36L1	TMEM173	BACE2	KLF4	MT1E
UBD	RARRES3	CD74	IL32	PSME2	HLA.DRB1
HLA.B	PFN1	B2M	HLA.DQA1	CXCL11	LAP3
HLA.A	GBP4	HLA.C	HLA.DRA	TAP1	PSMB9
TYMP	G0S2	LTB	ISG20	HLA.DPA1	PSME1
HLA.DPB1	SLC26A9	CTSS	GC	S100A11	AC022509.2
VIM	RPL13A	MSLN	RPS27A	RPL10	RPS3
RPL9	RPS18	RPL37A	RPL13	RPSA	RPS5
RPL11	RPL23A	RPS15	RPS24	FAM120AOS	RPL7A
PNCK	RPL41	RPL35	RPL27A	RPL18	RPS2
RPL14	BST2	RPS8	RPL18A	FAU	RPS28
HSPA8	DNAJB1	BAG3	DNAJA1	HSPA1B	HSP90AA1
SERPINH1	HSPA1A	HSPB1	FOSB	HSPD1	HSPH1
ZFAND2A	CRYAB	EGR1	HSPE1	HSPA5	PPP1R15A
DNAJB4	ATF3	ZFP36	MAFF	UBB	FOS
JUNB	BTG2	ACTG1	HSP90AB1	ACTB	MCL1
NAT8	ACSM2B	LGALS2	KHK	HMGN3	BBOX1
AMN	CYB5A	APOM	UQCRQ	FABP7	SMIM24
AQP1	GSTA1	BSG	MT.CO2	AGXT2	SLC22A2
MT.CO1	OCIAD2	SLC6A13	GSTA2	CUBN	RDH12
COX7C	MIOX	CXCL14	GSTP1	DAB2	PDZK1IP1

T46: 1244 cells; 5 programs

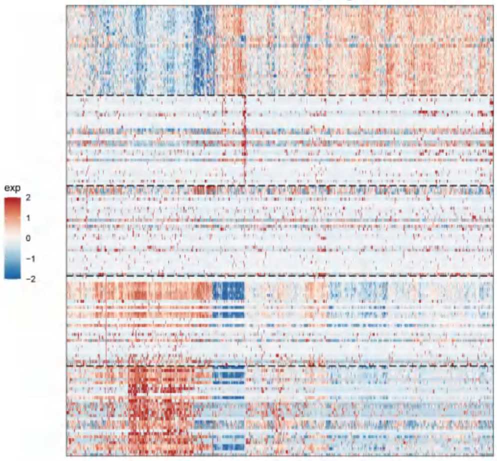

T46_1; T46_2; T46_3; T46_4; T46_5

SERTAD1	RPS3A	RPS3	FTH1	RPL7	RPL21
RPL11	RPS27A	RPL9	RPL8	ZFP36	CRYAB
EIF1	MYC	RPS4X	RPL23A	RPS5	RPL18
RPL13A	RPL41	RPL18A	RPL10	RPL5	JUNB
RPS27	RPS14	RPL3	RPS6	RPL13	RPL30
SFRP2	MMP7	COL1A1	COL6A3	COL1A2	TGFBI
PHLDA1	COL6A2	DKK3	TMEM100	CTHRC1	MT2A
ANXA2	LOXL2	BACE2	IGFBP7	MT1E	LBH
MYL9	PHLDA3	PPIC	LGALS1	FHL3	TPST2
LOXL1	PDLIM7	BGN	FSCN1	SPARC	ARHGEF2
IGLC3	IGHG1	IGHG3	SH3BP5	MZB1	CELF2
ZP1	ITGB7	IGHGP	DERL3	CCND2	CYBA
ARHGEF3	SPP1	SRGN	GJB2	FKBP11	CCL20
P2RX5	PNOC	RECQL	BIRC3	CST6	SMAP2
MEI1	IGHG2	QPCT	IGF1	TSPAN15	FSTL3
PTGS1	AC133644.2	MT.ND4	MT.ATP6	MT.ND1	MT.CO3
MT.CO2	MT.CO1	C3	RILPL1	MT.ND3	PCLAF
MT.CYB	MT.ND2	PCSK1N	CUL9	MT.ND5	NKX3.1
LRRC8D	SLC6A3	C1QL1	VEGFA	LDLRAD3	ADGRG6
C16orf74	ZNF516	PHKA2	ALKAL2	MT.ND4L	SCD
MT.CYB.1	AMN	MT.CO1.1	MT.CO3.1	KHK	MT.CO2.1
CYP4A11	CDHR5	AQP1	SLC22A6	MT.ND4.1	APOM
SMIM24	TXN	NAT8	CES2	ACSM2B	AZGP1
CYB5A	GPX4	PDZK1IP1	TM4SF5	CUBN	MT.ND5.1
MIOX	SLC6A13	MT.ND2.1	MT.ND3.1	TSPAN1	KRT18

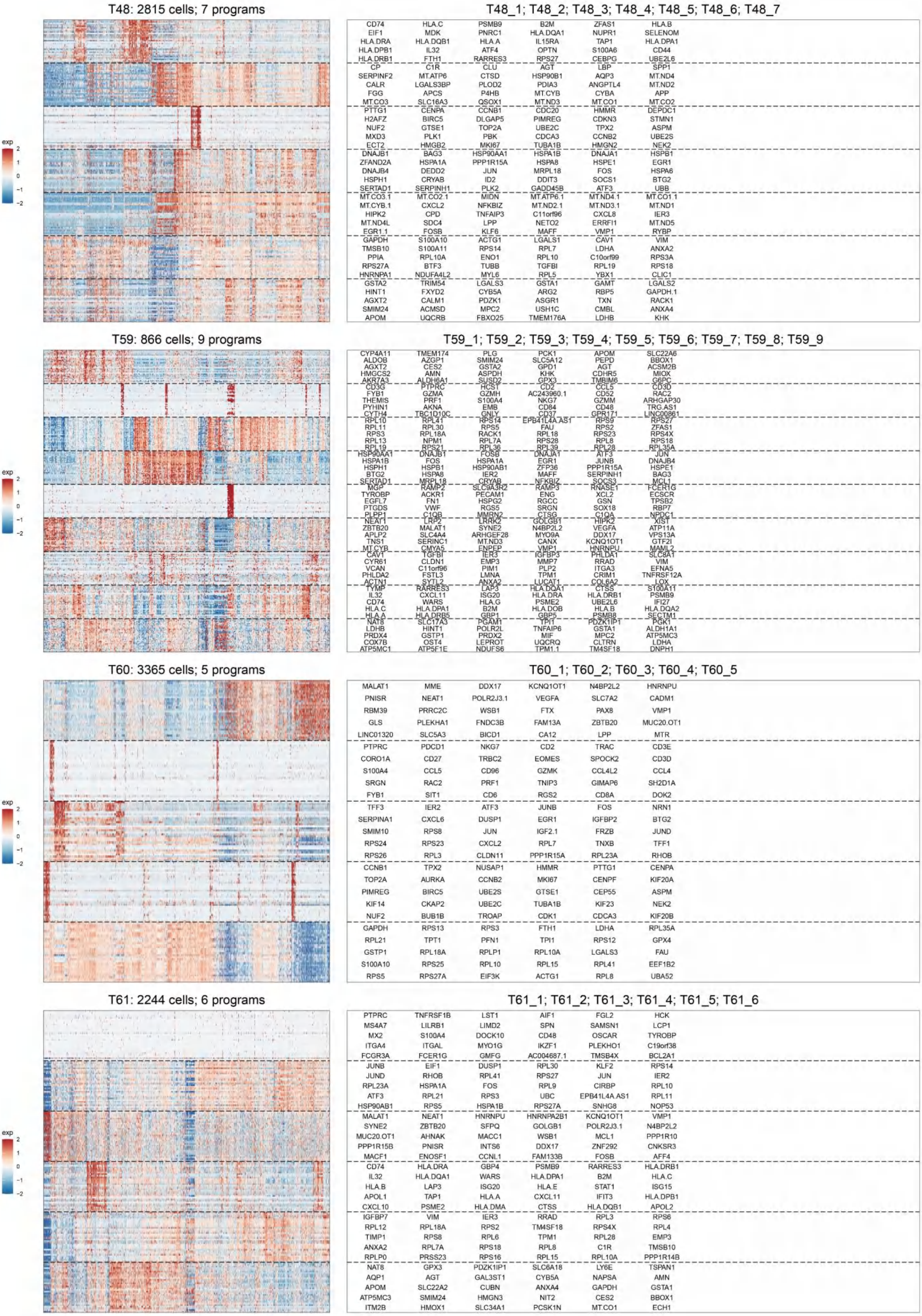
T48: 2815 cells; 7 programs
exp
2
1
0
−1
−2
T48_1; T48_2; T48_3; T48_4; T48_5; T48_6; T48_7
CD74 HLA.C PSMB9 B2M ZFAS1 HLA.B
EIF1 MDK PNRC1 HLA.DQA1 NUPR1 SELENOM
HLA.DRA HLA.DQB1 HLA.A IL15RA TAP1 HLA.DPA1
HLA.DPB1 IL32 ATF4 OPTN S100A6 CD44
HLA.DRB1 FTH1 RARRES3 RPS27 CEBPG UBE2L6
CP C1R CLU AGT LBP SPP1
SERPINF2 MT.ATP6 CTSD HSP90B1 AQP3 MT.ND4
CALR LGALS3BP PLOD2 PDIA3 ANGPTL4 MT.ND2
FGG APCS P4HB MT.CYB CYBA APP
MT.CO3 SLC16A3 QSOX1 MT.ND3 MT.CO1 MT.CO2
PTTG1 CENPA CCNB1 CDC20 HMMR DEPDC1
H2AFZ BIRC5 DLGAP5 PIMREG CDKN3 STMN1
NUF2 GTSE1 TOP2A UBE2C TPX2 ASPM
MXD3 PLK1 PBK CDCA3 CCNB2 UBE2S
ECT2 HMGB2 MKI67 TUBA1B HMGN2 NEK2
DNAJB1 BAG3 HSP90AA1 HSPA1B DNAJA1 HSPB1
ZFAND2A HSPA1A PPP1R15A HSPA8 HSPE1 EGR1
DNAJB4 DEDD2 JUN MRPL18 FOS HSPA6
HSPH1 CRYAB ID2 DDIT3 SOCS1 BTG2
SERTAD1 SERPINH1 PLK2 GADD45B ATF3 UBB
MT.CO3.1 MT.CO2.1 MIDN MT.ATP6.1 MT.ND4.1 MT.CO1.1
MT.CYB.1 CXCL2 NFKBIZ MT.ND2.1 MT.ND3.1 MT.ND1
HIPK2 CPD TNFAIP3 C11orf96 CXCL8 IER3
MT.ND4L SDC4 LPP NETO2 ERRFI1 MT.ND5
EGR1.1 FOSB KLF6 MAFF VMP1 RYBP
GAPDH S100A10 ACTG1 LGALS1 CAV1 VIM
TMSB10 S100A11 RPS14 RPL7 LDHA ANXA2
PPIA RPL10A ENO1 RPL10 C10orf99 RPS3A
RPS27A BTF3 TUBB TGFBI RPL19 RPS18
HNRNPA1 NDUFA4L2 MYL6 RPL5 YBX1 CLIC1
GSTA2 TRIM54 LGALS3 GSTA1 GAMT LGALS2
HINT1 FXYD2 CYB5A ARG2 RBP5 GAPDH.1
AGXT2 CALM1 PDZK1 ASGR1 TXN RACK1
SMIM24 ACMSD MPC2 USH1C CMBL ANXA4
APOM UQCRB FBXO25 TMEM176A LDHB KHK
T59: 866 cells; 9 programs
exp
2
1
0
−1
−2
T59_1; T59_2; T59_3; T59_4; T59_5; T59_6; T59_7; T59_8; T59_9
CYP4A11 TMEM174 PLG PCK1 APOM SLC22A6
ALDOB AZGP1 SMIM24 SLC5A12 PEPD BBOX1
AGXT2 CES2 GSTA2 GPD1 AGT ACSM2B
HMGCS2 AMN ASPDH KHK CDHR5 MIOX
AKR7A3 ALDH6A1 SUSD2 GPX3 TMBIM6 G6PC
CD3G PTPRC HCST CD2 CCL5 CD3D
FYB1 GZMA GZMH AC243960.1 CD52 RAC2
THEMIS PRF1 S100A4 NKG7 GZMM ARHGAP30
PYHIN1 AKNA EMB CD84 CD48 TRG.AS1
CYTH4 TBC1D10C GNLY CD37 GPR171 LINC00861
RPL10 RPL41 RPS14 EPB41L4A.AS1 RPS9 RPS27
RPL11 RPL30 RPS5 FAU RPS2 ZFAS1
RPS3 RPL18A RACK1 RPL18 RPS23 RPS4X
RPL13 NPM1 RPL7A RPS28 RPL8 RPS18
RPL19 RPS21 RPL36 RPL39 RPL28 RPL35A
HSP90AA1 DNAJB1 FOSB DNAJA1 ATF3 JUN
HSPA1B FOS HSPA1A EGR1 JUNB DNAJB4
HSPH1 HSPB1 HSP90AB1 ZFP36 PPP1R15A HSPE1
BTG2 HSPA8 IER2 MAFF SERPINH1 BAG3
SERTAD1 MRPL18 CRYAB NFKBIZ SOCS3 MCL1
MGP RAMP2 SLC9A3R2 RAMP3 RNASE1 FCER1G
TYROBP ACKR1 PECAM1 ENG XCL2 ECSCR
EGFL7 FN1 HSPG2 RGCC GSN TPSB2
PTGDS VWF RGS5 SRGN SOX18 RBP7
PLPP1 C1QB MMRN2 CTSG C1QA NPDC1
NEAT1 LRP2 LRRK2 GOLGB1 HIPK2 XIST
ZBTB20 MALAT1 SYNE2 N4BP2L2 VEGFA ATP11A
APLP2 SLC4A4 ARHGEF28 MYO9A DDX17 VPS13A
TNS1 SERINC1 MT.ND3 CANX KCNQ1OT1 GTF2I
MT.CYB CMYA5 ENPEP VMP1 HNRNPU MAML2
CAV1 TGFBI IER3 IGFBP3 PHLDA1 SLC8A1
CYR61 CLDN1 EMP3 MMP7 RRAD VIM
VCAN C11orf96 PIM1 PLP2 ITGA3 EFNA5
PHLDA2 FSTL3 LMNA TPM1 CRIM1 TNFRSF12A
ACTN1 SYTL2 ANXA2 LUCAT1 COL6A2 LOX
TYMP RARRES3 LAP3 HLA.DQA1 CTSS S100A11
IL32 CXCL11 ISG20 HLA.DRA HLA.DRB1 PSMB9
CD74 WARS HLA.G PSME2 UBE2L6 IFI27
HLA.C HLA.DPA1 B2M HLA.DOB HLA.B HLA.DQA2
HLA.A HLA.DRB5 GBP1 GBP5 PSMB8 SECTM1
NAT8 SLC17A3 PGAM1 TPI1 PDZK1IP1 PGK1
LDHB HINT1 POLR2L TNFAIP6 GSTA1 ALDH1A1
PRDX4 GSTP1 PRDX2 MIF MPC2 ATP5MC3
COX7B OST4 LEPROT UQCRQ CLTRN LDHA
ATP5MC1 ATP5F1E NDUFS6 TPM1.1 TM4SF18 DNPH1
T60: 3365 cells; 5 programs
exp
2
1
0
−1
−2
T60_1; T60_2; T60_3; T60_4; T60_5
MALAT1 MME DDX17 KCNQ1OT1 N4BP2L2 HNRNPU
PNISR NEAT1 POLR2J3.1 VEGFA SLC7A2 CADM1
RBM39 PRRC2C WSB1 FTX PAX8 VMP1
GLS PLEKHA1 FNDC3B FAM13A ZBTB20 MUC20.OT1
LINC01320 SLC5A3 BICD1 CA12 LPP MTR
PTPRC PDCD1 NKG7 CD2 TRAC CD3E
CORO1A CD27 TRBC2 EOMES SPOCK2 CD3D
S100A4 CCL5 CD96 GZMK CCL4L2 CCL4
SRGN RAC2 PRF1 TNIP3 GIMAP6 SH2D1A
FYB1 SIT1 CD6 RGS2 CD8A DOK2
TFF3 IER2 ATF3 JUNB FOS NRN1
SERPINA1 CXCL6 DUSP1 EGR1 IGFBP2 BTG2
SMIM10 RPS8 JUN IGF2.1 FRZB JUND
RPS24 RPS23 CXCL2 RPL7 TNXB TFF1
RPS26 RPL3 CLDN11 PPP1R15A RPL23A RHOB
CCNB1 TPX2 NUSAP1 HMMR PTTG1 CENPA
TOP2A AURKA CCNB2 MKI67 CENPF KIF20A
PIMREG BIRC5 UBE2S GTSE1 CEP55 ASPM
KIF14 CKAP2 UBE2C TUBA1B KIF23 NEK2
NUF2 BUB1B TROAP CDK1 CDCA3 KIF20B
GAPDH RPS13 RPS3 FTH1 LDHA RPL35A
RPL21 TPT1 PFN1 TPI1 RPS12 GPX4
GSTP1 RPL18A RPLP1 RPL10A LGALS3 FAU
S100A10 RPS25 RPL10 RPL15 RPL41 EEF1B2
RPS5 RPS27A EIF3K ACTG1 RPL8 UBA52
T61: 2244 cells; 6 programs
exp
2
1
0
−1
−2
T61_1; T61_2; T61_3; T61_4; T61_5; T61_6
PTPRC TNFRSF1B LST1 AIF1 FGL2 HCK
MS4A7 LILRB1 LIMD2 SPN SAMSN1 LCP1
MX2 S100A4 DOCK10 CD48 OSCAR TYROBP
ITGA4 ITGAL MYO1G IKZF1 PLEKHO1 C19orf38
FCGR3A FCER1G GMFG AC004687.1 TMSB4X BCL2A1
JUNB EIF1 DUSP1 RPL30 KLF2 RPS14
JUND RHOB RPL41 RPS27 JUN IER2
RPL23A HSPA1A FOS RPL9 CIRBP RPL10
ATF3 RPL21 RPS3 UBC EPB41L4A.AS1 RPL11
HSP90AB1 RPS5 HSPA1B RPS27A SNHG8 NOP53
MALAT1 NEAT1 HNRNPU HNRNPA2B1 KCNQ1OT1 VMP1
SYNE2 ZBTB20 SFPQ GOLGB1 POLR2J3.1 N4BP2L2
MUC20.OT1 AHNAK MACC1 WSB1 MCL1 PPP1R10
PPP1R15B PNISR INTS6 DDX17 ZNF292 CNKSR3
MACF1 ENOSF1 CCNL1 FAM133B FOSB AFF4
CD74 HLA.DRA GBP4 PSMB9 RARRES3 HLA.DRB1
IL32 HLA.DQA1 WARS HLA.DPA1 B2M HLA.C
HLA.B LAP3 ISG20 HLA.E STAT1 ISG15
APOL1 TAP1 HLA.A CXCL11 IFIT3 HLA.DPB1
CXCL10 PSME2 HLA.DMA CTSS HLA.DQB1 APOL2
IGFBP7 VIM IER3 RRAD RPL3 RPS6
RPL12 RPL18A RPS2 TM4SF18 RPS4X RPL4
TIMP1 RPS8 RPL6 TPM1 RPL28 EMP3
ANXA2 RPL7A RPS18 RPL8 C1R TMSB10
RPLP0 PRSS23 RPS16 RPL15 RPL10A PPP1R14B
NAT8 GPX3 PDZK1IP1 SLC6A18 LY6E TSPAN1
AQP1 AGT GAL3ST1 CYB5A NAPSA AMN
APOM SLC22A2 CUBN ANXA4 GAPDH GSTA1
ATP5MC3 SMIM24 HMGN3 NIT2 CES2 BBOX1
ITM2B HMOX1 SLC34A1 PCSK1N MT.CO1 ECH1

T62: 508 cells; 4 programs

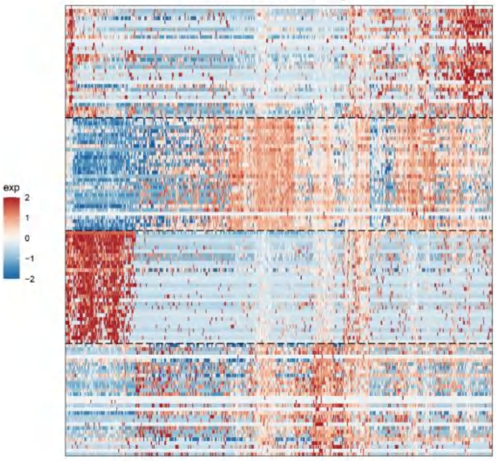

T62_1; T62_2; T62_3; T62_4

SELPLG	HSPA1B	JUNB	B2M	CD69	PTPRC
HLA.C	CD52	HSPA1A	SYNGR1	IL7R	CXCR4
DUSP2	DNAJB1	HSP90AA1	NFKBIA	HLA.B	GZMA
S100A4	CCL5	CD247	TMSB4X	BTG1	NKG7
ZFP36L2	GATA3	RPS29	HSPA8	JUN	HSPA6
FTH1	RPL41	HINT1	TPI1	RPL10	CD63
RPS12	CRYAB	GSTP1	RPL26	RPS14	RPL23A
RPS5	RACK1	NACA	BRI3	RPL35A	CYB5A
MGST1	TMEM176B	ATP5MC2	TOMM7	TMEM176A	RPS15
ZFAS1	AC078883.3	NNMT	RPS15A	RPS7	RPL13
MALAT1	NEAT1	POLR2J3.1	VMP1	FUS	PLAGL1
HNRNPA2B1	KLF6	WSB1	FRMD4A	PDK4	MUC20.OT1
KHDC4	FTX	LRRC41	NUTM2B.AS1	KCNQ1OT1	ABCC3
NOTCH2NL	SLC16A12	MCL1	SPG7	SLC25A37	LINC01320
ZNF83	ACADL	ERRFI1	PREX2	RHEX	ARHGEF10
MT.CO2	C3	LGALS3BP	ZNF490	MT.CO1	IGFBP5
HSP90B1	C1QL1	MT.CYB	COL1A2	CXCL14	IGFBP3
PTTG1IP	P4HB	ZNF497	C17orf51	SERPING1	AC010331.1
S100A11	GAL3ST1	AOC1	RPTOR	PKM	SLC6A8
APLP2	MT.ND4	CHPF	TGM2	ZNF362	FGB

T63: 1352 cells; 8 programs

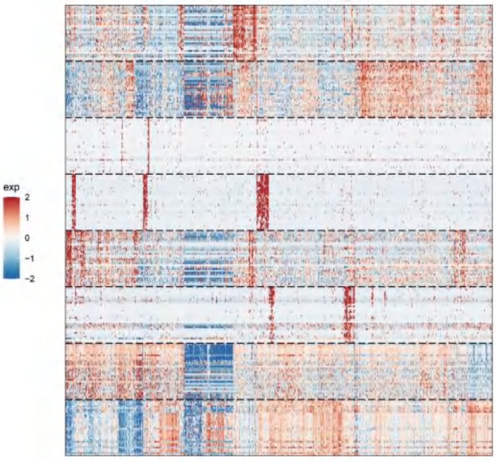

T63_1; T63_2; T63_3; T63_4; T63_5; T63_6; T63_7; T63_8

RARRES3	HLA.DRA	PSMB9	HLA.C	CD74	CXCL10
B2M	HLA.DRB1	LAP3	HLA.E	HLA.B	CXCL11
ISG20	PSME2	GBP4	TAP1	ISG15	IL32
HLA.F	HLA.DRB5	PSMB8	UBE2L6	WARS	HLA.DQA1
HLA.A	HAPLN3	VAMP5	PSME1	CXCL9	IFI27
NAT8	GAL3ST1	SLC6A18	SLC17A3	AMN	PDZK1IP1
GSTP1	GAPDH	FOLR1	SLC22A18	PRDX4	SMIM24
BSG	CYB5A	PGAM1	THY1	TPI1	AKR1B1
ATP5MC3	DNPH1	GPX4	PRELID1	COX6A1	CYC1
FCGRT	ANXA4	NAPSA	PRDX2	ATP5F1B	APOM
KDR	RGCC	TMEM204	ESM1	CLEC14A	ADGRF5
TP53I11	PDGFD	PLVAP	CDH5	CDH13	UNC5B
RFLNB	FLT1	ADGRL4	A2M	LRRC32	RHOJ
PODXL	FLT4	RAMP2	S1PR1	PLPP1	ROBO4
F2RL3	ANGPT2	LDB2	JAM3	HSPG2	APOLD1
CORO1A	CCL5	GZMA	NKG7	CD3D	CD3G
FYB1	CST7	CD3E	PTPRC	TRAC	RAC2
LCK	CD2	HCST	IKZF1	S100A4	SLA
CD37	TRBC2	SRGN	CD48	CD53	GZMK
LIMD2	CD247	CTSW	CD8A	RUNX3	CYTIP
TGFBI	ITIH5	IGFBP5	CPE	IGFBP3	PGF
MUC1	B4GALNT1	AHNAK2	C3	CAV1	NEFL
BGN	COL1A2	C5orf46	FLNA	ANGPTL4	MXRA7
COL6A3	NDUFA4L2	CST3	PMEPA1	COL23A1	DEGS1
C1R	CD151	CTSZ	SERPING1	MXRA8	EMP3
TF	INHA	FGA	FGB	FGG	EPO
S100A1	TSC22D4	BACE2	ASGR2	COLEC11	ADRA2A
VNN2	TTR	F2	LAD1	NCAM1	CLDN11
ASGR1	TFF3	WFDC2	CLU	CXCL2	AC002480.2
TPST2	TSPAN8	MAL	RPS7	SERPIND1	CFH
MALAT1	MT.ND2	MT.ND1	MT.CO3	MT.ATP6	MT.ND3
NEAT1	MT.ND4	MT.ND5	VEGFA	DDX17	SYNE2
MT.CO1	AKAP9	PNISR	HNRNPU	KCNQ1OT1	SORBS2
NCL	FAT1	MT.CYB	MT.ND6	ATP11A	POLR2J3.1
TNS1	PKHD1	ZBTB20	LINC01320	ZNF395	PRRC2C
FOS	EIF1	RPL10	JUND	DUSP1	JUNB
ZFP36	RPL41	RPS5	RPL8	RPS3	RPS14
RPL11	RPL30	RPL10A	RHOB	RPS8	RPS13
RPS3A	RPL18A	RPL18	RPS2	IER2	RPS18
RPL13	ATF3	RPL7A	RPL21	EGR1	CEBPD

T64: 322 cells; 4 programs

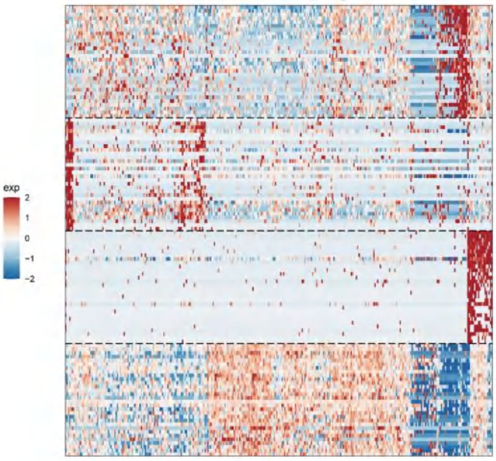

T64_1; T64_2; T64_3; T64_4

MALAT1	ZBTB20	VMP1	CCNL1	MCL1	N4BP2L2
MUC20.OT1	GOLGB1	VEGFA	DNAH11	NFKBIZ	RBM33
FKBP5	ITPRIPL2	FOSB	PNISR	RBM39	LINC01320
PLEKHH2	WDR60	TRA2A	ZBTB16	DDX3X	CHORDC1
CREBRF	WEE1	NEAT1	JMJD1C	GCC2	STAT3
CCL20	C1S	MT3	TMSB10	MT1H	LINC01638
C1R	BICDL2	CAV1	IL1R2	MT1G	EIF4EBP1
CXCL8	MT1X	GPNMB	IGFBP7	MMP7	PRSS23
C3	SYTL2	AEBP1	C2	ANXA2	LOX
CEBPB	LGALS1	CP	PHLDA1	TGFBI	EFEMP1
NKG7	CCL5	CST7	CD37	GZMA	PTPRC
CD48	TMSB4X	SRGN	GZMH	TRBC2	CD52
CYTIP	RUNX3	RAC2	TBC1D10C	CD7	SKAP1
PRF1	S100A4	TRDC	HOPX	LCK	LIMD2
KLRB1	GPSM3	CD3D	KLRD1	SLAMF6	SPN
TMEM176A	TMEM176B	TECR	LGALS2	CYB5A	PEBP1
ADIRF	FTH1	BBOX1	GAMT	IMPA2	HLA.B
ATP5F1D	TMEM37	AMN	MT.CO1	PPIA	SERPINA1
OCIAD2	PDZK1	SLC6A13	DUSP1	ACY3	CALM3
ADI1	ACO2	HPN	NDUFA2	GPX4	CLU

T65: 1899 cells; 4 programs

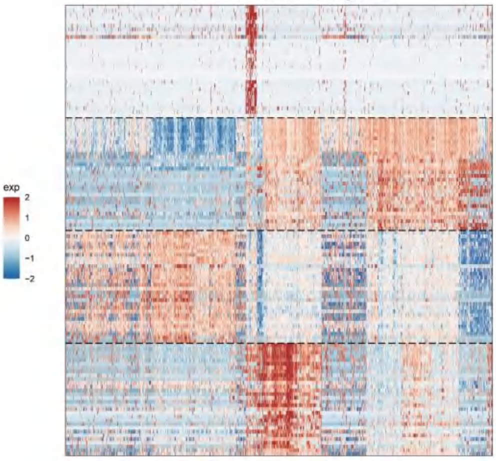

T65_1; T65_2; T65_3; T65_4

GZMA	NKG7	PTPRC	CORO1A	DOK2	CCL5
S100A4	CD52	TMSB4X	GZMM	GIMAP7	GZMH
PTPN7	GATA3	TBC1D10C	PRF1	TRBC1	SASH3
GRAP2	CCR5	HCST	CTSW	TRAC	TRBC2
CD3G	CD3E	CD2	DUSP2	CD69	SAMD3
MT.CYB	MT.ND4	MT.ATP6	MT.CO3	MT.ND1	MT.CO1
MT.ND2	MT.CO2	MT.ND3	MT.ND5	ATP1B1	VCAN
VEGFA	MALAT1	CA12	LINC01320	EDIL3	MTRNR2L12
SLC38A1	N4BP2L2	ATP11A	SPP1	TFPI	BICD1
APP	ITM2B	MT.ND4L	VCAM1	SYNE2	MYO9A
FTH1	CYB5A	GAPDH	HINT1	PEBP1	RPLP1
MYL6	RPL41	TPT1	LGALS4	SERF2	PPIA
S100A10	GSTP1	MYL12B	RPS28	VAMP8	TXN
CCDC146	CHCHD2	S100A11	TMSB10	ADIRF	BRI3
RPS15	FTL	TPI1	GPX4	RBP5	PRDX5
FOS	JUN	IER2	DNAJB1	HSPA1A	EGR1
JUNB	JUND	ATF3	PPP1R15A	UBC	CRYAB
DUSP1	HSPA1B	ZFP36	HSPA8	FOSB	HSPB1
C11orf96	SERTAD1	SERPINH1	TUBA1A	SOCS3	HSP90AA1
CYR61	HSP90AB1	BAG3	DNAJA1	BTG1	H3F3B

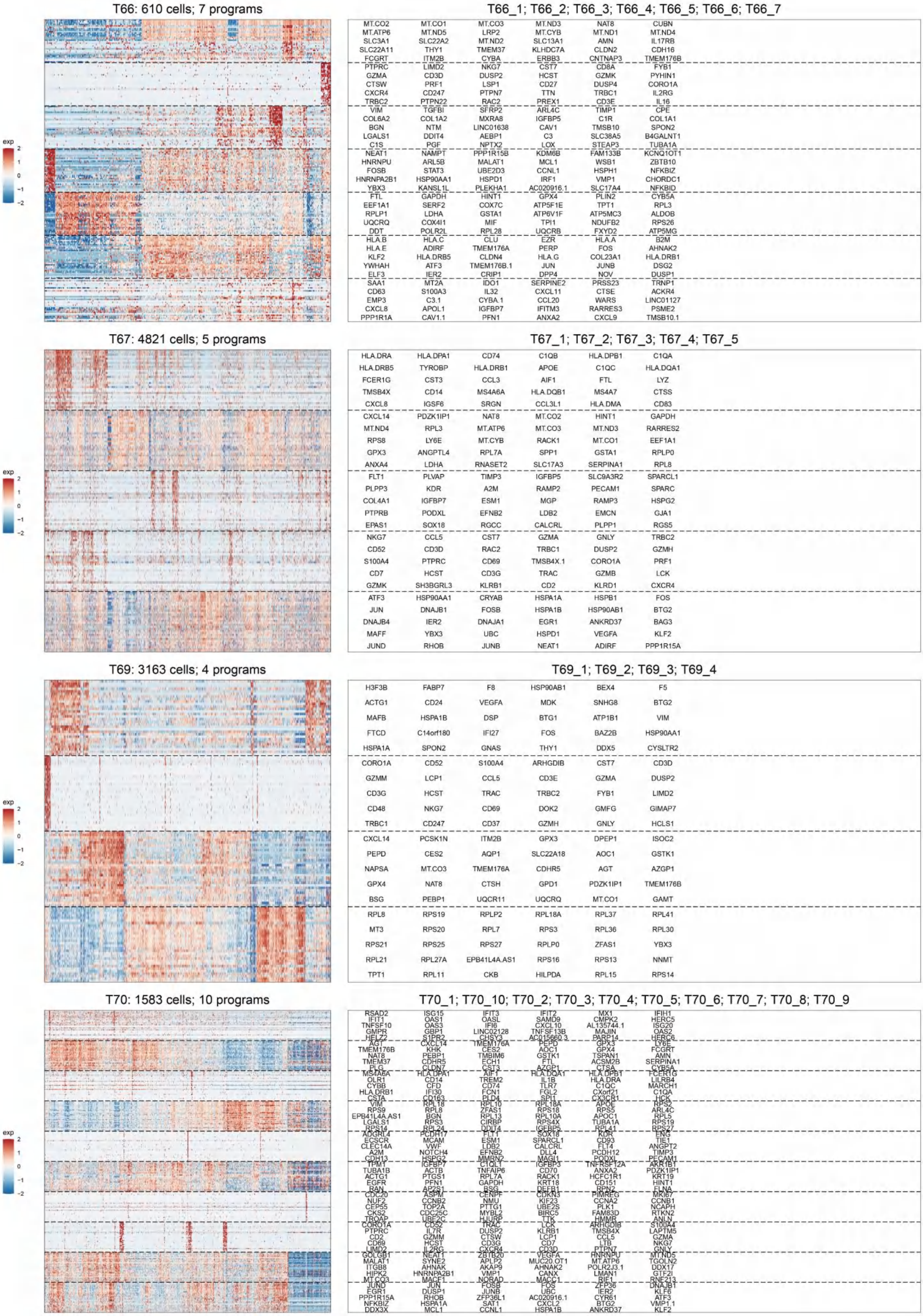
T66: 610 cells; 7 programs
exp
2
1
0
−1
−2
T66_1; T66_2; T66_3; T66_4; T66_5; T66_6; T66_7
MT.CO2 MT.CO1 MT.CO3 MT.ND3 NAT8 CUBN
MT.ATP6 MT.ND5 LRP2 MT.CYB MT.ND1 MT.ND4
SLC3A1 SLC22A2 MT.ND2 SLC13A1 AMN IL17RB
SLC22A11 THY1 TMEM37 KLHDC7A CLDN2 CDH16
FCGRT ITM2B CYBA ERBB3 CNTNAP3 TMEM176B
PTPRC LIMD2 NKG7 CST7 CD8A FYB1
GZMA CD3D DUSP2 HCST GZMK PYHIN1
CTSW PRF1 LSP1 CD27 DUSP4 CORO1A
CXCR4 CD247 PTPN7 TTN TRBC1 IL2RG
TRBC2 PTPN22 RAC2 PREX1 CD3E IL16
VIM TGFBI SFRP2 ARL4C TIMP1 CPE
COL6A2 COL1A2 MXRA8 IGFBP5 C1R COL1A1
BGN NTM LINC01638 CAV1 TMSB10 SPON2
LGALS1 DDIT4 AEBP1 C3 SLC38A5 B4GALNT1
C1S PGF NPTX2 LOX STEAP3 TUBA1A
NEAT1 NAMPT PPP1R15B KDM6B FAM133B KCNQ1OT1
HNRNPU ARL5B MALAT1 MCL1 WSB1 ZBTB10
FOSB STAT3 UBE2D3 CCNL1 HSPH1 NFKBIZ
HNRNPA2B1 HSP90AA1 HSPD1 IRF1 VMP1 CHORDC1
YBX3 KANSL1L PLEKHA1 AC020916.1 SLC17A4 NFKBID
FTL GAPDH HINT1 GPX4 PLIN2 CYB5A
EEF1A1 SERF2 COX7C ATP5F1E TPT1 RPL3
RPLP1 LDHA GSTA1 ATP6V1F ATP5MC3 ALDOB
UQCRQ COX4I1 MIF TPI1 NDUFB2 RPS26
DDT POLR2L RPL28 UQCRB FXYD2 ATP5MG
HLA.B HLA.C CLU EZR HLA.A B2M
HLA.E ADIRF TMEM176A PERP FOS AHNAK2
KLF2 HLA.DRB5 CLDN4 HLA.G COL23A1 HLA.DRB1
YWHAH ATF3 TMEM176B.1 JUN JUNB DSG2
ELF3 IER2 CRIP1 DPP4 NOV DUSP1
SAA1 MT2A IDO1 SERPINE2 PRSS23 TRNP1
CD63 S100A3 IL32 CXCL11 CTSE ACKR4
EMP3 C3.1 CYBA.1 CCL20 WARS LINC01127
CXCL8 APOL1 IGFBP7 IFITM3 RARRES3 PSME2
PPP1R1A CAV1.1 PFN1 ANXA2 CXCL9 TMSB10.1
T67: 4821 cells; 5 programs
exp
2
1
0
−1
−2
T67_1; T67_2; T67_3; T67_4; T67_5
HLA.DRA HLA.DPA1 CD74 C1QB HLA.DPB1 C1QA
HLA.DRB5 TYROBP HLA.DRB1 APOE C1QC HLA.DQA1
FCER1G CST3 CCL3 AIF1 FTL LYZ
TMSB4X CD14 MS4A6A HLA.DQB1 MS4A7 CTSS
CXCL8 IGSF6 SRGN CCL3L1 HLA.DMA CD83
CXCL14 PDZK1IP1 NAT8 MT.CO2 HINT1 GAPDH
MT.ND4 RPL3 MT.ATP6 MT.CO3 MT.ND3 RARRES2
RPS8 LY6E MT.CYB RACK1 MT.CO1 EEF1A1
GPX3 ANGPTL4 RPL7A SPP1 GSTA1 RPLP0
ANXA4 LDHA RNASET2 SLC17A3 SERPINA1 RPL8
FLT1 PLVAP TIMP3 IGFBP5 SLC9A3R2 SPARCL1
PLPP3 KDR A2M RAMP2 PECAM1 SPARC
COL4A1 IGFBP7 ESM1 MGP RAMP3 HSPG2
PTPRB PODXL EFNB2 LDB2 EMCN GJA1
EPAS1 SOX18 RGCC CALCRL PLPP1 RGS5
NKG7 CCL5 CST7 GZMA GNLY TRBC2
CD52 CD3D RAC2 TRBC1 DUSP2 GZMH
S100A4 PTPRC CD69 TMSB4X.1 CORO1A PRF1
CD7 HCST CD3G TRAC GZMB LCK
GZMK SH3BGRL3 KLRB1 CD2 KLRD1 CXCR4
ATF3 HSP90AA1 CRYAB HSPA1A HSPB1 FOS
JUN DNAJB1 FOSB HSPA1B HSP90AB1 BTG2
DNAJB4 IER2 DNAJA1 EGR1 ANKRD37 BAG3
MAFF YBX3 UBC HSPD1 VEGFA KLF2
JUND RHOB JUNB NEAT1 ADIRF PPP1R15A
T69: 3163 cells; 4 programs
exp
2
1
0
−1
−2
T69_1; T69_2; T69_3; T69_4
H3F3B FABP7 F8 HSP90AB1 BEX4 F5
ACTG1 CD24 VEGFA MDK SNHG8 BTG2
MAFB HSPA1B DSP BTG1 ATP1B1 VIM
FTCD C14orf180 IFI27 FOS BAZ2B HSP90AA1
HSPA1A SPON2 GNAS THY1 DDX5 CYSLTR2
CORO1A CD52 S100A4 ARHGDIB CST7 CD3D
GZMM LCP1 CCL5 CD3E GZMA DUSP2
CD3G HCST TRAC TRBC2 FYB1 LIMD2
CD48 NKG7 CD69 DOK2 GMFG GIMAP7
TRBC1 CD247 CD37 GZMH GNLY HCLS1
CXCL14 PCSK1N ITM2B GPX3 DPEP1 ISOC2
PEPD CES2 AQP1 SLC22A18 AOC1 GSTK1
NAPSA MT.CO3 TMEM176A CDHR5 AGT AZGP1
GPX4 NAT8 CTSH GPD1 PDZK1IP1 TMEM176B
BSG PEBP1 UQCR11 UQCRQ MT.CO1 GAMT
RPL8 RPS19 RPLP2 RPL18A RPL37 RPL41
MT3 RPS20 RPL7 RPS3 RPL36 RPL30
RPS21 RPS25 RPS27 RPLP0 ZFAS1 YBX3
RPL21 RPL27A EPB41L4A.AS1 RPS16 RPS13 NNMT
TPT1 RPL11 CKB HILPDA RPL15 RPS14
T70: 1583 cells; 10 programs
exp
2
1
0
−1
−2
T70_1; T70_10; T70_2; T70_3; T70_4; T70_5; T70_6; T70_7; T70_8; T70_9
RSAD2 ISG15 IFIT3 IFIT2 MX1 IFIH1
IFIT1 OAS1 OASL SAMD9 CMPK2 HERC5
TNFSF10 OAS3 IFI6 CXCL10 AL135744.1 ISG20
GMPR GBP1 LINC02128 TNFSF13B MAJIN OAS2
HELZ2 S1PR2 CHSY3 AC015660.3 PARP14 HERC6
AGT CXCL14 TMEM176A PEPD GPX3 LY6E
TMEM176B KHK CES2 AOC1 GPX4 FCGRT
NAT8 PEBP1 TMBIM6 GSTK1 TSPAN1 AMN
TMEM37 CDHR5 ECH1 FTL ACSM2B SERPINA1
PLG CLDN7 CST3 AZGP1 CTSA CYB5A
MS4A6A HLA.DPA1 AIF1 HLA.DQA1 HLA.DPB1 FCER1G
OLR1 CD14 TREM2 IL1B HLA.DRA LILRB4
CYBB CFD CD74 TLR7 C1QC MARCH1
HLA.DRB1 IFI30 FCN1 FGL2 CXorf21 C1QA
CSTA CD163 PLD4 SPI1 CX3CR1 HCK
VIM RPL18 RPL10 RPL18A APOE RPS2
RPS9 RPL8 ZFAS1 RPS18 RPS5 ARL4C
EPB41L4A.AS1 BGN RPL13 RPL10A APOC1 RPL5
LGALS1 RPS3 CIRBP RPS4X TUBA1A RPS19
RPS14 RPL24 DDIT4 IGFBP5 RPL41 RPS27
ADGRL4 PCDH17 FLT1 SOX18 KDR ENG
ECSCR MCAM ESM1 SPARCL1 CD93 TIE1
CLEC14A VWF LDB2 CALCRL FLT4 ANGPT2
A2M NOTCH4 EFNB2 DLL4 PCDH12 TIMP3
CDH13 HSPG2 MMRN2 MAGI1 PODXL PECAM1
TPM1 IGFBP7 C1QL1 IGFBP3 TNFRSF12A AKR1B1
TUBA1B ACTB TNFAIP6 CD70 ANXA2 PDZK1IP1
ACTG1 PTGS1 RPL7A RACK1 HCFC1R1 KRT19
EGFR PFN1 GAPDH KRT18 CD151 HINT1
RAN AP2S1 BSG DEFB1 RPN2 FLNA
CDC20 ASPM CENPF CDKN3 PIMREG MKI67
NUF2 CCNB2 NMU KIF23 CCNA2 CCNB1
CEP55 TOP2A PTTG1 UBE2S PLK1 NCAPH
CKS2 CDC25C MYBL2 BIRC5 FAM83D RTKN2
TROAP UBE2C HJURP TTK HMMR ANLN
CORO1A CD52 TRAC LCK ARHGDIB S100A4
PTPRC IL7R DUSP2 KLRB1 TMSB4X LAPTM5
CD2 GZMM CTSW LCP1 CCL5 GZMA
CD69 HCST CD3G CD7 LTB NKG7
LIMD2 IL2RG CXCR4 CD3D PTPN7 GNLY
GOLGB1 NEAT1 ZBTB20 VEGFA HNRNPU MT.ND5
MALAT1 SYNE2 APLP2 MUC20.OT1 MT.ATP6 TGOLN2
ITGB8 AHNAK AKAP9 AHNAK2 POLR2J3.1 DDX17
HIPK2 HNRNPA2B1 VMP1 CANX LMAN1 GTF2I
MT.CO3 MACF1 NORAD MACC1 RIF1 RNF213
JUND JUN FOSB FOS ZFP36 DNAJB1
EGR1 DUSP1 JUNB UBC IER2 KLF6
PPP1R15A RHOB ZFP36L1 AC020916.1 CYR61 ATF3
NFKBIZ HSPA1A SAT1 CXCL2 BTG2 VMP1.1
DDX3X MCL1 CCNL1 HSPA1B ANKRD37 KLF2

T71: 5469 cells; 8 programs

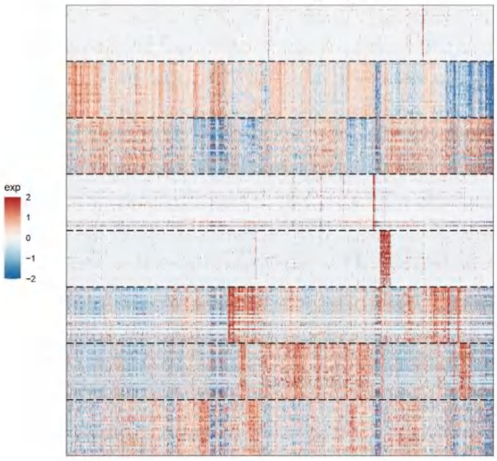

T71_1; T71_2; T71_3; T71_4; T71_5; T71_6; T71_7; T71_8

CLEC14A	IL33	A2M	FAM198B	EFNB2	MGP
CD34	PECAM1	RAMP2	ADGRL4	ENG	MMRN2
CDH5	EMCN	S1PR1	CLDN5	FLT1	GJA4
CD93	FN1	LMO2	SPARCL1	VEGFC	SLC9A3R2
RBP7	VWF	PODXL	ADGRF5	ADAMTS5	FBN1
RPS2	RPL28	RPL41	RPS18	RPL7A	RACK1
RPS12	RPL18A	RPS15	RPL29	RPL19	RPL12
RPL13	RPL10A	RPL3	RPS5	RPS23	RPS8
RPS4X	RPS7	RPS3	RPLP0	RPS14	RPS15A
RPS9	RPLP1	RPS6	RPL6	RPL32	RPL11
MALAT1	MT.ATP6	VEGFA	MT.ND1	RPL23	MT.ND2
HNRNPU	GOLGB1	VMP1	NEAT1	MT.ND3	FUS
ZBTB20	RPS11	MT.ND4	HNRNPA2B1	SRRM2	KCNQ1OT1
MT.ND5	FOSB	CCNL1	HSP90AB1	RBM39	RPS20
ZNF292	STAT3	DDX17	DDX5	SF1	PNISR
TPX2	BIRC5	MKI67	CENPF	CDKN3	CDCA3
KIF20A	ASPM	CCNB2	PTTG1	CDC20	UBE2C
CCNB1	GTSE1	CEP55	AURKB	STMN1	KIFC1
TOP2A	PBK	SAPCD2	PLK1	CENPA	HJURP
KIF23	TUBA1B	ANLN	H2AFZ	CCNA2	SPC25
CD2	CST7	CCL5	GZMA	CORO1A	CD3D
NKG7	IL2RG	PTPRC	TRBC2	LCP1	TRAC
HCST	CTSW	SRGN	LIMD2	GZMK	DUSP2
CD3G	RGS1	GMFG	CD3E	CD27	CD69
TNFRSF9	CD37	CD247	CD8A	IKZF3	SLA
HLA.DRA	CD74	HLA.DQA2	GBP4	RARRES3	HLA.C
HLA.DQA1	HLA.DRB5	B2M	PSMB9	HLA.DPA1	HLA.A
HLA.DRB1	GBP1	HLA.B	CXCL10	HLA.DQB1	IDO1
HLA.DPB1	HLA.E	LAP3	CXCL11	HLA.DMA	CXCL9
WARS	TNFSF10	CTSS	IRF1	IFI27	HLA.F
NAT8	CXCL14	CUBN	PDZK1IP1	GPX3	CES2
APOM	AMN	SLC6A13	UQCRQ	CYB5A	AGT
BBOX1	KHK	SMIM24	AQP1	COX6A1	CDHR5
SLC5A12	SLC17A3	TM4SF5	GAL3ST1	PRAP1	PEPD
AOC1	BSG	DAB2	DNPH1	TXNDC17	SLC39A5
UBC	CRYAB	DUSP1	CLDN4	NDUFA4L2	CD9
PKM	TMEM176A	JUNB	KRT7	JUND	CRIP2
TMEM176B	CLU	S100A10	TACSTD2	ZFP36	PGK1
RHOB	CLDN3	HSPA1A	KLF2	HSPB1	FOS
HSPA1B	TPI1	CLDN7	ADIRF	IER2	EIF1

T72: 2433 cells; 5 programs

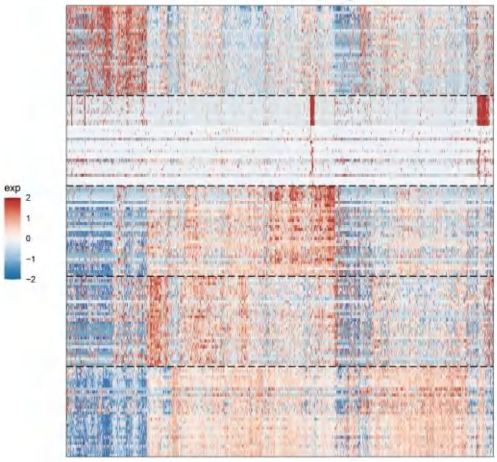

T72_1; T72_2; T72_3; T72_4; T72_5

MALAT1	NEAT1	POLR2J3.1	HNRNPA2B1	HNRNPU	VMP1
WSB1	PNISR	KCNQ1OT1	PLEKHH2	ATP13A3	DDX17
N4BP2L2	ZEB2	PLAGL1	FTX	CCNL1	MACF1
PIAS2	PAX8	VEGFA	PLEKHA1	MUC20.OT1	WDR60
ZNF83	MACC1	GLS	ABCC3	SYNE2	ARGLU1
MT.ND3	MT.ND4	MT.ATP6	MT.ND1	MT.CYB	MT.CO1
MT.ND2	MT.ND5	MT.CO3	MT.CO2	MZB1	IGHG3
IGHG4	IGHG1	IGKC	MT.ND4L	JCHAIN	CD3E
DUSP2	MT.ND6	IL7R	TMSB4X	SRGN	CD69
CD52	MTRNR2L12	CD74	PTPN22	ITGAL	RCSD1
NAT8	GPX4	KHK	BBOX1	LGALS2	ECH1
SLC6A18	CYB5A	MPC2	TMEM176B	DNPH1	GSTA1
ATP5MC3	GAL3ST1	PXMP2	LDHB	NDUFB2	AQP1
TMEM176A	HINT1	ANXA4	CXCL14	OCIAD2	IMPA2
CUBN	PTN	COX5B	RPS28	PRDX2	CHCHD10
TNFRSF12A	S100A11	TM4SF1	PFN1	ACTB	ACTG1
CTSZ	MT2A	KRT19	ANXA2	IGFBP7	CITED4
PDZK1IP1	TNFAIP6	TPM1	CLIC1	CFL1	TUBA1B
RAN	EMP3	MYL6	PHLDA1	PPP1R14B	FHL2
IL32	CAV1	TRAM1	PRSS23	CCL2	MYL12A
FOS	JUNB	EIF1	DUSP1	RPL10	RPL41
TPT1	KLF2	HSPA1A	C11orf96	RPL21	HSPA1B
ATF3	IER2	RHOB	DNAJB1	RPL23A	VIM
RPL9	RPS14	FTH1	RPL30	RPS27A	CCNI
RPS27	RPS5	UBC	RPS3	RPS2	RPL18A

T73: 309 cells; 6 programs

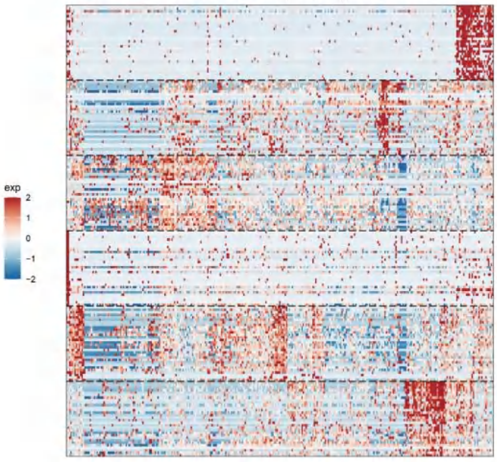

T73_1; T73_2; T73_3; T73_4; T73_5; T73_6

CD3G	TRAC	TMSB4X	IL2RG	PTPRC	TIGIT
GPSM3	LSP1	CD3D	GZMK	LAPTM5	CD27
CD2	DUSP4	ARHGDIB	ITM2A	CORO1A	EMB
HCST	TBC1D10C	UCP2	CTSW	CD52	LCP1
LCK	SPN	RAC2	GIMAP4	SRGN	TRBC2
THBS1	XIST	SYNE2	DDX17	VEGFA	SYNPO
SBNO2	LINC00472	NEAT1	MALAT1	EP400	SYNE1
MT.CO3	ZNF292	LIFR	ALPK2	LRP2	ZNF462
AHNAK	TBC1D8B	HNRNPU	LRCH1	AKAP9	SLCO3A1
IL6ST	BRAT1	SAMD4A	RRBP1	RC3H1	MKLN1
CYB5A	SLC6A13	LGALS2	NDUFC1	GAPDH	TMEM176B
CXCL14	TESC	MPC2	AC021744.1	ABCB9	BBOX1
TMEM139	TMEM176A	FBP1	GAL3ST1	DUSP28	RACK1
FTL	TPT1	PDZK1IP1	SERF2	TSTD1	CMBL
TPI1	SLC17A3	ADIRF	S100A1	MIF	HIST1H1C
IGSF6	SPI1	C1QB	FCER1G	C1QC	A2M
CD14	C1QA	HLA.DPB1	TYROBP	APOC1	CTSS
MS4A6A	ADA2	CST3	LY96	APOE	GPR65
CD4	FCGR2A	AIF1	LST1	HLA.DRA	LAPTM5.1
HLA.DQB1	RGS10	PTGER4	DSE	NRP2	HLA.DPA1
CXCL11	TNFSF10	MARCKSL1	RARRES3	C3	VAMP5
GBP1	C1R	CAV1	TYMP	IGFBP3	IL32
LY6E	IL18BP	PSMB9	NNMT	PSME1	CXCL9
SELENOM	TIMP1	CD40	S100A11	TAP1	PHLDA2
IFITM3	TMSB10	WARS	FBXL6	SPIB	EMP3
FOS	IER2	JUNB	JUND	ZFP36	SOCS3
UBC	ATF3	EGR1	JUN	FOSB	SERTAD1
DUSP1	HSPA1B	KLF2	PPP1R15A	MAFF	GDF15
BTG2	CDKN1A	CEBPD	EIF1	KLF4	NFKBIA
YBX3	MT.ND1	ST7.AS1	GADD45B	H3F3B	DNAJB1

T74: 1603 cells; 5 programs

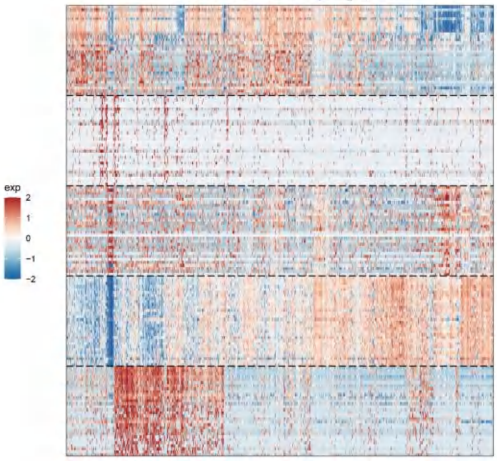

T74_1; T74_2; T74_3; T74_4; T74_5

MT.CO3	CXCL14	MT.CO2	MT.ATP6	PDZK1IP1	MT.CO1
MT.ND4	MT.ND3	MT.CYB	KRT18	CYB5A	TMEM176A
CRYAB	GPX3	HINT1	SLC17A3	CES2	GSTA2
FXYD2	SPP1	CNDP2	GSTA1	TMEM176B	SERPINA1
ANGPTL4	LGALS2	RBP5	MT.ND5	ANXA4	CKB
FLT1	ENG	SLC9A3R2	ADGRL4	COL4A1	HSPG2
VWF	CD34	SPRY1	COL4A2	PODXL	PLVAP
PECAM1	RAMP3	KDR	SULF1	CALCRL	ADGRF5
TIMP3	TM4SF1	TCF4	COL15A1	SPARCL1	EMP1
HEG1	INSR	A2M	PLXND1	CLEC14A	SH3BP5
SQSTM1	MALAT1	ATF3	TCEA1	BEX2	NEAT1
HSPA9	ZBTB20	MUC20.OT1	NUPR1	FAM13A	VEGFA
PDK4	FOSB	IGFBP5	PLCG2	AC025423.1	VMP1
CPEB4	GADD45A	N4BP2L2	ANXA1	RUFY3	PNISR
PDLIM3	ZFAND5	FTL	HSP90AA1	DDX17	MSC.AS1
RPL41	RPS14	RPL10	RPS27	RPL18	RPS11
RPS20	RPL13	RPL11	RPS8	RPL36	RPL23
RPS5	RPL24	APOE	RPL39	RPS28	RPL9
RPL35A	RPL19	VIM	RPS18	RPL37A	RPL18A
RPS3	RPL30	RPS15	LGALS1	RPL27A	RPL23A
HLA.DRA	HLA.DPB1	HLA.DPA1	CD74	HLA.DRB5	TYROBP
HLA.DRB1	SRGN	RGS1	TMSB4X	CST3	C1QA
SAT1	AIF1	HLA.DQA1	C1QB	FCER1G	CXCR4
B2M	CTSS	LYZ	HLA.DQB1	SGK1	LAPTM5
FGL2	C1QC	MS4A7	HLA.DMA	GPR183	HERPUD1

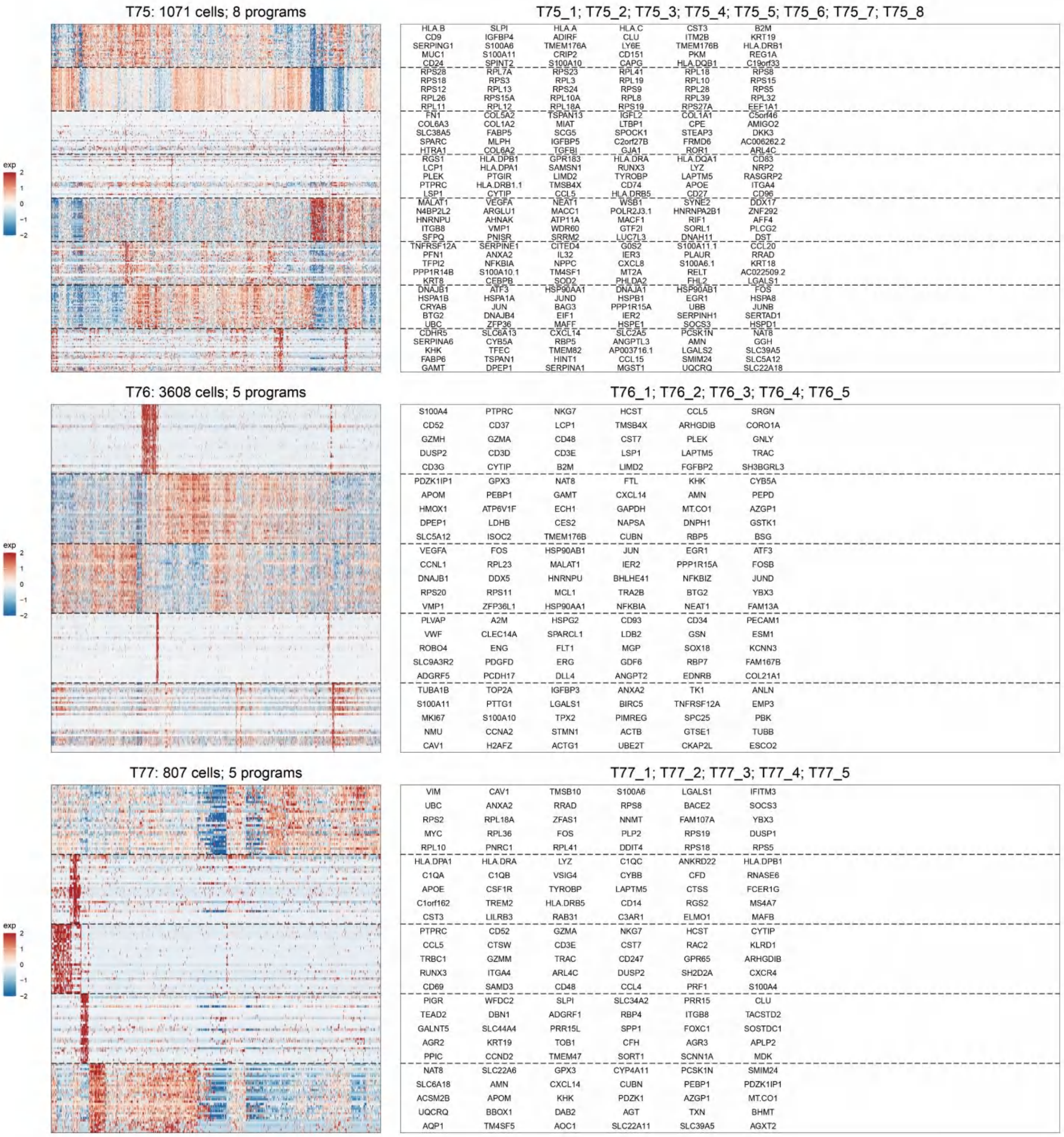

Figure S6-S16 Gene expression within each gene expression program in each sample.

Heatmap showing expression of genes within each gene expression program (GEP) across single cells in each tumor sample. Randomly selected 5% cells for each program are shown, with top 30 signature genes showed on the right. cNMF analysis was performed in 43 tumor samples separately using the optimal number of NMF components (k-value).

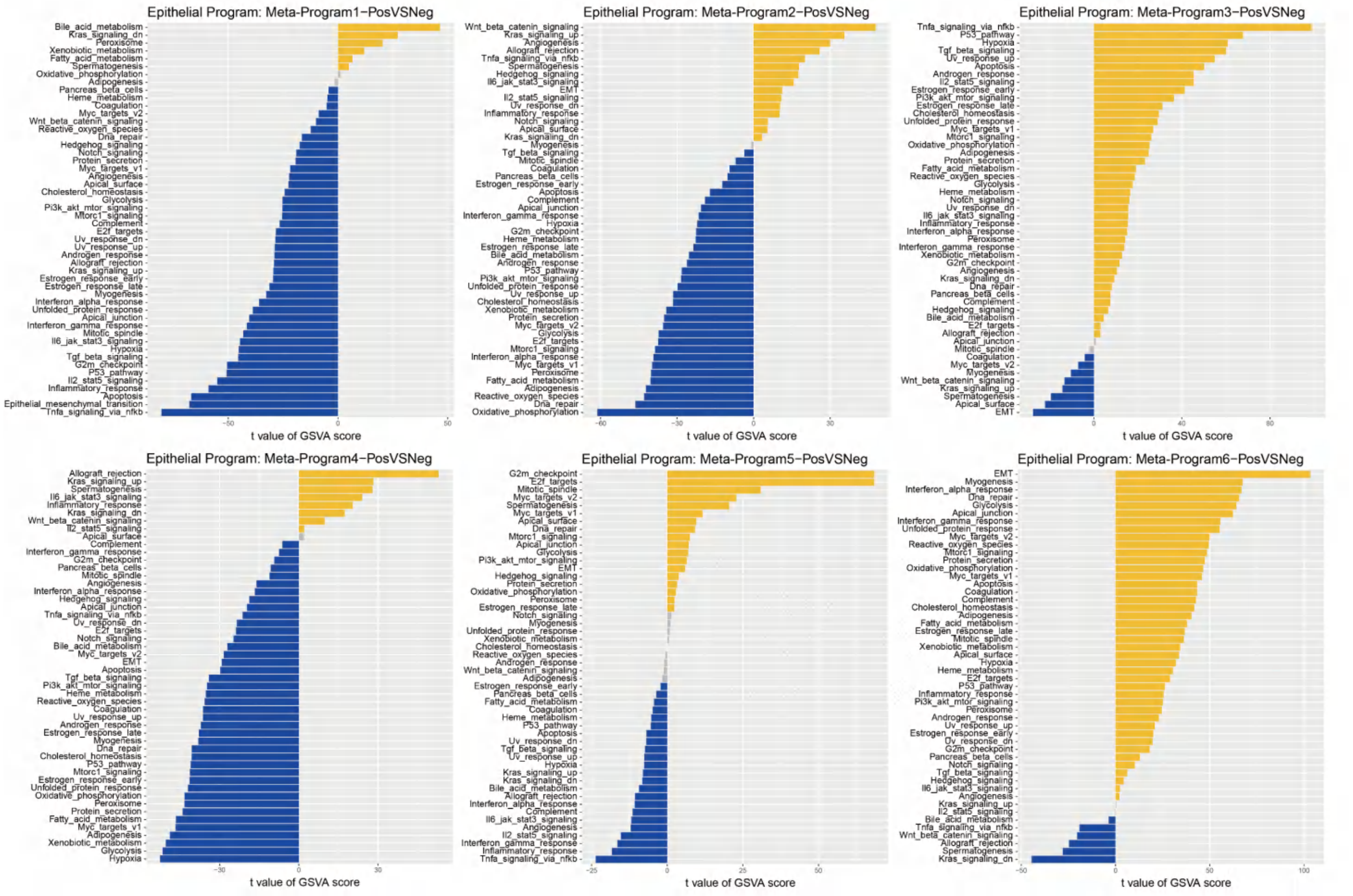

Figure S17 Hallmark pathways enrichment of six malignant meta-programs.

Diverging bar plots showing differences in pathway activities scored per cell by GSVA between program tumor cells and non-program tumor cells. t value of GSVA score was corrected for patient of origin. UV, ultraviolet; dn, down; v1, version 1; v2, version 2.

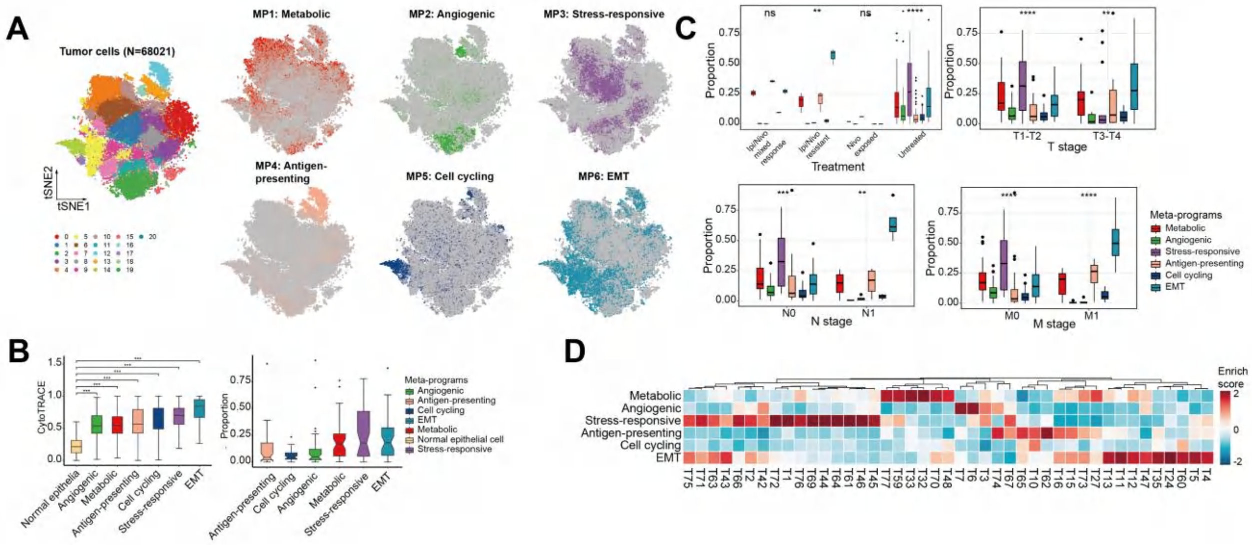

Figure S18 Distributions and clinical associations of six program tumor cells.

(A) tSNE plot showing clusters of malignant epithelial cells (left), with colored by enrichment score of each meta-program (right). (B) Boxplot showing the CytoTRACE score (left) and the proportion of program cells

for each tumor (n = 43) (right) among six MPs. (C) Boxplots showing proportion of six malignant epithelial cells in different ICB treatment, T stage, N stage, and M stage groups. (D) Heatmap showing the normalized enrichment scores for six MPs in each tumor. All samples are distinctly assigned to a specific tumor state.

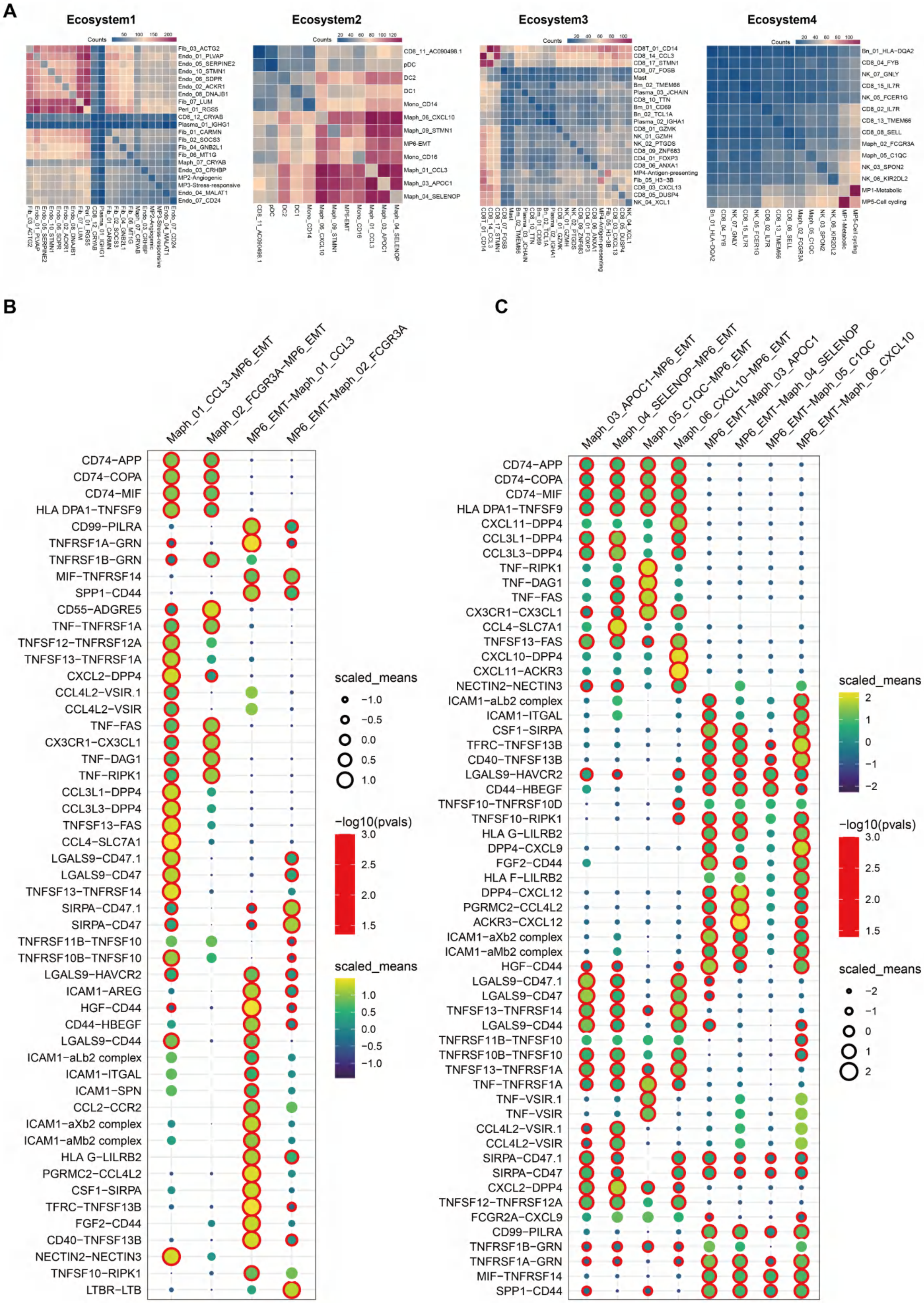
A
Ecosystem1
Ecosystem2
Ecosystem3
Ecosystem4
Counts
B
Maph_01_CCL3-MP6_EMT
Maph_02_FCGR3A-MP6_EMT
MP6_EMT-Maph_01_CCL3
MP6_EMT-Maph_02_FCGR3A
CD74−APP
CD74−COPA
CD74−MIF
HLA DPA1−TNFSF9
CD99−PILRA
TNFRSF1A−GRN
TNFRSF1B−GRN
MIF−TNFRSF14
SPP1−CD44
CD55−ADGRE5
TNF−TNFRSF1A
TNFSF12−TNFRSF12A
TNFSF13−TNFRSF1A
CXCL2−DPP4
CCL4L2−VSIR.1
CCL4L2−VSIR
TNF−FAS
CX3CR1−CX3CL1
TNF−DAG1
TNF−RIPK1
CCL3L1−DPP4
CCL3L3−DPP4
TNFSF13−FAS
CCL4−SLC7A1
LGALS9−CD47.1
LGALS9−CD47
TNFSF13−TNFRSF14
SIRPA−CD47.1
SIRPA−CD47
TNFRSF11B−TNFSF10
TNFRSF10B−TNFSF10
LGALS9−HAVCR2
ICAM1−AREG
HGF−CD44
CD44−HBEGF
LGALS9−CD44
ICAM1−aLb2 complex
ICAM1−ITGAL
ICAM1−SPN
CCL2−CCR2
ICAM1−aXb2 complex
ICAM1−aMb2 complex
HLA G−LILRB2
PGRMC2−CCL4L2
CSF1−SIRPA
TFRC−TNFSF13B
FGF2−CD44
CD40−TNFSF13B
NECTIN2−NECTIN3
TNFSF10−RIPK1
LTBR−LTB
scaled_means
−1.0
−0.5
0.0
0.5
1.0
−log10(pvals)
3.0
2.5
2.0
1.5
scaled_means
1.0
0.5
0.0
−0.5
−1.0
C
Maph_03_APOC1-MP6_EMT
Maph_04_SELENOP-MP6_EMT
Maph_05_C1QC-MP6_EMT
Maph_06_CXCL10-MP6_EMT
MP6_EMT-Maph_03_APOC1
MP6_EMT-Maph_04_SELENOP
MP6_EMT-Maph_05_C1QC
MP6_EMT-Maph_06_CXCL10
CD74−APP
CD74−COPA
CD74−MIF
HLA DPA1−TNFSF9
CXCL11−DPP4
CCL3L1−DPP4
CCL3L3−DPP4
TNF−RIPK1
TNF−DAG1
TNF−FAS
CX3CR1−CX3CL1
CCL4−SLC7A1
TNFSF13−FAS
CXCL10−DPP4
CXCL11−ACKR3
NECTIN2−NECTIN3
ICAM1−aLb2 complex
ICAM1−ITGAL
CSF1−SIRPA
TFRC−TNFSF13B
CD40−TNFSF13B
LGALS9−HAVCR2
CD44−HBEGF
TNFSF10−TNFRSF10D
TNFSF10−RIPK1
HLA G−LILRB2
DPP4−CXCL9
FGF2−CD44
HLA F−LILRB2
DPP4−CXCL12
PGRMC2−CCL4L2
ACKR3−CXCL12
ICAM1−aXb2 complex
ICAM1−aMb2 complex
HGF−CD44
LGALS9−CD47.1
LGALS9−CD47
TNFSF13−TNFRSF14
LGALS9−CD44
TNFRSF11B−TNFSF10
TNFRSF10B−TNFSF10
TNFSF13−TNFRSF1A
TNF−TNFRSF1A
TNF−VSIR.1
TNF−VSIR
CCL4L2−VSIR.1
CCL4L2−VSIR
SIRPA−CD47.1
SIRPA−CD47
CXCL2−DPP4
TNFSF12−TNFRSF12A
FCGR2A−CXCL9
CD99−PILRA
TNFRSF1B−GRN
TNFRSF1A−GRN
MIF−TNFRSF14
SPP1−CD44
scaled_means
2
1
0
−1
−2
−log10(pvals)
3.0
2.5
2.0
1.5
scaled_means
−2
−1
0
1
2

Figure S19 Analysis of ligand-receptor interactions in tumor ecosystems using CellphoneDB.

(A) Heatmaps of L-R interactions across four tumor ecosystems. Heatmaps display the intensity of L-R interactions within four distinct tumor ecosystems, labeled Ecosystem1 through Ecosystem4. The color scale ranges from blue (low interaction) to red (high interaction), with the counts of interactions indicated by the color intensity. These heatmaps highlight the heterogeneity and unique interaction profiles of each tumor microenvironment. (B) Dot plot of L-R interactions between EMT tumor cells and M1 macrophages in Ecosystem2. This dot plot details the interaction significance and expression levels of L-R pairs between EMT tumor cells and M1 macrophages within Ecosystem2. The dot size denotes the -log10 (p-value), reflecting the statistical significance of each interaction, with larger dots indicating more significant findings. The color of the dots varies from green to red, representing the scaled mean expression levels from low to high, respectively. Key interactions such as CD74-APP, CD74-MIF, and TNF-FAS are highlighted, showing their relevance in these ecosystems. (C) Dot plot of L-R interactions between EMT tumor cells and M2 macrophages in Ecosystem2. Mirroring the structure of (B), this dot plot shows L-R interactions between EMT tumor cells and M2 macrophages in Ecosystem2. It uses the same conventions for dot size and color coding to indicate statistical significance and expression levels. This panel focuses on illustrating the distinct interaction patterns that may influence the dynamics within the tumor microenvironment specifically involving M2 macrophages. Notable interactions include TNF-FAS, ICAM1-ITGAL, and CXCL12-CXCR4, which may play critical roles in the tumor microenvironment dynamics of these ecosystems.

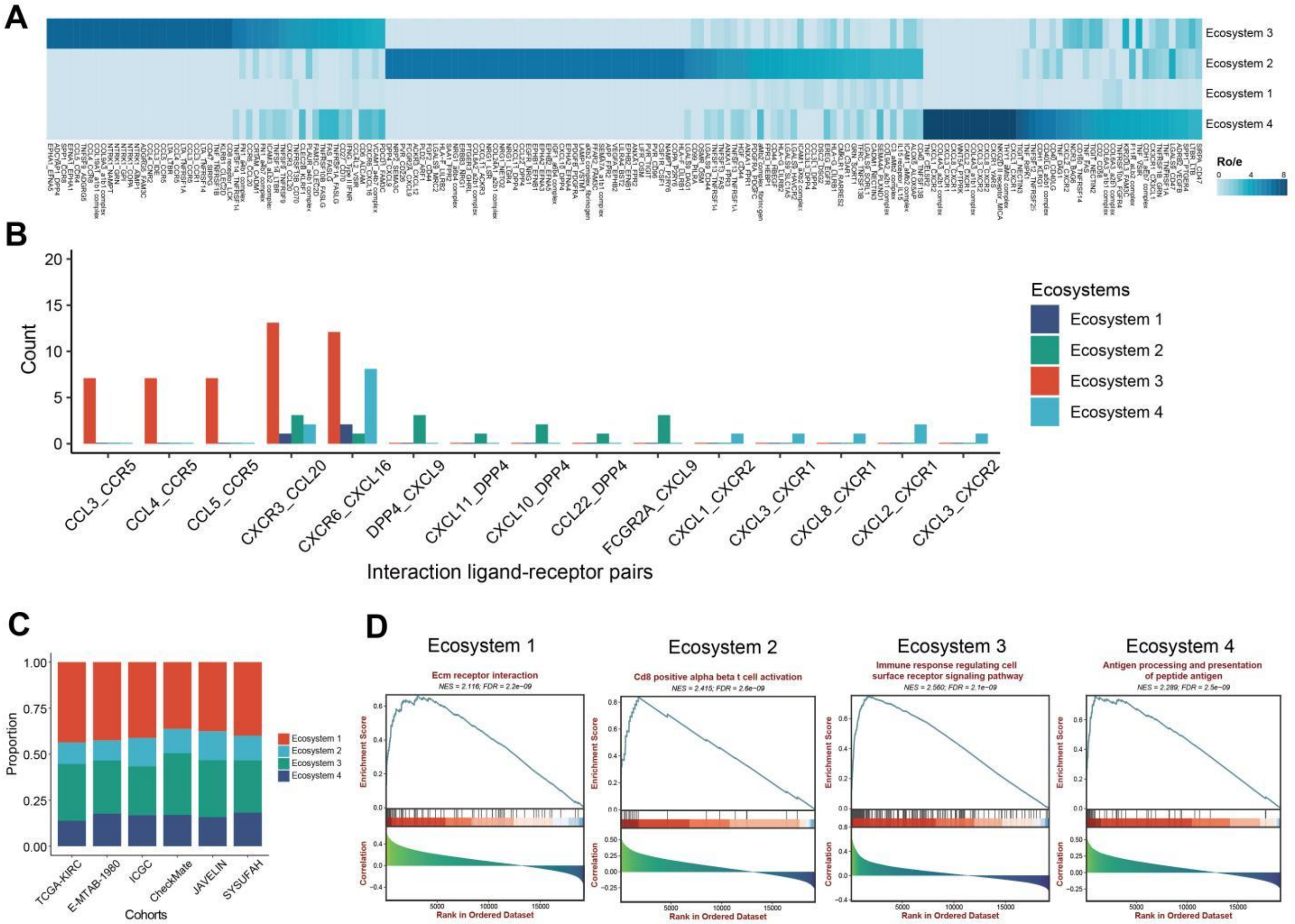

Figure S20 Ligand-receptor interactions, distribution, and signaling pathways across tumor ecosystems. (A) Heatmap showing enriched L-R pairs in four specific ecosystems, colored coded by $R_{o/e}$ enrichment values. L-R pair with $R_{o/e} > 1$ was assumed to enriched in a specific ecosystem. (B) Barplots showing the frequency of key chemokines/chemokine receptors pairs significantly enriched in four distinct ecosystems. (C) Stacked bar chart showing the proportional distribution of four tumor ecosystems in five independent cohorts (TCGA, E-MTAB-1980, ICGC, CheckMate, JAVELIN, and SYSUFAH). (D) GSEA plot visualizing significantly enriched hallmark signaling pathways of four tumor ecosystems. The normalized enrichment score (NES) and GSEA false discovery rate (FDR) are indicated for key pathways. Pathways with $|NES| > 1$ and $FDR < 0.05$ were deemed significant.

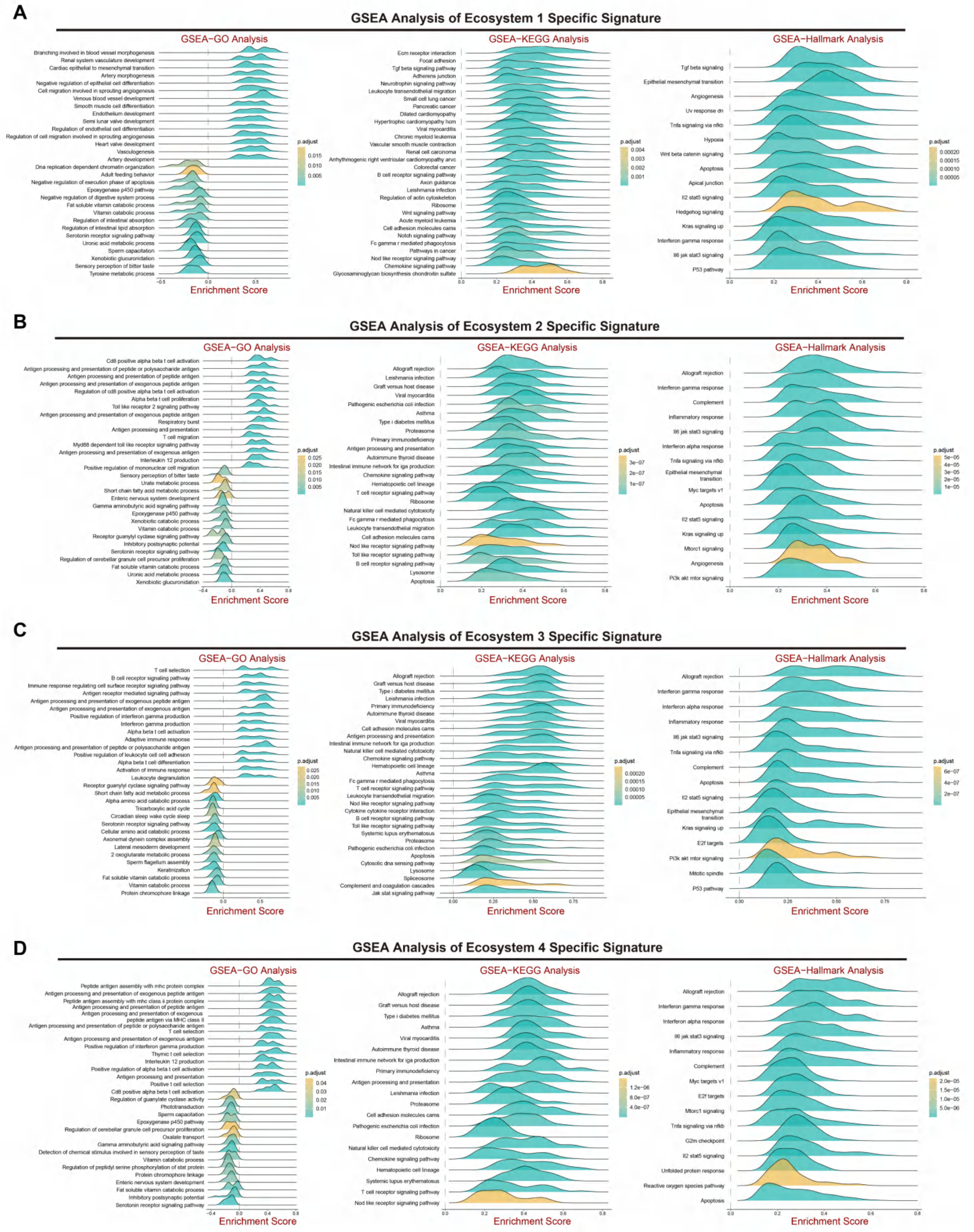

Figure S21 GSEA ridge plot analysis of tumor ecosystem signatures across GO, KEGG, and Hallmark gene sets.

(A-D) Ridge plots visualize the enrichment patterns of four tumor ecosystem signatures (Ecosystems 1-4)

against gene sets from GO, KEGG, and Hallmark databases using Gene Set Enrichment Analysis (GSEA). Each "ridge" represents the distribution of enrichment scores for a significantly enriched pathway, where the peak corresponds to the maximum enrichment score (ES) and the width reflects the density of genes contributing to the enrichment. The x-axis indicates the ES (positive/negative values denote up-/down-regulation in the signature), and the y-axis lists enriched pathways. Color intensity represents the statistical significance. Pathways with |NES| > 1 and FDR < 0.05 were deemed significant. The analysis reveals distinct biological functions associated with each ecosystem.

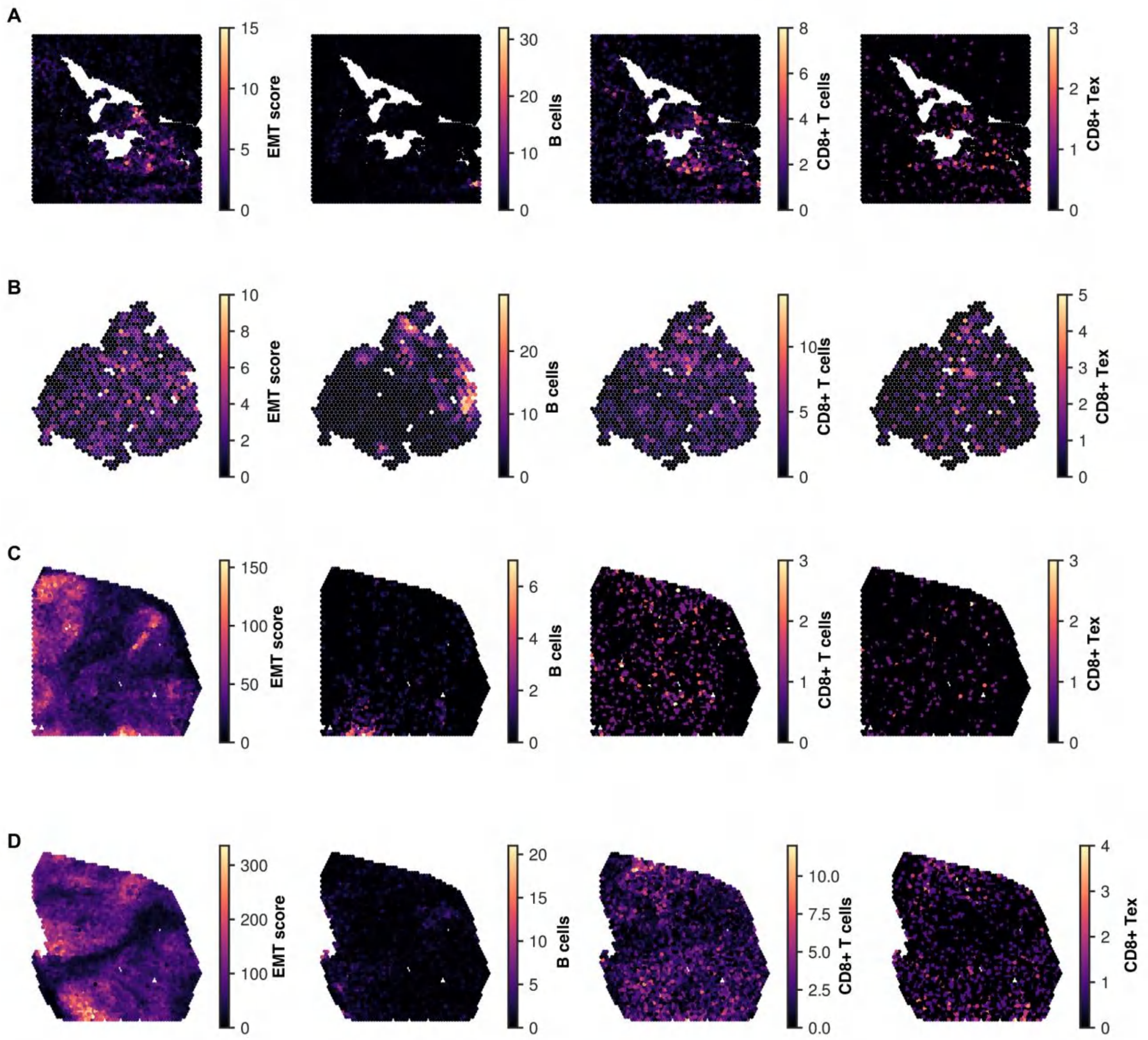

Figure S22 Spatial co-localization analysis between EMT-high tumor cells and specific immune cell types.

(A-D) Representative spatial transcriptomics map showing the distribution of EMT-high tumor cells, B cells, CD8+ T cells, and exhausted CD8+ T cells within the tumor microenvironment. Color gradient indicates signature score intensity. EMT-high tumor cells exhibit spatial proximity to CD8+ T cells and exhausted CD8+ T cells.

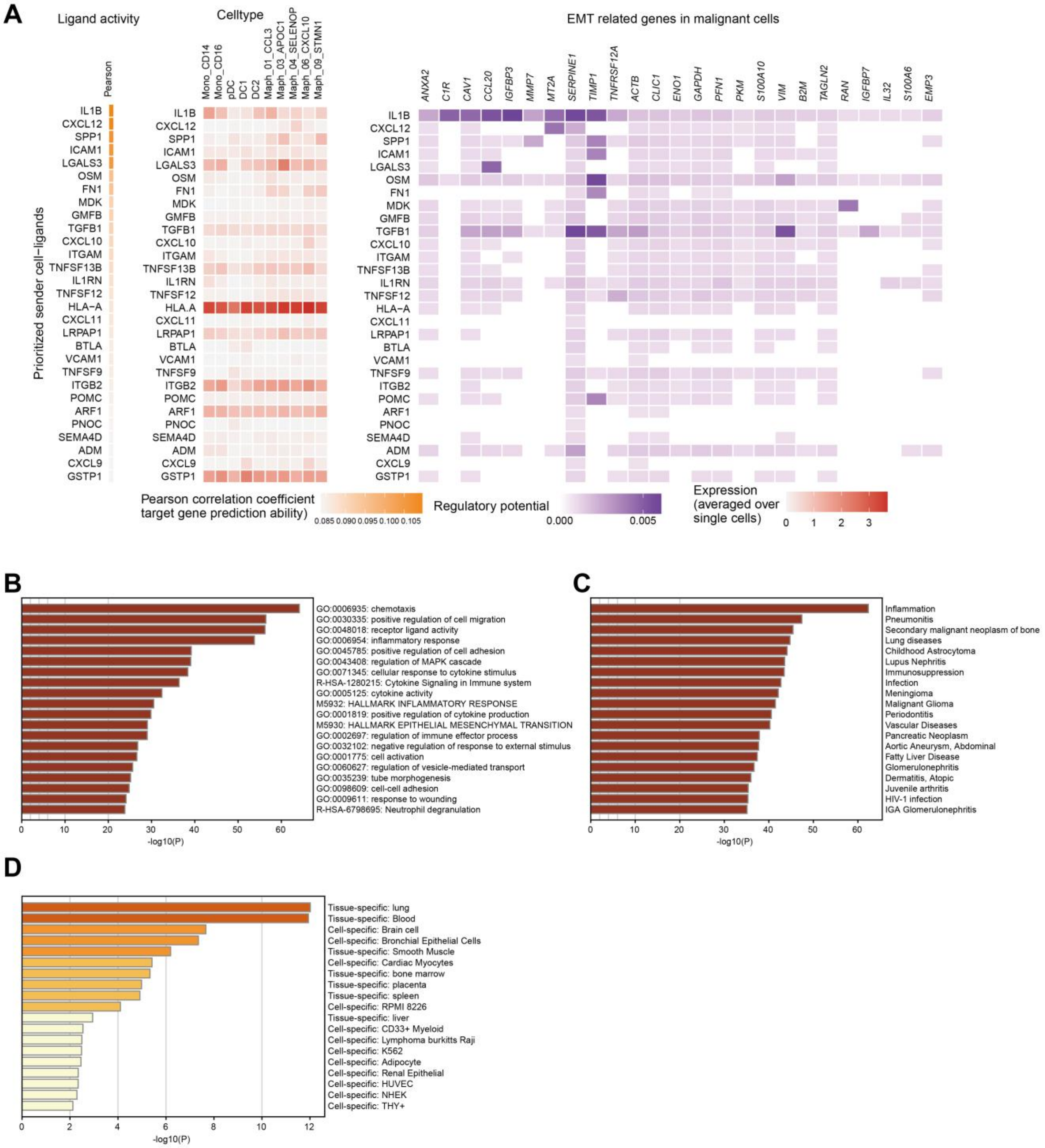

Figure S23 NicheNet analysis and functional enrichment of the ecosystem 2 signature.

(A) Outcome of predicted NicheNet's ligand activity by myeloid cells on EMT related genes. Heatmap showing the Pearson correlation indicates the target genes prediction ability of each ligand, and better predictive ligands are thus ranked higher. (B-D) Metascape enrichment analysis of the signature associated with ecosystem 2. Significant enrichments are shown for (B) epithelial-mesenchymal transition (EMT)-related pathways, (C) immunosuppression-related pathways, and (D) myeloid cell-related pathways. The consistent enrichments across categories validate the robustness of the signature. Metascape terms with adjusted $P < 0.05$.

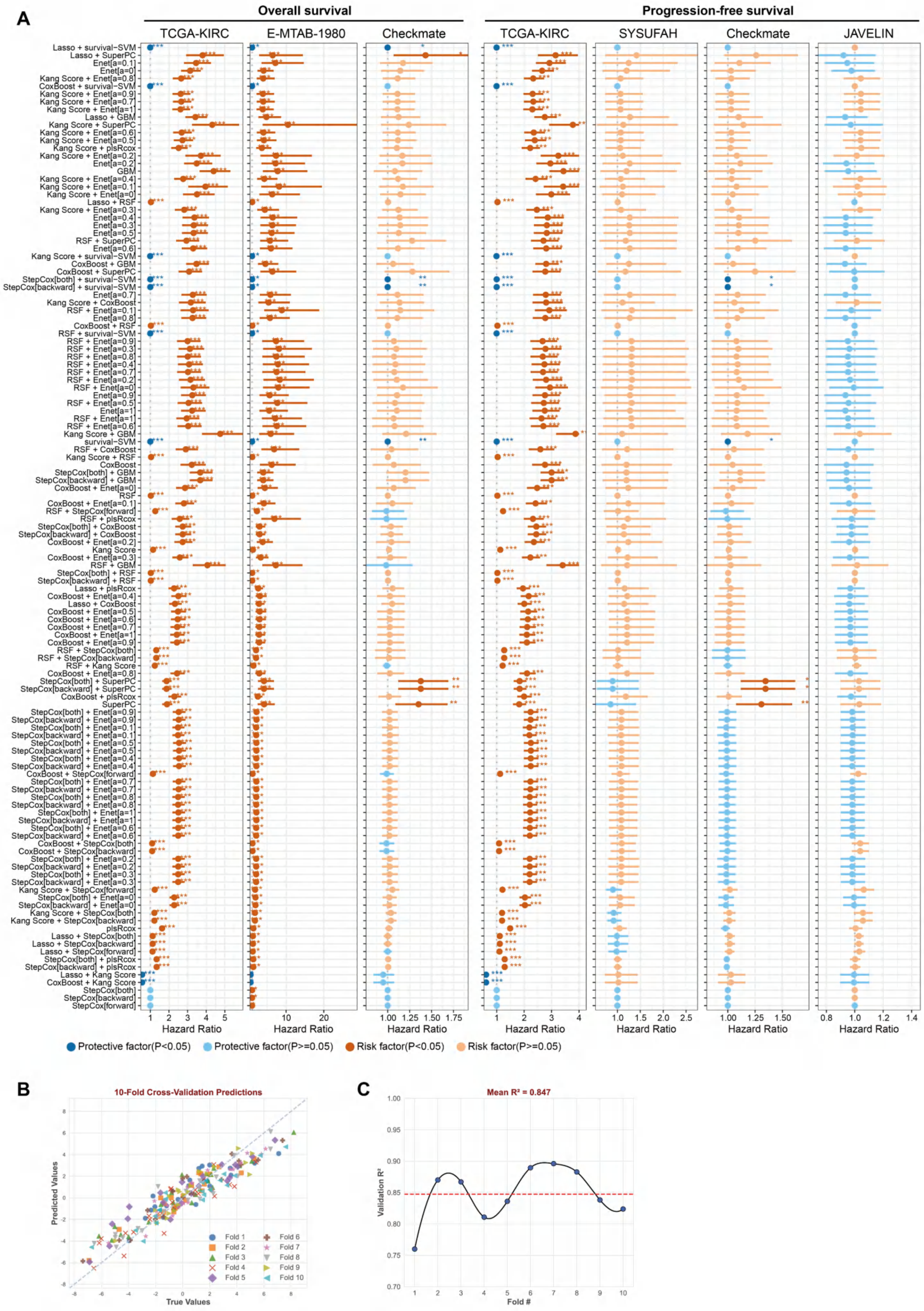
A
Overall survival
Progression-free survival
TCGA-KIRC
E-MTAB-1980
Checkmate
TCGA-KIRC
SYSUFAH
Checkmate
JAVELIN
Hazard Ratio
Protective factor(P<0.05)
Protective factor(P>=0.05)
Risk factor(P<0.05)
Risk factor(P>=0.05)
B
10-Fold Cross-Validation Predictions
Predicted Values
True Values
Fold 1
Fold 2
Fold 3
Fold 4
Fold 5
Fold 6
Fold 7
Fold 8
Fold 9
Fold 10
C
Mean R² = 0.847
Validation R²
Fold #

Figure S24. Assessment of prognostic model performance through forest plot and 10-fold cross-validation.

(A) Forest plot of hazard ratios for 126 prognostic models across multiple validation cohorts. The prognostic performance of each model is visualized by its HR (with 95% confidence intervals) for overall survival (OS) in the TCGA-KIRC, EMTAB-1980, CheckMate cohorts and progression-free survival (PFS) in the TCGA, SYSUFAH, CheckMate, JAVELIN cohorts. Each horizontal line represents the HR and confidence interval for a single model within a specific cohort and endpoint. The color of each marker indicates the effect direction and statistical significance: red (HR > 1, $P < 0.05$), light red (HR > 1, $P \geq 0.05$), blue (HR < 1, $P < 0.05$), light blue (HR < 1, $P \geq 0.05$). The vertical line (line of no effect) is set at HR = 1. Statistical significance was defined as a two-sided $P < 0.05$. Abbreviations: HR, hazard ratio; TCGA, The Cancer Genome Atlas. (B) Parity plot from 10-fold cross-validation of the optimal model, comparing the actual versus predicted prognostic values for each fold. Markers represent individual folds, and the solid diagonal line denotes the ideal 1:1 relationship. The close alignment of data points to the diagonal indicates consistent model performance across different data subsets. (C) Coefficient of determination (R^2) for each of the 10 cross-validation folds. The average R^2 value is 0.847, indicating that the model explains 84.7% of the variance and demonstrates robust predictive capability.

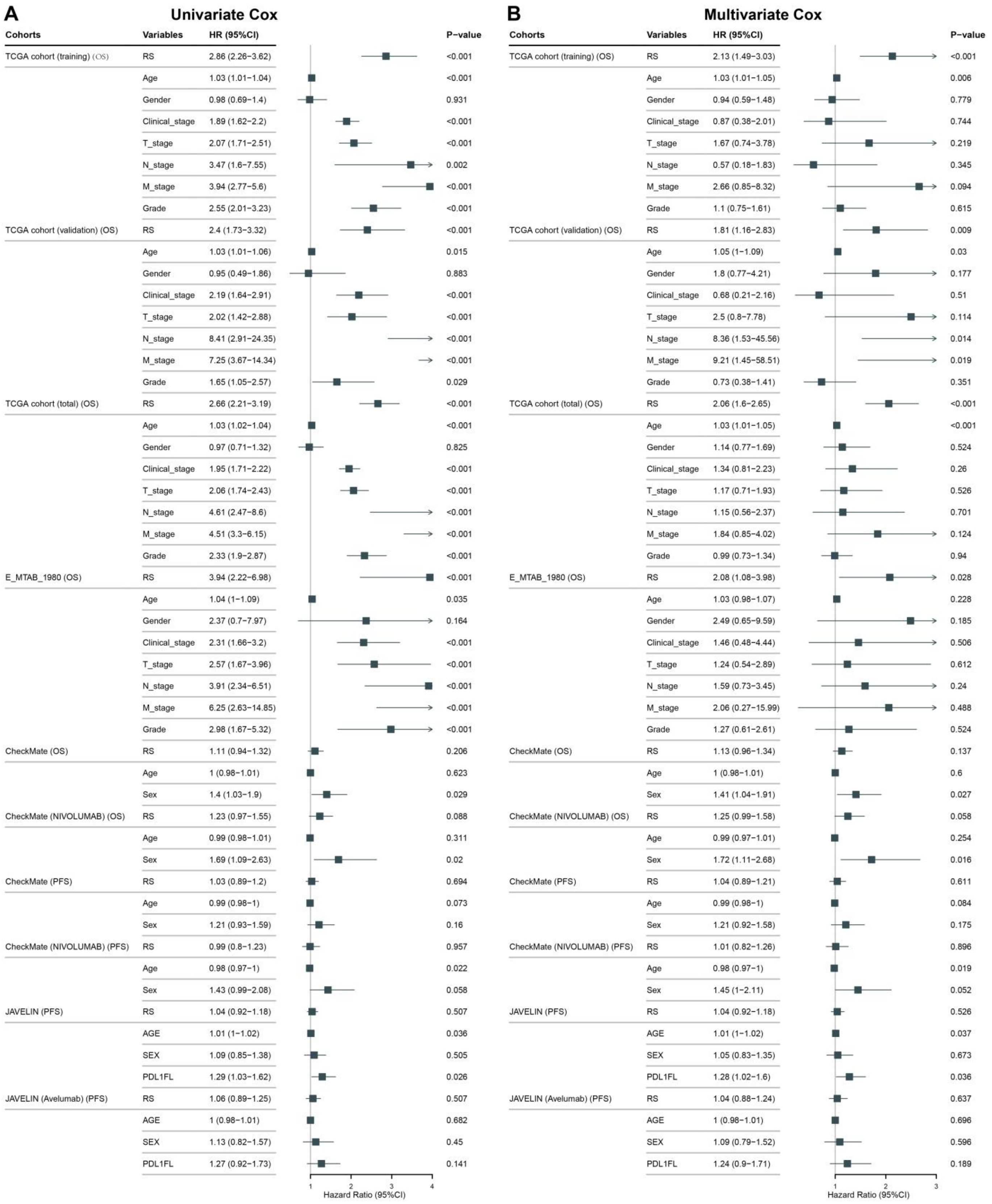

Figure S25 Forest plots of univariable and multivariable Cox regression analyses for the prognostic model across multiple cohorts.

(A-B) The prognostic value of the risk score (RS) derived from the model and key clinical variables was assessed in the TCGA, E-MTAB-1980, CheckMate (OS: overall survival), CheckMate (PFS: progression-free survival), and JAVELIN cohorts. For each cohort, panel (A) shows the univariable analysis results, and panel

(B) shows the multivariable analysis results. Hazard ratios (HRs) with 95% confidence intervals (CIs) are displayed both graphically (squares and horizontal lines) and numerically. The vertical grey line represents the null effect (HR = 1). A HR > 1 indicates a worse prognosis (risk factor), while an HR < 1 indicates a better prognosis (protective factor). Analyses were performed using the survival package in R. The specific statistical methods and sample sizes for each cohort are provided in the Methods section. Abbreviations: CI, confidence interval; HR, hazard ratio; OS, overall survival; PFS, progression-free survival.

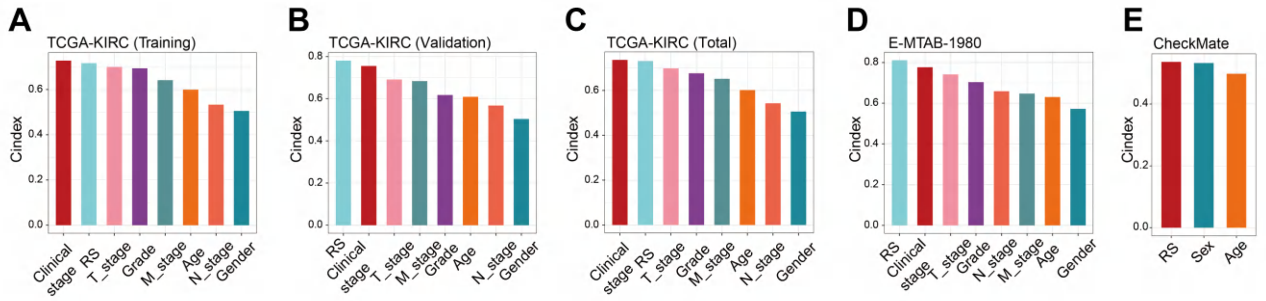

Figure S26 Validation and evaluation of ISM-EMTRS prognostic potentials.

(A-E) Histograms show the comparisons between performance of ISM-EMT- RS and other clinical variables in predicting prognosis.

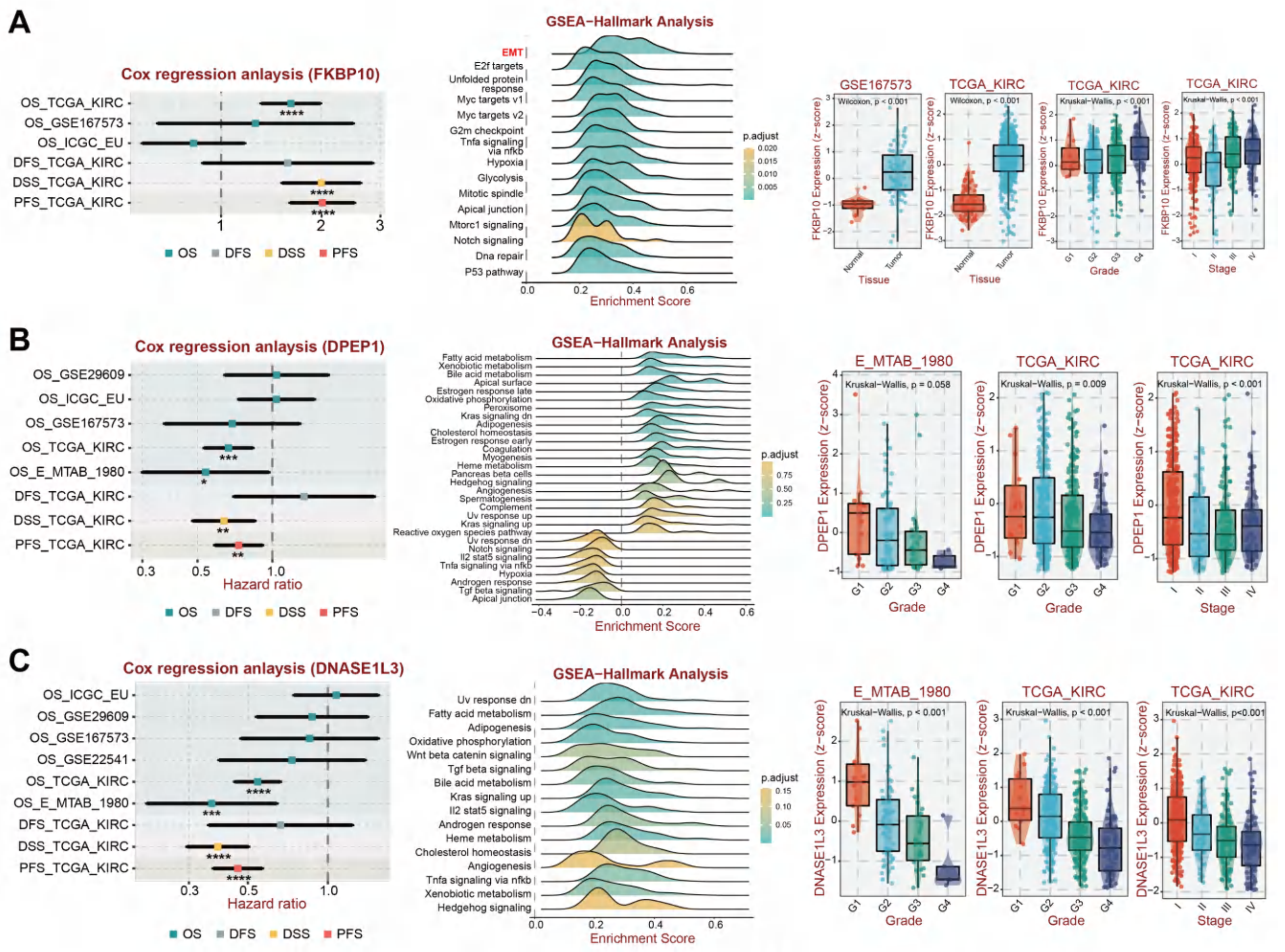

Figure S27 Validation of the role of FKBP10, DPEP1 and DNASE1L3 in ccRCC across 6 cohorts.

(A-C) (Left) Cox regression analysis revealed significant associations between the expressions of FKBP10, DPEP1, and DNASE1L3 with clinical outcomes such as Overall Survival (OS), Disease-Free Survival (DFS), Disease-Specific Survival (DSS), and Progression-Free Survival (PFS) across datasets like GSE29609 (n = 39), ICGC_EU (n = 91), GSE167573 (n = 63), E-MTAB-1980 (n = 101), GSE22541 (n = 68) and TCGA_KIRC (n = 533). (Middle) GSEA-Hallmark analysis identified key pathways enriched in correlation with these genes, including epithelial-mesenchymal transition, fatty acid metabolism and oxidative phosphorylation, and. (Right) Boxplots showed increase expression of FKBP10 and decreased expression of DNASE1L3, DPEP1 from early to advanced clinical stages or pathological grade. Significance was assessed using the Kruskal-Wallis test (multi-group comparisons) and Wilcoxon test (pairwise comparisons). All statistical tests were two-sided, $P < 0.05$.

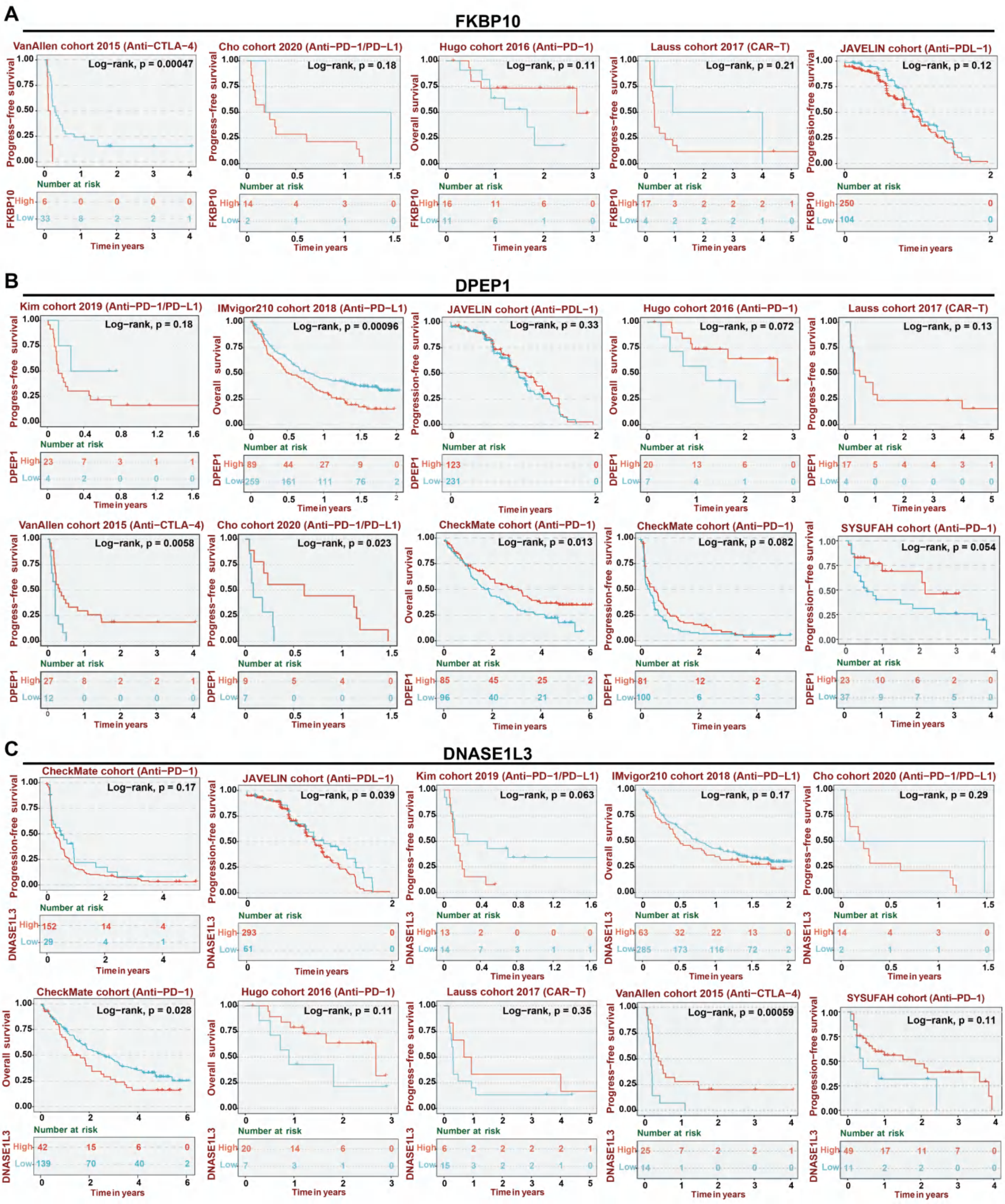

Figure S28 Association of FKBP10, DPEP1, and DNASE1L3 expression with survival outcomes in immunotherapy cohorts.

(A-C) Kaplan-Meier curves illustrate progression-free survival (PFS) and overall survival (OS) for patients across multiple cohorts treated with immune checkpoint inhibitors. Patients were stratified into high- and low-expression groups based on the optimal cut-off value for each gene (FKBP10, DPEP1, DNASE1L3). The log-rank test was used to compare survival differences between groups.

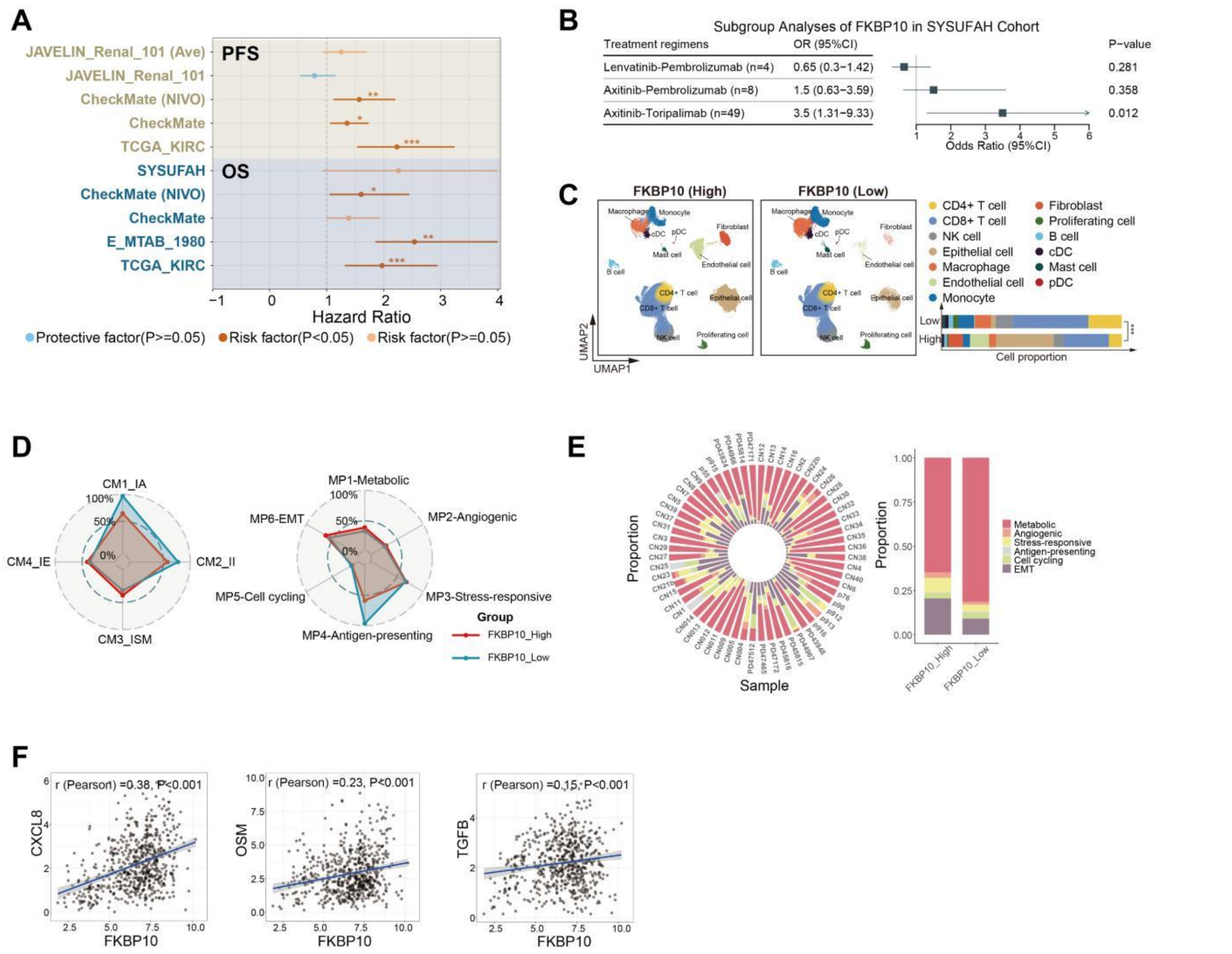

Figure S29 FKBP10 is an independent prognostic factor and remodels the tumor immune microenvironment in ccRCC.

(A) Multivariable Cox regression forest plot demonstrating that high expression of FKBP10 is an independent risk factor in ccRCC patients across the TCGA-KIRC, E-MTAB-1980, CheckMate, and JAVELIN cohorts. Analyses were adjusted for age, gender, clinical stage, pathological stage, and PD-L1 status. (B) Subgroup analysis of an independent validation cohort (SYSUFAH, n = 61) receiving different immunotherapy regimens. FKBP10 was a significant risk factor in the Axitinib plus Toripalimab treatment group (n = 49). No significant association (P > 0.05) was observed in the Lenvatinib plus Pembrolizumab (n = 4) or Axitinib plus Pembrolizumab (n = 8) groups, likely due to limited sample size. (C) UMAP visualization of tumor-infiltrating immune cells (External validation in Bi, Li, and Obradovic datasets, cells = 429,854) with FKBP10-high/-low grouping. Bar plot (right) showed the specific cell proportion of FKBP10-high/-low groups (χ^2 test, P < 0.001). (D-E) Analysis of TIME subtypes and malignant cell states in the independent external validation cohort (n = 62). (D) The proportion of antitumor TIME subtypes (CM1-IA and CM2-II) was significantly reduced, while the immunosuppressive CM3-ISM subtype was significantly increased in FKBP10-High tumors. (E) The proportion of tumor cells in the low-malignancy MP1-Metabolic state was decreased, whereas the EMT state was significantly increased in the FKBP10-High group (Chi-square test, P < 0.05 for all comparisons). (F)

Correlation analysis between FKBP10 expression and cytokine levels (CXCL8, TGFB, OSM). FKBP10 expression showed the strongest positive correlation with CXCL8 among the cytokines tested.

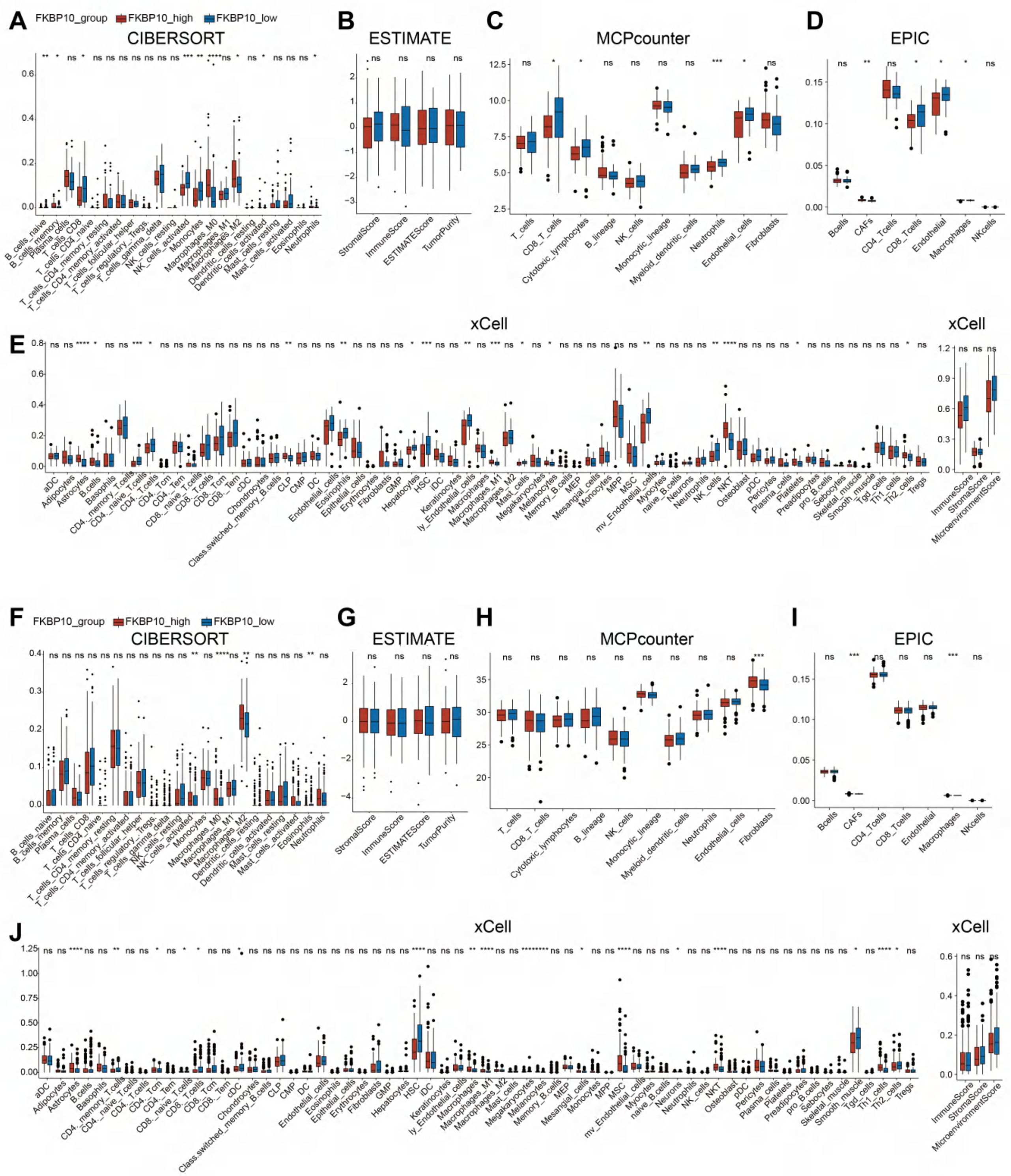

Figure S30 Analysis of immune cell infiltration stratified by FKBP10 expression levels in E-MTAB-1980 and Checkmate cohorts.

(A-E) The tumor immune microenvironment was evaluated using multiple algorithms (CIBERSORT, ESTIMATE, MCPcounter, EPIC, xCell) in E-MTAB-1980. Patients within the cohort were stratified into high and low FKBP10 expression groups. In the E-MTAB-1980 cohort, high FKBP10 expression was associated

with a significant decrease in T cells and an increase in macrophages (particularly M2 macrophages) and cancer-associated fibroblasts (CAFs). (F-J) The tumor immune microenvironment was evaluated using multiple algorithms in Checkmate cohort. In the Checkmate cohort, high FKBP10 expression was associated with a significant decrease in T cells and an increase in M2 macrophages, as well as CAFs. Data are presented as mean ± SD. Statistical significance was determined by the Wilcoxon rank-sum test (*$P < 0.05$, **$P < 0.01$,***$P < 0.001$).

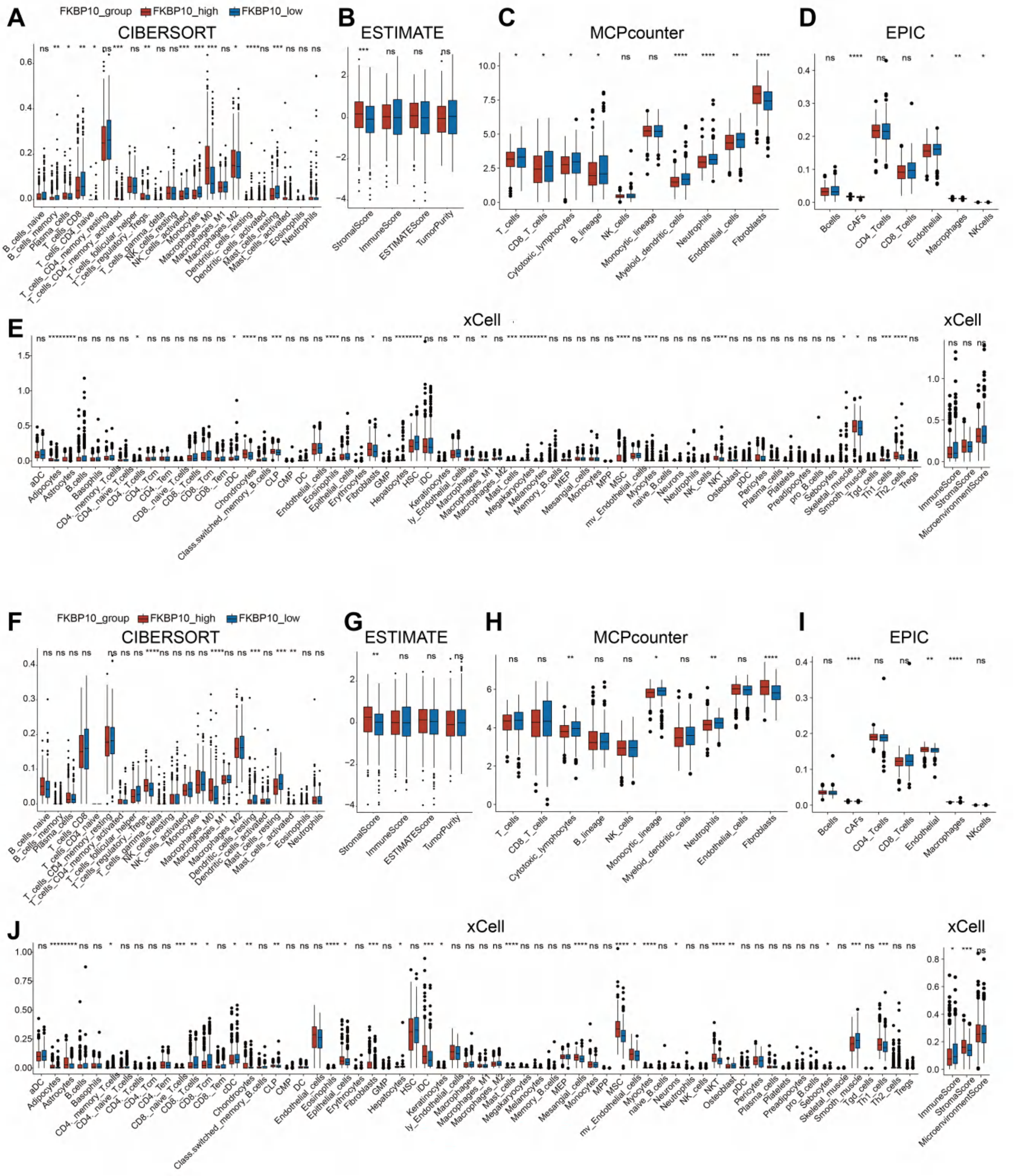

Figure S31 Analysis of immune cell infiltration stratified by FKBP10 expression levels in TCGA-KIRC

and JAVELIN cohorts.

(A-E) The tumor immune microenvironment was evaluated using multiple algorithms in TCGA-KIRC cohort. In the TCGA-KIRC cohort, the high FKBP10 group similarly showed reduced T cells, increased M2 macrophages and CAFs, and a significantly elevated stromal score. (F-J) The tumor immune microenvironment was evaluated using multiple algorithms in JAVELIN cohort. In the JAVELIN cohort, the high FKBP10 group exhibited T cell reduction, increased CAFs, a significantly decreased immune score, and a significantly increased stromal score. Data are presented as mean ± SD. Statistical significance was determined by the Wilcoxon rank-sum test (**P* < 0.05, ***P* < 0.01,****P* < 0.001).

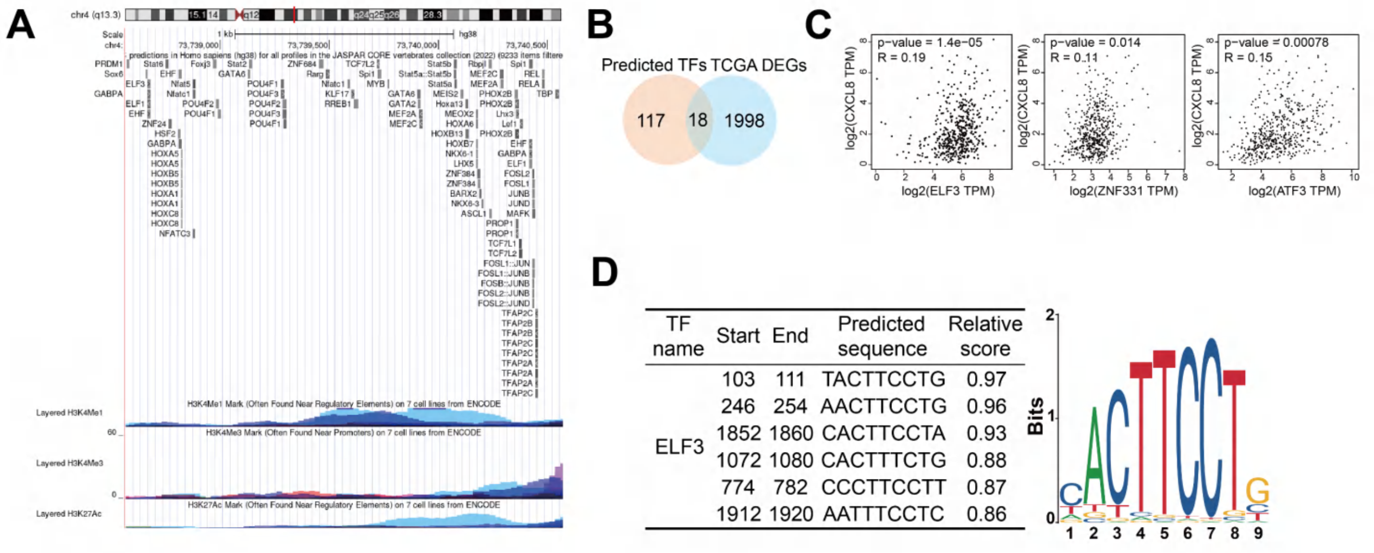

Figure S32 Analysis of transcription factor binding and prioritization of a key regulator at the CXCL8 locus.

(A) Analysis of ENCODE ChIP-seq data using MACS2 (q < 0.01) identifies 135 transcription factors (TFs). These TFs demonstrate coordinated occupancy at chromatin domains marked by active promoters (H3K4me3+/H3K27ac+) and putative enhancers (H3K4me1+) within the CXCL8 locus, spanning -2000 bp to +100 bp surrounding the transcription start site (TSS). (B) Venn intersection (n = 18 genes) between TCGA differentially expressed genes (|log2FC| > 1, FDR < 0.05) and predicted TFs. (C) Among these 18 genes, ELF3, ZNF331 and ATF3 demonstrate significant co-expression with CXCL8 (*P* < 0.001). (D) JASPAR analysis prioritizes ELF3 as the dominant regulator, showing both high binding potential (relative score > 0.85) and conserved motif at the CXCL8 promoter (right panel).

Reference

1. Zhang Y, Narayanan SP, Mannan R, Raskind G, Wang X, Vats P, et al. Single-cell analyses of renal cell cancers reveal insights into tumor microenvironment, cell of origin, and therapy response. Proc Natl Acad Sci U S A. 2021; 118.
2. Stewart BJ, Ferdinand JR, Young MD, Mitchell TJ, Loudon KW, Riding AM, et al. Spatiotemporal

immune zonation of the human kidney. Science. 2019; 365: 1461-6.
3. Coorens THH, Treger TD, Al-Saadi R, Moore L, Tran MGB, Mitchell TJ, et al. Embryonal precursors of Wilms tumor. Science. 2019; 366: 1247-51.
4. Melé M, Ferreira PG, Reverter F, DeLuca DS, Monlong J, Sammeth M, et al. Human genomics. The human transcriptome across tissues and individuals. Science. 2015; 348: 660-5.
5. McGinnis CS, Murrow LM, Gartner ZJ. DoubletFinder: Doublet Detection in Single-Cell RNA Sequencing Data Using Artificial Nearest Neighbors. Cell Syst. 2019; 8: 329-37.e4.
6. Zheng L, Qin S, Si W, Wang A, Xing B, Gao R, et al. Pan-cancer single-cell landscape of tumor-infiltrating T cells. Science. 2021; 374: abe6474.
7. Gulati GS, Sikandar SS, Wesche DJ, Manjunath A, Bharadwaj A, Berger MJ, et al. Single-cell transcriptional diversity is a hallmark of developmental potential. Science. 2020; 367: 405-11.
8. Yoshihara K, Shahmoradgoli M, Martínez E, Vegesna R, Kim H, Torres-Garcia W, et al. Inferring tumour purity and stromal and immune cell admixture from expression data. Nat Commun. 2013; 4: 2612.
9. Newman AM, Liu CL, Green MR, Gentles AJ, Feng W, Xu Y, et al. Robust enumeration of cell subsets from tissue expression profiles. Nat Methods. 2015; 12: 453-7.
10. Becht E, Giraldo NA, Lacroix L, Buttard B, Elarouci N, Petitprez F, et al. Estimating the population abundance of tissue-infiltrating immune and stromal cell populations using gene expression. Genome Biol. 2016; 17: 218.
11. Racle J, de Jonge K, Baumgaertner P, Speiser DE, Gfeller D. Simultaneous enumeration of cancer and immune cell types from bulk tumor gene expression data. Elife. 2017; 6.
12. Aran D, Hu Z, Butte AJ. xCell: digitally portraying the tissue cellular heterogeneity landscape. Genome Biol. 2017; 18: 220.
